# First fossil-leaf floras from Brunei Darussalam show dipterocarp dominance in Borneo by the Pliocene

**DOI:** 10.7717/peerj.12949

**Published:** 2022-03-24

**Authors:** Peter Wilf, Xiaoyu Zou, Michael P. Donovan, László Kocsis, Antonino Briguglio, David Shaw, JW Ferry Slik, Joseph J. Lambiase

**Affiliations:** 1 Department of Geosciences and Earth & Environmental Systems Institute, Pennsylvania State University, University Park, Pennsylvania, United States; 2 Department of Paleobotany and Paleoecology, Cleveland Museum of Natural History, Cleveland, Ohio, United States; 3 Faculty of Science, Universiti Brunei Darussalam, Gadong, Brunei Darussalam; 4 Institute of Earth Surface Dynamics, Faculty of Geosciences and Environment, University of Lausanne, Lausanne, Switzerland; 5 Dipartimento di Scienze della Terra, dell’Ambiente e della Vita, Università degli Studi di Genova, Genoa, Italy; 6 Biostratigraphic Associates (UK) Ltd., Stoke-on-Trent, UK; 7 Lambiase Geoscience, Nokomis, Florida, United States

**Keywords:** Paleobotany, Borneo, Pliocene, Pleistocene, Dipterocarpaceae, Melastomataceae, Araceae, Rhamnaceae, Ferns, Palynology

## Abstract

The Malay Archipelago is one of the most biodiverse regions on Earth, but it suffers high extinction risks due to severe anthropogenic pressures. Paleobotanical knowledge provides baselines for the conservation of living analogs and improved understanding of vegetation, biogeography, and paleoenvironments through time. The Malesian bioregion is well studied palynologically, but there have been very few investigations of Cenozoic paleobotany (plant macrofossils) in a century or more. We report the first paleobotanical survey of Brunei Darussalam, a sultanate on the north coast of Borneo that still preserves the majority of its extraordinarily diverse, old-growth tropical rainforests. We discovered abundant compression floras dominated by angiosperm leaves at two sites of probable Pliocene age: Berakas Beach, in the Liang Formation, and Kampong Lugu, in an undescribed stratigraphic unit. Both sites also yielded rich palynofloral assemblages from the macrofossil-bearing beds, indicating lowland fern-dominated swamp (Berakas Beach) and mangrove swamp (Kampong Lugu) depositional environments. Fern spores from at least nine families dominate both palynological assemblages, along with abundant fungal and freshwater algal remains, rare marine microplankton, at least four mangrove genera, and a diverse rainforest tree and liana contribution (at least 19 families) with scarce pollen of Dipterocarpaceae, today’s dominant regional life form. Compressed leaves and rare reproductive material represent influx to the depocenters from the adjacent coastal rainforests. Although only about 40% of specimens preserve informative details, we can distinguish 23 leaf and two reproductive morphotypes among the two sites. Dipterocarps are by far the most abundant group in both compression assemblages, providing rare, localized evidence for dipterocarp-dominated lowland rainforests in the Malay Archipelago before the Pleistocene. The dipterocarp fossils include winged *Shorea* fruits, at least two species of plicate *Dipterocarpus* leaves, and very common *Dryobalanops* leaves. We attribute additional leaf taxa to Rhamnaceae (*Ziziphus*), Melastomataceae, and Araceae (*Rhaphidophora*), all rare or new fossil records for the region. The dipterocarp leaf dominance contrasts sharply with the family’s <1% representation in the palynofloras from the same strata. This result directly demonstrates that dipterocarp pollen is prone to strong taphonomic filtering and underscores the importance of macrofossils for quantifying the timing of the dipterocarps’ rise to dominance in the region. Our work shows that complex coastal rainforests dominated by dipterocarps, adjacent to swamps and mangroves and otherwise similar to modern ecosystems, have existed in Borneo for at least 4–5 million years. Our findings add historical impetus for the conservation of these gravely imperiled and extremely biodiverse ecosystems.

## Introduction

The everwet rainforests of the Southeast Asian tropics are located in the Malesian bioregion, comprised of Peninsular Malaysia and the ca. 25,000 islands of the Malay Archipelago from Sumatra to New Guinea and the Philippines (van Steenis, 1950; Whitmore, 1984; Wikramanayake, Dinerstein & Loucks, 2002). Malesia’s forests rank among the most biodiverse on the planet, even as their species suffer exceptionally high extinction risks. Thus, the densely populated (ca. 400 million people) region has become an epicenter of urgent conservation threats, including wildlife trafficking, deforestation, climate change, fires, and pollution of air, water, and coastal ecosystems (Hoffmann et al., 2010; Bryan et al., 2013; de Bruyn et al., 2014; Slik et al., 2015; Crippa et al., 2016; Nater et al., 2017; Gaveau et al., 2018; Gaveau et al., 2021; Corlett, 2019; Tilker et al., 2019; Joyce et al., 2020; Raven et al., 2020; Jung et al., 2021). Knowledge of fossil history is a powerful conservation tool (*e.g*., Kooyman, Watson & Wilf, 2020) that increases public awareness of ecosystem heritage and fulfills evolutionary and geological history criteria for designation of UNESCO World Heritage Sites and other preservation areas worldwide (see https://whc.unesco.org/en/criteria). However, paleo-conservation is little developed in the region due to its comparatively limited fossil record (*e.g*., Lee, 1992; Brown et al., 2004; Zonneveld et al., 2011; van Gorsel, 2014; van Gorsel, 2020; Murray et al., 2015; Claude, 2017).

A significant distinction of Malesian from New World and African tropical rainforests is that their history is closely tied to tectonic introductions from exotic terranes (*e.g*., Hall, 1996; Hall, 2011; Hall, 2017; Morley, 2000; Lohman et al., 2011; Ashton, 2014). Since well before plate tectonic theory, the Malesian flora has been understood as an enormously complex juxtaposition of *in-situ* speciation and exchange with disparate sources, including Eurasia and the two Gondwana-sourced terranes that collided with Asia during the Cenozoic, the Indian and Australian plates (Stapf, 1894; van Steenis, 1934; van Steenis, 1964; van Steenis, 1971; van Steenis, 1979; Whitmore, 1981; Beaman & Beaman, 1990; Morley, 2018; Morley, 2000; Sniderman & Jordan, 2011; Ashton, 2014; Kooyman et al., 2019; Joyce et al., 2021). As a result, a great deal of paleobotanical (used here for macrofossils) knowledge about the origins of the Malesian rainforest comes, not from the undersampled area of Malesia itself, but from rapidly increasing fossil discoveries in the principal contributing areas and their formerly contiguous land masses: Eurasia, India, Australia, Antarctica, and South America (Hill, 1994; Hill & Brodribb, 1999; Jin et al., 2010; Wilf et al., 2013; Kooyman et al., 2014; Kooyman et al., 2019; Wheeler et al., 2017; Tarran et al., 2018; Manchester et al., 2019; Wu et al., 2019).

The celebrated history of biological exploration and research in Malesia began in the 17^th^ century with the expeditions of Georg Eberhard Rumphius (de Wit, 1952). Later, Alfred Russel Wallace’s seminal observations in the region formed the basis of the field of biogeography and his independent discovery of evolution by natural selection (Darwin & Wallace, 1858; Wallace, 1860; Wallace, 1869). Wallace (1869) posed an important, apparently overlooked conjecture by observing (italics ours) “the existence of extensive coal-beds in Borneo and Sumatra, of such recent origin that *the leaves which abound in their shales are scarcely distinguishable from those of the forests which now cover the country*.”

Nearly all recent paleobotanical work in Malesia comes from the Permian of Sumatra (Indonesia; van Waveren et al., 2018; van Waveren et al., 2021) and the Late Triassic of Bintan Island (Indonesia; Wade-Murphy & van Konijnenburg-van Cittert, 2008). Research on Cenozoic regional floras falls into three preservational domains: compression floras dominated by leaves, fossil woods, and pollen. Work on compression floras largely dates to the 19^th^ and early 20^th^ centuries, starting even before Wallace’s time, and covers material from the Paleogene of Sumatra (Indonesia; Heer, 1874; Heer, 1881) and South Kalimantan (Indonesia; Geyler, 1875; see also von Ettingshausen, 1883b); the Neogene of Sumatra (Kräusel, 1929a), Java (Indonesia; Göppert, 1854; von Ettingshausen, 1883a; Crié, 1888), and Labuan Island (offshore Malaysian Borneo; Geyler, 1887); and a few other areas (see summaries in Kräusel, 1929b; Bande & Prakash, 1986; van Gorsel, 2020). We are not aware of any significant revisions of these important early reports (van Konijnenburg-van Cittert, van Waveren & Jonkers, 2004). As for comparable works on compression floras from the discovery era elsewhere in the world (see Dilcher, 1971; Hill, 1982), we must assume that many of the historical identifications are inaccurate, pending restudy of the type collections (van Konijnenburg-van Cittert, van Waveren & Jonkers, 2004).

The body of work focused on Malesian fossil woods is more botanically informative than for compressions and includes Neogene records of many plant families that are extant in the region. The wood literature encompasses historical to comparatively recent studies of apparently *ex-situ* specimens from the Neogene of Sumatra, Borneo, and Java (Kräusel, 1922; Kräusel, 1926; den Berger, 1923; den Berger, 1927; Schweitzer, 1958; Kramer, 1974a; Kramer, 1974b; Mandang & Kagemori, 2004; see also Ashton, 1982; Wheeler, 2011).

The wood record includes numerous fossils of Dipterocarpaceae, which overwhelmingly dominate today’s lowland rainforests of the region and are thus central to most discussions of Asian rainforest evolution and biogeography (*e.g*., Maury-Lechon & Curtet, 1998; Morley, 2000; Ashton, 2014; Raes et al., 2014; Ghazoul, 2016). The age and biogeographic origins of the dipterocarps are widely debated (Ashton, 1982; Morley, 2003; Shukla, Mehrotra & Guleria, 2013; Ghazoul, 2016; Kooyman et al., 2019; Ashton et al., 2021; see Discussion). The family includes many extremely tall trees (Ashton, 1982; Shenkin et al., 2019), providing the characteristic vertical structure of regional rainforests that is famously associated with elevated animal diversity, including diverse vertebrate gliders (Dudley & DeVries, 1990; Heinicke et al., 2012); their long-cycle mast flowering and fruiting events are also a significant control on animal populations (*e.g*., Ashton, Givnish & Appanah, 1988; Corlett, 2019). The dipterocarps suffer severe anthropogenic pressure, especially from logging followed by agricultural conversion (Ashton, 2014; Corlett, 2019; Ashton et al., 2021; Bartholomew et al., 2021). Of 460 Asian dipterocarp species (subfamily Dipterocarpoideae) assessed in the IUCN Red List (IUCN, 2021), 408 (89%) have Near Threatened status or worse; 57% are Endangered, Critically Endangered, or Extinct. Borneo has 267 dipterocarp species, more than half the global total, of which 162 (61%) are endemic; 99 of those endemic species (62%) are threatened with extinction (Bartholomew et al., 2021).

In contrast to the limited macrofossil data, a great deal is known about Malesian rainforest history in the Cenozoic from decades of palynological research (*e.g*., Muller, 1964; Muller, 1966; Muller, 1968; Morley, 1982; Morley, 1998; Morley, 2000; Morley, 2002; Morley, 2003; Morley, 2018; van der Kaars, 1991; Morley & Jirin, 2006; Lelono & Morley, 2011; Witts et al., 2012; Morley & Morley, 2013; Morley & Morley, 2022). Compared with macrofossils, pollen assemblages sample a broader range of environments; they occur at a higher abundance and stratigraphic density but lower taxonomic, temporal, and spatial resolution (*e.g*., Behrensmeyer, Kidwell & Gastaldo, 2000). Pollen records paired with macrofossil assemblages from the same strata are a powerful combination that typically allows recognition of many more taxa than from either component taken separately (*e.g*., Barreda et al., 2020). Notably, dipterocarp pollen data reported from the region often, but not always (Demchuk & Moore, 1993; Morley, 2000; Morley, 2018; Dodson et al., 2019) show conspicuously low relative abundances, even as late as the Holocene (Maxwell, 2001; Penny, 2001; Morley et al., 2004; Rugmai et al., 2008; Sepulchre et al., 2010; Hamilton et al., 2019). The likely under-representation of dipterocarps in many pollen records has been related to taphonomic and life-history factors that minimize fossilization potential (Ashton, 1982; Bera, 1990; Maxwell, 2001; Sepulchre et al., 2010; Hamilton et al., 2019).

From the evidence at hand, there is a consensus that dipterocarps became dominant in everwet rainforests of the Malay Archipelago after about 20 Ma (Ashton, 1982; Morley, 2000; Heinicke et al., 2012). However, there has been no comparative use of compression floras in assessing past dipterocarp abundance. Compression floras often provide taxonomic resolution below the family level, and unbiased collections of fossil leaves are widely used in paleoecology to evaluate diversity and relative abundance at a far more local scale than is possible from pollen or *ex-situ* woods (*e.g*., Chaney & Sanborn, 1933; Burnham, 1994b; Wing & DiMichele, 1995; Wilf, 2000). Significantly, relative leaf area and leaf counts in modern litter samples correlate directly with relative stem basal area in source forests (Burnham, Wing & Parker, 1992; Burnham, 1994a; Burnham, 1997). Thus, leaf and pollen data from the same strata, when both are based on unbiased (“quantitative” or “census”) sampling, offer the opportunity to obtain complementary data about dominance and diversity from co-occurring macrofossils and microfossils (Nichols & Johnson, 2002; Barreda et al., 2020).

**Figure 1:**
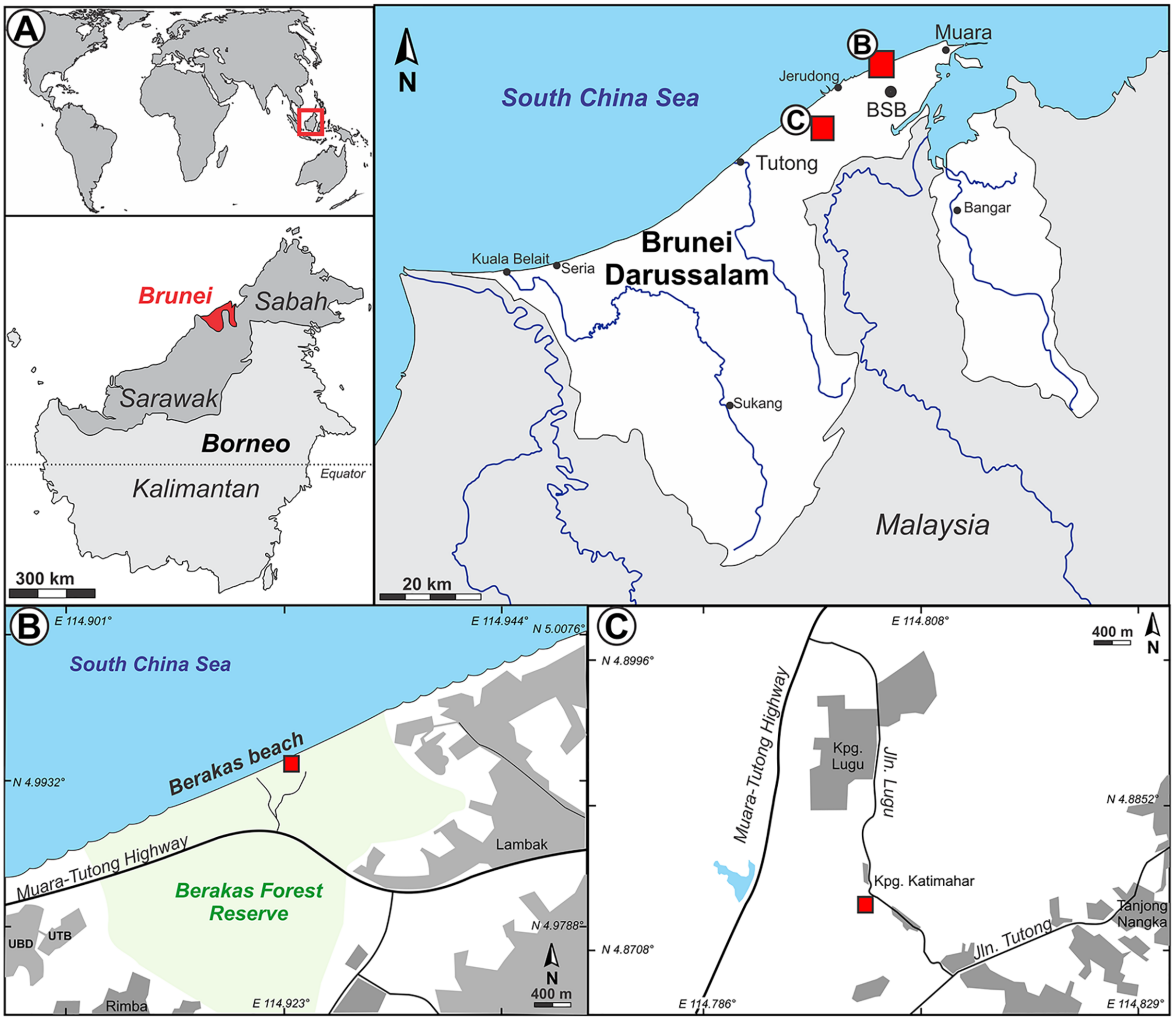
Study area. (A) Inset series showing location of Brunei Darussalam, the capital Bandar Seri Begawan (BSB), and the two fossil sites studied here at Berakas Beach (B), detailed in Fig. 2, and Kampong Lugu (C), detailed in Fig. 3.

The Sultanate of Negara Brunei Darussalam (informally, Brunei; Fig. 1) is a jewel of tropical biodiversity conservation in Borneo, the Malay Archipelago, and the world. Over half of Brunei’s forest remains unlogged, compared with only ca. 3% of intact forest cover remaining in the neighboring Malaysian state of Sarawak (Bryan et al., 2013). In a total land area similar to that of Delaware, USA, Brunei has about 3,500 cataloged seed-plant species, including almost 200 species of Dipterocarpaceae; the actual species richness is presumed to be much higher (Ashton, 1964; Ashton, 1982; Coode et al., 1996; Wong & Kamariah, 1999; Ashton, Kamariah & Said, 2003). There was, until this report, no previous paleobotanical (macrofossil) record from Brunei. Palynological data from Brunei was also scarce until recently (Germeraad, Hopping & Muller, 1968; Anderson & Muller, 1975; see Roslim et al., 2021 for additional regional literature), and many early studies were never published (see Morley, 1991). However, Roslim et al. (2021) recently reported diverse and well-illustrated palynofloras studied under SEM, comprising 62 taxa from 37 families sampled from a suite of 36 Miocene and Pliocene outcrops in Brunei. Dipterocarp pollen was reported as rare, consisting of two species of *Shorea* from Miocene sediments. From many of the same Miocene to Pliocene outcrops, Kocsis et al. (2020) sampled ambers, finding from spectral analyses that all samples came from dipterocarp source plants and none from other resiniferous taxa in the region such as *Agathis* (Araucariaceae).

**Figure 2:**
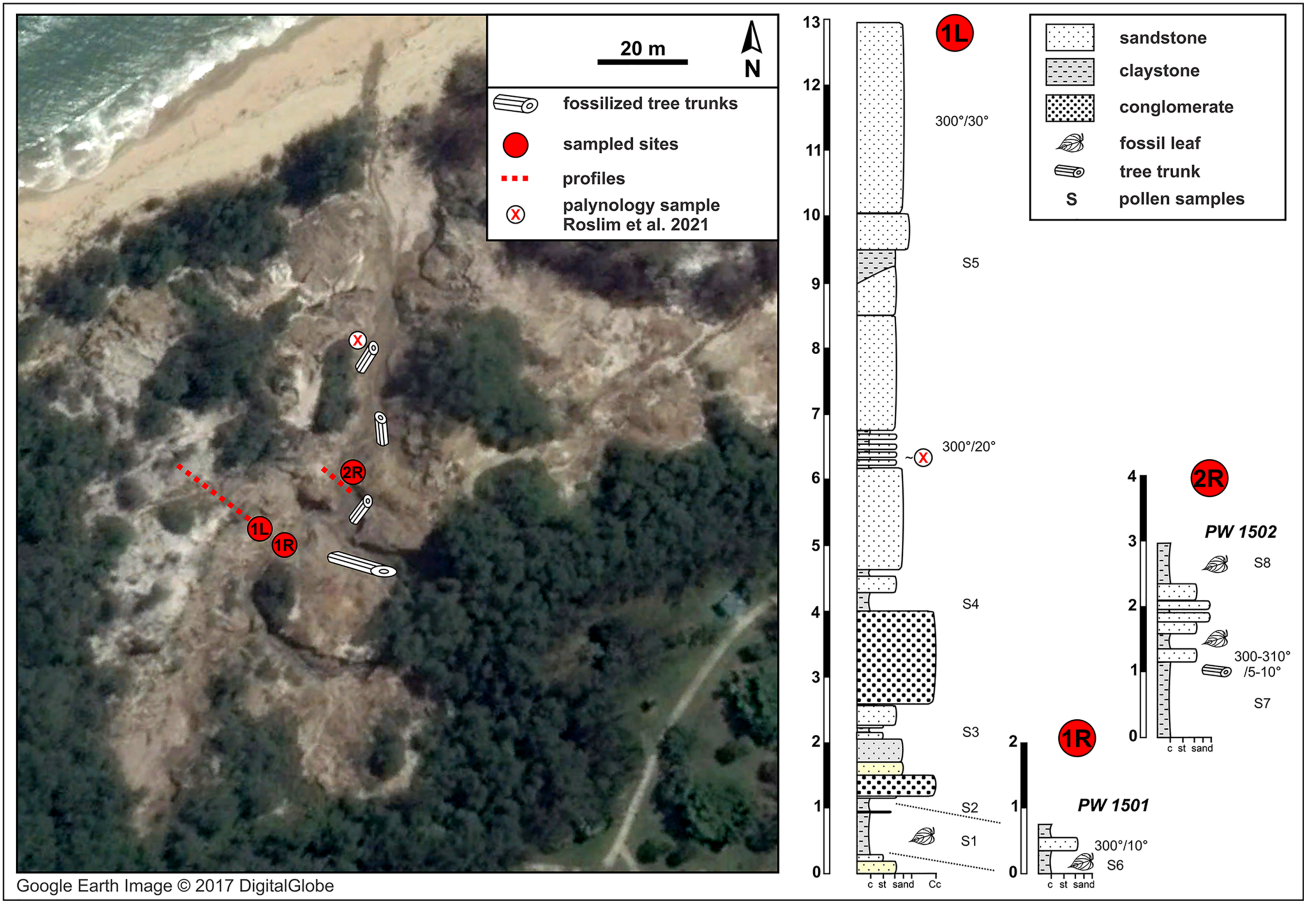
Berakas Beach fossil site (see also Fig. 1) and stratigraphic sections. Fossil locality PW1501 comes from a single horizon that crops out on both sides of a small gully (sections 1L and 1R), and locality PW1502 is from section 2R. Google Earth Image © 2017 DigitalGlobe.

**Figure 3:**
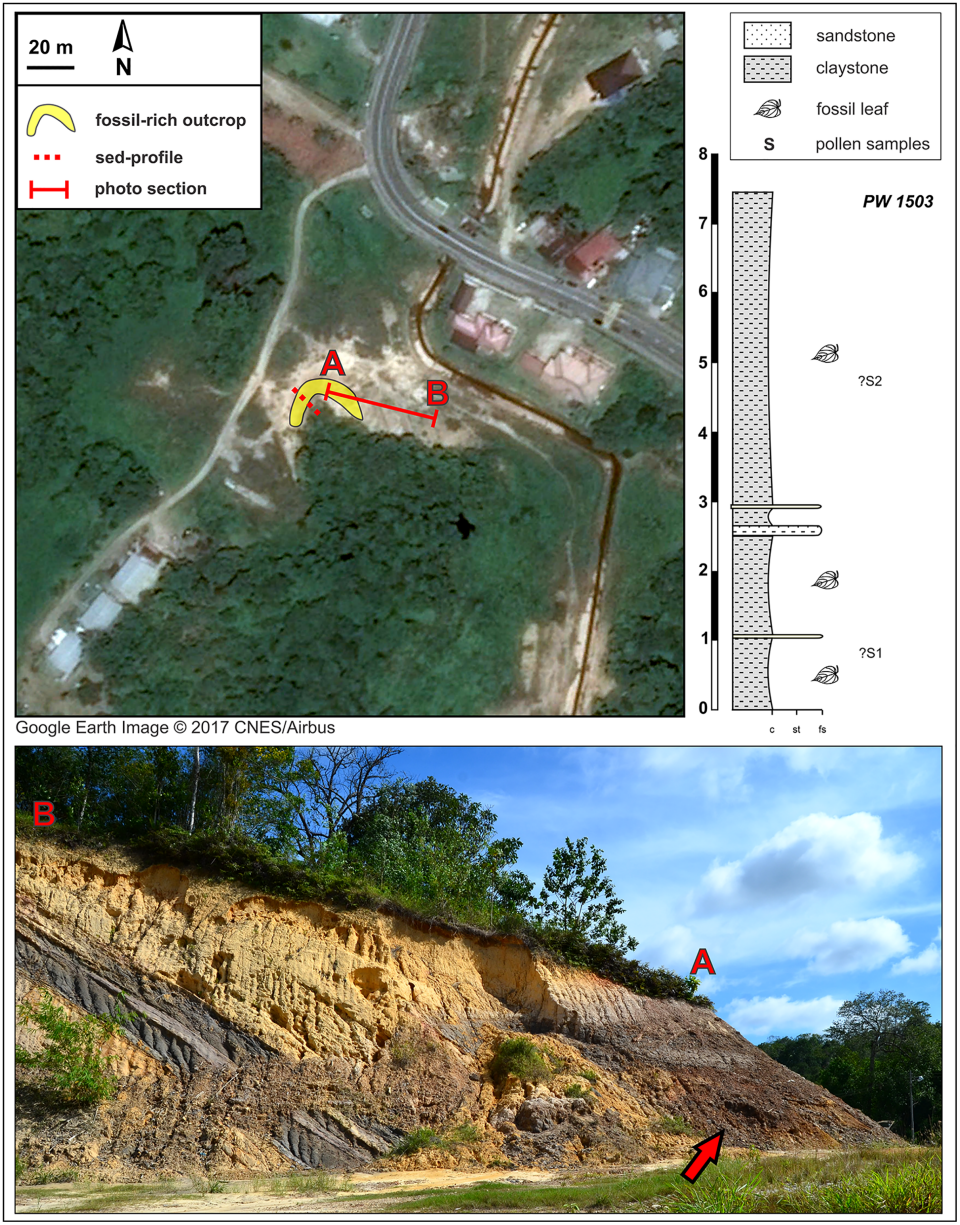
Kampong Lugu fossil site (see also Fig. 1) and stratigraphic section. A sharp, ca. 30° angular unconformity separates the lower unit, the Miocene Miri Formation, from the horizontal, dark, onlapping beds of the new stratigraphic unit, which is exposed in a horseshoe around the west and north sides of the local hill as shown (marker A; bottom photograph shows the north face of the outcrop). Fossil leaves are abundant throughout the dark claystones. Google Earth Image © 2017 CNES/Airbus.

Long ago, Geyler (1887) reported a small Miocene leaf flora from the shallow marine Belait Formation on the small Malaysian island of Labuan, located just north of Brunei near the mouth of Brunei Bay, from a site that is no longer accessible. From this lead, we decided to launch a field season of paleobotanical reconnaissance in the Belait Formation in Brunei, where it crops out at many locations (Back et al., 2001; Lambiase & Tulot, 2013; Roslim et al., 2021), and in other Neogene strata with potential to hold plant remains (Kocsis et al., 2020; Roslim et al., 2020; Roslim et al., 2021). This report details our discovery, preliminary descriptions, and interpretations of abundant fossils from two different compression-flora sites that are the first from Brunei, along with palynological samples from the same beds as the leaf fossils.

## Materials and Methods

Our field survey in May–June 2015 covered numerous late Cenozoic natural and anthropogenic outcrops of the Setap Shale (early to middle Miocene), Belait (early to late Miocene), Miri (middle-late Miocene), Seria (late Miocene), and Liang (latest Miocene-early Pliocene) formations in the western portion of Brunei (Tutong and Brunei-Muara districts). At the times of deposition of these formations, what is now Borneo was a partially elevated area of eastern Sundaland, with overland connections to mainland Southeast Asia (*e.g*., Hall, 2017; Morley, 2018). The localities visited were primarily the same ones that Kocsis et al. (2020) and Roslim et al. (2021) sampled for amber and palynological content, respectively; those authors provided maps and updated stratigraphic relationships of the units (see also Roslim et al., 2020). Almost all outcrops visited had abundant but degraded or hashy compressed plant remains, primarily of twigs and small leaf fragments with little potential for identification, as noted in various geological studies (Tate, 1976; Kocsis et al., 2018). We found and excavated macrofossils suitable for larger-scale collection at two localities, Berakas Beach and Kampong Lugu (Figs. 1–3), and we collected pollen samples from the freshest available sediment in the leaf-rich horizons. Fieldwork and collecting took place with permission from the Ministry of Industry and Primary Resources, Brunei Darussalam (reference [112]/JPH/UND/17PT.1, issued 25 May 2015).

### Berakas Beach locality

The natural fossiliferous outcrop at Berakas Beach is located in the gullies and creeks that cut the sea cliffs running along the coast in the Berakas Forest Reserve, next to the Muara-Tutong highway (Figs. 1, 2). The outcrop exposes rocks belonging to the Berakas Member of the Liang Formation (Sandal, 1996) that top up the core of the Berakas syncline (*e.g*., Morley et al., 2003). The age of the Liang Formation has been proposed as Pliocene and the youngest beds possibly Pleistocene, based on the late Miocene age of the underlying Seria Formation and the overlying Pleistocene terraces (Liechti et al., 1960; Wilford, 1961); however, some reports stretch the lower age limit to the latest Miocene (Sandal, 1996).

The Liang Formation overlies late Miocene, dominantly marine successions that crop out in the coastal areas of northern Borneo with locally varying depositional settings (Liechti et al., 1960; Sandal, 1996; Wannier et al., 2011). The sediments included in the Berakas syncline were deposited in a protected embayment and partly influenced by tidal processes; however, the younger Liang Formation strata are dominated by deposits of meandering rivers that filled up the bay and cut through the tidal and distributary sediments (Wannier et al., 2011). At the base of these river deposits, conglomeratic channel-lag beds containing fossil wood (not studied here) are well exposed due to recent weathering and erosion that formed gullies and small canyons in the area (Fig. 2). Otherwise, the lithology is dominated by fine to medium-grained sandstone that is often cross-bedded and intercalated claystone beds with high organic content and leaf compressions. Each sedimentary unit yielded amber fragments that have dipterocarp origin (Kocsis et al., 2020); pollen from these outcrops, at a somewhat higher stratigraphic position than the pollen samples studied here (marked in Fig. 2), was recently analyzed as part of a separate study (Roslim et al., 2021: their sample 16).

The fluvial layers in the investigated Berakas outcrops dip northwest at 5–30°. The ca. 13 m of logged strata contain compressed leaves and rare reproductive plant fossils at their clay-rich base (Fig. 2). Plant fossils were sampled from the locations shown in Fig. 2 as logs 1L and 1R, collectively as locality PW1501 (N 4.99404°, E 114.92297°), all from a single layer exposed on opposite banks of a small gully. About 30 m to the northeast (Fig. 2: log 2R) sits fossil locality PW1502 (N 4.99423°, E 114.92316°), where a similar clay-rich succession crops out with variable, sandier intercalations. Most of the section has sedimentary structures that point to deposition in a river system characterized by episodic increases of water flows. The presence of tree trunks, often fully embedded into clay deposits, indicates elevated bed and suspended loads. The sandier portions with cross stratifications and abundant reactivation surfaces point to calmer conditions that could have alternated between anastomosing and meandering fluvial systems. This variety of environmental conditions is plausible, considering the general likelihood of significant runoff events in the wet tropics. Possibly, the fossil-leaf-rich clay layers accumulated as part of fine-grained crevasse splay deposits. Several clay samples were taken, and one was processed from each of the two macrofossil localities for palynological study of age, paleoenvironment, and floral composition (see Palynology).

### Kampong Lugu locality

The Kampong Lugu fossil locality is situated southwest of Jerudong on the eastern flank of the Belait syncline (*e.g*., Sandal, 1996; also the western flank of the Jerudong anticline), and the fossiliferous strata are located in a new lithologic unit that we discovered, exposed mainly due to excavation (Figs. 1, 3). At the site (Fig. 3), the late Miocene marine sediments of the Miri Formation are exposed and dip northwest at 30° (*e.g*., Wilford, 1961). The age of the Miocene sediment series ranges from 10–12 Ma (east-southeast of the site; Back et al., 2005) to 6–8 Ma (north-northeast of the site, near Tutong; Kocsis et al., 2018; Roslim et al., 2020).

The new fossiliferous unit, a 7–7.5 m thick claystone, onlaps the Miocene marine series horizontally, showing a sharp, 30° angular unconformity (Fig. 3) that presumably represents a significant hiatus in the depositional system. A much younger, possibly Plio-Pleistocene age of the fossiliferous claystone is likely on that basis alone. The claystone is grey and sometimes light brown, relatively uniform but with several alternations and intercalations of sandy lenses toward the lower part of the section. The claystone is rich in leaf compressions at several levels, although no reproductive structures were found. The entire exposure was sampled opportunistically as a single fossil locality, PW1503 (N 4.87582°, E 114.80229°), along with two pollen samples (see Palynology). Bioturbation is very rare in the fossiliferous unit. The presence of thin, sandy layers could represent occasional, low-energy fluvial input, but there are no sedimentary structures indicating major fluvial forms such as channels, meanders, and oxbow lakes.

### Palynology

From the leaf-bearing horizons at each site, we took several pollen samples from the freshest rock available, of which two were processed per site. At Berakas Beach, one sample each was processed from fossil localities PW1501 (Fig. 2: sample S6) and PW1502 (Fig. 2: sample S8), and at Kampong Lugu locality PW1503, samples S1 and S2 came from the respective positions shown in Fig. 3. The samples were processed using standard palynological techniques. Approximately 20 g of a crushed, washed, and dried sample were first reacted with 20% hydrochloric acid to dissolve and disaggregate the carbonates. Once all chemical reactions had ceased, the sample was neutralized with water, then reacted with hydrofluoric acid (40%) to dissolve and disaggregate the silicates. The sample was then sieved with a 10 µm mesh nylon sieve, using water to neutralize. Any fluoride precipitates were removed by warming the residue in 20% hydrochloric acid, then re-sieving the samples with water to neutralize. The now-concentrated organic fraction was examined under the microscope to assess the need for oxidation using concentrated nitric acid or for mechanical separation using ultrasonic vibration. Representatives of the oxidized and unoxidized organic fractions were mounted onto cover slips, and these were glued to glass slides using Norland Optical Adhesive No. 63.

**Figure 4:**
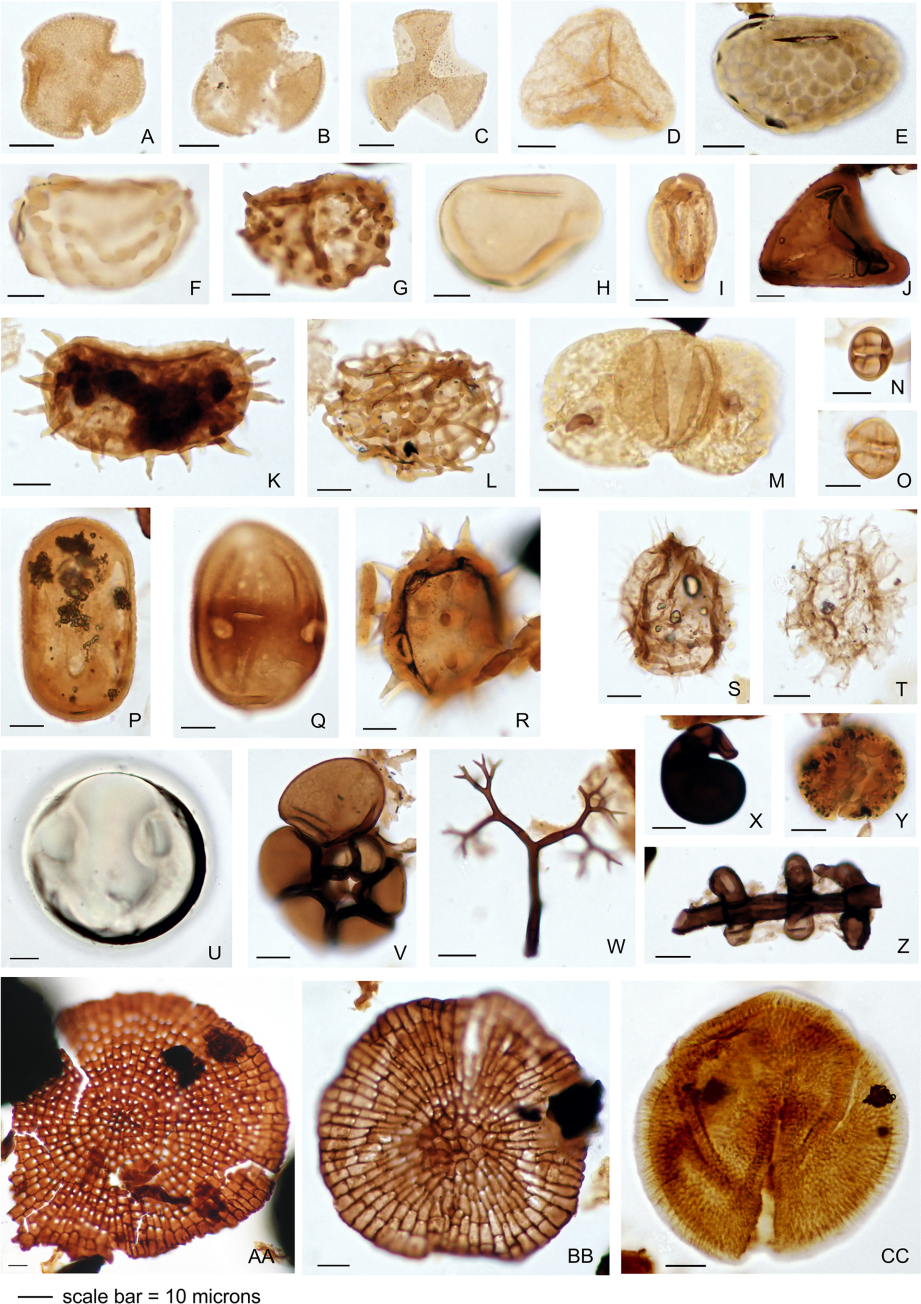
Representative palynomorph specimens from Berakas Beach (BE) and Kampong Lugu (KL). (A) *Discoidites* sp. (BE), affinity Malvaceae; (B, C) Dipterocarpaceae sp. (BE); (D) *Acrostichum* sp. (KL, Pteridaceae); (E) *Verrucatosporites favus* (BE, *Polypodium*); (F) *Stenochlaenidites papuanus* (BE, *Stenochlaena*); (G) *Verrucatosporites usmensis* (BE, *Stenochlaena*); (H) *Laevigatosporites* sp. (BE, Thelypteridaceae?); (I) *Rostriapollenites robustus* (BE, *Barringtonia*); (J) *Pteris* sp. (BE, Pteridaceae); (K) *Scolocyamus magnus* (BE, *Stenochlaena*); (L) *Praedapollis* sp. (BE, Fabaceae?); (M) *Podocarpus* sp. (BE, Podocarpaceae); (N, O) *Zonocostites ramonae* (KL, *Rhizophora*); (P) *Florschuetzia levipoli* (KL, *Sonneratia*); (Q) Sapotaceae sp. (BE); (R) *Nypa* sp. (KL, Arecaceae); (S) *Impletosphaeridium* sp. (KL, Dinoflagellata); (T) *Spiniferites* sp. (BE, Dinoflagellata); (U) *Tasmanites* sp. (BE, Prasinophyceae); (V) Foraminifera sp., test lining (KL); (W) *Dendromyceliates splendus* (KL, Agonomycetes); (X) *Cirrenalia pygmea* (KL, Helicospores); (Y) *Botryococcus* sp. (BE, Botryococcaceae); (Z) *Meliolinites* sp. (KL, Sordariomycetes); (AA) *Callimothallus* sp. (BE, microthyriaceous fungi); (BB) *Phragmothyrites* sp. (BE, microthyriaceous fungi); (CC) *Perfotricolpites digitatus* (KL, *Merremia*).

The palynology samples were logged quantitatively (Appendix 1). Palynomorph recovery in all samples proved very high, and it was possible to make counts of 300 specimens for each, after which the slides were scanned for additional taxa logged as presence-absence (Appendix 1). The remaining cover slips were observed for other significant taxa. All palynomorph groups were recorded, including spores, pollen, freshwater algae, fungal bodies, and marine microplankton. A semi-quantitative assessment was also made of the kerogen, not intended as a detailed kerogen study but rather a determination of the main kerogen types and their derivation. Corel Draw (Corel, Ottawa, ON, Canada) was used to compose an illustration of light micrographs for representative palynomorphs (Fig. 4).

### Macrofossils

The fossiliferous outcrops were saturated with water and often overgrown, making the standard bench-quarrying techniques and enormous sample sizes of dryland paleobotany impossible. Quarrying generally was shallow and laterally extended to prioritize the driest or least weathered blocks, split using a rock hammer or pocket knife. All potentially identifiable macrofossils were collected and later lab-tallied (Table 1; Appendix 2) to ensure an unbiased sample, a first, to our knowledge, for a Cenozoic paleobotanical collection in the Malesian region. Unidentifiable material often appeared as hash or other tiny fragments (“Un” in Appendix 2).

**Table 1:** Summary of paleobotanical collections.

Species or morphotype	Organ	Berakas Beach	Kampong Lugu	Total
*Dipterocarpus* sp. BR01 (Dipterocarpaceae)	L	7	8	15
*Dipterocarpus* sp. BR02 (Dipterocarpaceae)	L	0	5	5
*Dryobalanops* sp. BR03 (Dipterocarpaceae)	L	1	87	88
*Shorea* sp. BR04 (Dipterocarpaceae)	F	5	0	5
cf. Malvaceae sp. BR05	L	1	0	1
Melastomataceae sp. BR06	L	2	2	4
cf. Myrtaceae sp. BR07	L	1	0	1
cf. Myrtaceae sp. BR08	L	2	0	2
*Ziziphus* sp. BR09 (Rhamnaceae)	L	2	0	2
Dicot sp. BR10, probable dipterocarp	L	3	0	3
Dicot sp. BR11	L	1	0	1
Dicot sp. BR12	L	0	1	1
Dicot sp. BR13, similar to *Shorea*	L	0	2	2
Dicot sp. BR14	L	1	0	1
Dicot sp. BR15	L	1	0	1
Dicot sp. BR16	L	0	1	1
Dicot sp. BR17	L	1	0	1
Dicot sp. BR18, probable dipterocarp	L	0	1	1
Dicot sp. BR19	L	0	1	1
Dicot sp. BR20	L	0	1	1
Dicot sp. BR21	L	1	0	1
*Rhaphidophora* sp. BR22 (Araceae)	L	1	0	1
cf. Arecaceae sp. BR23	L	0	1	1
Monocot sp. BR24	L	0	1	1
Unknown sp. BR25	F?	1	0	1
**Totals**				
Identified to species or morphotype	n/a	31	111	142
Fossiliferous slabs collected	n/a	136	203	339
Slabs without identifiable material	n/a	105 (77%)	98 (48%)	203 (60%)
Morphotypes	n/a	16	12	25
Unique morphotypes at locality	n/a	13	9	n/a

**Note:**

Specimen totals by species are fossil counts, not slab counts, and slightly exceed slab counts because of co-occurrences (Appendix 2). Berakas Beach, localities PW1501 and PW1502; Kampong Lugu, locality PW1503 (Figs. 1–3). L, leaves/leaflets. F, fruits.

Macrofossils were trimmed, usually with a pocket knife due to the soft, wet matrix, provided a unique field number (Appendix 2) with letter suffices indicating parts and counterparts, and field-photographed (when conditions permitted) to create an immediate visual record. The total macrofossil collection consists of 339 compression specimens (slabs, some containing multiple fossils; Appendix 2), 136 from Berakas Beach and 203 from Kampong Lugu (Table 1). Each specimen was field-wrapped in plastic film to slow drying and thus avoid catastrophic cracking, then in sanitary paper to increase protection and wick away moisture. The specimens were packed into suitcases for shipping, and these were stored in air-conditioned rooms at Universiti Brunei Darussalam to dry for several months. The repository of the fossils is the Herbarium of Universiti Brunei Darussalam (UBDH).

The specimens were removed from suitcases and inspected on receipt of the loaned material at the Penn State Paleobotany Laboratory (loan export approved by Muzium Brunei 19 September 2015, reference JMB/654/86/17). Although there was mold growth on the wrapping paper, and some fragile specimens had minor breakage, nearly all fossil material was undamaged. Moldy material was removed and the collection left, still wrapped, for about two more months to ensure complete drying. The dried fossils were more friable and considerably lighter in color since the time of collection, but they were undamaged and stable for the most part. We completely unwrapped them and labeled the slabs with their field numbers (Appendix 2), using a Brady Lab Pal handheld label printer (Brady, Milwaukee, WI, USA). *In-situ* cuticle was rare and unusably degraded, although dispersed cuticle found in the palynological analyses holds potential for future study (see Results). Following vetting of the collection, a UBDH collection number was assigned to each field-numbered slab (Appendix 2). Both UBDH numbers and field numbers (Appendix 2) are referenced in our descriptions for faster correlation to field data, specimen labels, and photographs.

Specimens were prepared and cleaned with standard air tools (Paleotools Micro Jack 2 and PaleoAro; Paleotools, Brigham, UT, USA) and precut Paleotools needles mounted on pin vises. The collection was photographed with Nikon D90 and D850 DSLR cameras under polarized light (Nikon USA, Melville, NY, USA). The specimens were usually lit only from one side of the copy stand to increase surface contrast and relief capture. Reflected-light and epifluorescence microscopy were done on a Nikon SMZ-1500 stereoscope with a DS-Ri1 camera and Nikon NIS Elements v. 2 and 3 software. However, due to the generally limited preservation of detail in the fossils and low contrast with the matrix, DSLR photography almost always showed fine features better than microscope photography. Many specimens were photographed at multiple focal points to increase detail capture from uneven fossil surfaces, and the series of highest interest were then *z*-stacked in Adobe Photoshop CC (Align and Blend functions; Adobe, San Jose, CA, USA) to increase the depth of field. A few photos were laterally merged (stitched) from overlapping panels, using the Photomerge macro in the same application.

The resulting image library was organized in parallel with the physical specimens using the Adobe Bridge CC visual browser, using the keyword functions to develop a set of searchable and filterable metadata attributes for each fossil (Wilf et al., 2017). Standard leaf architectural terms (Ellis et al., 2009) were applied hierarchically as Bridge keywords (characters) and sub-keywords (character states). This simple, intuitive system allows rapid filtering and visual comparisons using the Bridge filter and search functions and a smooth workflow from Bridge to Adobe Camera Raw and Photoshop. Camera Raw was used for reversible whole-image adjustments of crop, alignment, contrast, grey levels, and color temperature to increase the visibility of features, then to export the images to Photoshop as smart layers with a standard sRGB color profile. Photoshop was used to compose the macrofossil plates at 1,200 dpi, using the Smart Layers feature for continued reversible editing of the layers in Camera Raw (launched directly from the Photoshop layers palette) and maintenance of full image resolution. One dipterocarp fruit fossil was selected for CT scanning to ascertain if any taxonomically informative features were hidden in the sediment. Scanning was done by Whitney Yetter and Timothy Stecko at the Penn State Center for Quantitative Imaging, using a General Electric v|tome|x L300 system at 20 μm resolution (see https://iee.psu.edu/labs/center-quantitative-imaging/general-electric-micro-nano-ct-system). Scan data were post-processed using ImageJ Fiji (open access at https://imagej.net/software/fiji/downloads) and Avizo (Thermo Fisher Scientific, Hillsboro, OR, USA) software.

The complete image library of the Brunei macrofossil collection is available at full resolution on Figshare Plus, DOI 10.25452/figshare.plus.16510584, providing open access to far more material than we can illustrate in this article. The image library includes all field and lab images of the macrofossils, converted only once to jpeg format with minimal compression from camera raw or tiff format; high-resolution (1,200 dpi) versions of the composed macrofossil plates; CT reconstruction animations; and an archive of the CT raw image stacks and acquisition data.

The leaf architecture data generated for each specimen were applied to segregate the material into distinctive morphotypes, each with a two-letter prefix (here, “BR”; Table 1; Appendix 2) and a designated exemplar specimen, as long practiced in angiosperm leaf paleobotany (*e.g*., Johnson et al., 1989; Ash et al., 1999; Iglesias et al., 2021). The morphotype system is informal by nature and widely used as a prelude to formal systematic work, with the exemplar specimens as potential future type specimens. We organized the morphotypes systematically to the degree possible and described them informally (see Morphotype Descriptions). We use “species,” “morphotypes,” and the “BR” morphotype codes interchangeably in the text for convenience and readability. The nature of the material limited the number of recognizable entities, and we assume that the number of species that contributed to the assemblage was much higher than the number of recognized morphotypes. We only assigned morphotypes based on distinctive preserved characters, and the majority of the specimens were unidentifiable (Table 1). Several morphotypes probably represent multiple biological species with similar features. Most leaves had similar physiognomy typical of tropical rainforest assemblages, such as elliptic and untoothed blades.

No new nomenclature or type specimens are declared at this time due to the nature of the work, which intends to survey the whole flora so far collected and to lay a foundation for future paleobotanical research in a new area; additional specimens are likely to increase understanding of the fossil taxa and support formal treatments. In addition, many of the fossils could represent extant species or do not preserve diagnostic characters that differentiate them from living taxa. For readability, botanical authorities are listed only in the Morphotype Descriptions section. Nomenclature and authorities follow World Flora Online (http://www.worldfloraonline.org). The conservation status for various taxa discussed comes from the IUCN Red List (IUCN, 2021), Bartholomew et al. (2021), and other sources as cited.

Reference material included a variety of physical and digital herbarium specimens and cleared leaves, in addition to the literature cited. Digital resources that were especially useful, among many, for comparative material from the region included the websites for the Naturalis Biodiversity Center, Leiden (L, https://bioportal.naturalis.nl), the Herbarium of Universiti Brunei Darussalam (UBDH, http://ubdherbarium.fos.ubd.edu.bn), Muséum National d’Histoire Naturelle, Paris (P, https://science.mnhn.fr/institution/mnhn/collection/p//list), the Herbarium of the Arnold Arboretum, Harvard University Herbaria (A, https://huh.harvard.edu), and JStor Global Plants (https://plants.jstor.org). For cleared leaves, we consulted slides and images from the National Cleared Leaf Collection (NCLC) held in the Division of Paleobotany of the National Museum of Natural History, Washington, D.C., including both the Jack A. Wolfe (NCLC-W) and Leo J. Hickey (NCLC-H) contributions. These collections are also online at http://clearedleavesdb.org (NCLC-W) and https://collections.peabody.yale.edu/pb/nclc (NCLC-H) and were recently consolidated digitally as part of an open access leaf-image dataset (Wilf et al., 2021).

The order of morphotype listing is 22 “dicots” (non-monocot angiosperms), three monocots, then one morphotype with unknown affinities. Length and width measurements are estimated if 1 cm or less of the missing dimension was inferred by eye. If this was not possible, or if the largest dimension was found in a fragmented specimen, the largest measurable dimension for the species is given as a minimum length or width denoted as an inequality (*i.e*., “length > 5 cm”). Insect-feeding damage was recorded (Appendix 2) following the damage type (DT) system of Labandeira et al. (2007) and is included in the morphotype descriptions.

## Results

### Palynoflora

Palynological results are summarized for the Berakas Beach and Kampong Lugu sites in Appendix 1, Table 2, and Fig. 4. Fern spores and fungal bodies dominate both assemblages. Mangrove pollen is common at Kampong Lugu but comparatively rare at Berakas Beach. Uncommon tree and liana pollen at both sites record deposition primarily from the adjacent lowland rainforests, which were the sources of the abundant leaves that were transported into the depocenters and fossilized, and to some extent from more distant slope forests. Dipterocarp pollen was notably rare, even though the family dominated all the leaf assemblages (see Macroflora). Additional microfossil elements include freshwater and marine algae, dinocysts, and foraminifera.

**Table 2:** Biostratigraphically significant palynomorphs at Berakas Beach (BE) and Kampong Lugu (KL).

Taxon	Occurrence	Range
*Crassoretitriletes vanraadshooveni*	KL	Late Eocene–Recent, Southeast Asia
*Florschuetzia levipoli*	KL	Early Miocene–Pleistocene, Southeast Asia
*Perfotricolpites digitatus*	BE, KL	Late Eocene–Pleistocene, Borneo
*Praedapollis* spp.	BE	Late Eocene–Pliocene, Africa
*Rostriapollenites robustus*	BE, KL	Middle Eocene–Recent, Papua New Guinea
*Scolocyamus magnus*	BE	Earliest late Miocene–early Pliocene, Southeast Asia
*Stenochlaenidites papuanus*	BE	Late Miocene–Pliocene, Southeast Asia

#### Age constraints

Age-specific palynomorphs are rare in the assemblages (Table 2; Appendix 1). At Berakas Beach, the co-occurrence of *Stenochlaenidites papuanus*, *Perfotricolpites digitatus*, *Scolocyamus magnus*, and *Rostriapollenites robustus* indicates an age from late Miocene to early Pliocene. At Kampong Lugu, the co-occurrence of *Crassoretitriletes vanraadshoveni*, *Florschuetzia levipoli*, *Perfotricolpites digitatus*, and *Rostriapollenites robustus* suggests an age range from early Miocene to Pleistocene. Considering these results along with the scarce geological age constraints (see Geological setting), the Berakas Beach fossils are probably early Pliocene but could be as old as the latest Miocene, and those from Kampong Lugu are most likely younger than Berakas Beach and Pliocene or Pleistocene in age.

#### Berakas Beach

A very high abundance assemblage of well-preserved palynomorphs was recorded from both Berakas Beach samples, which are very similar to each other (Appendix 1). The assemblage is dominated by terrestrially derived miospores and fungal bodies and includes rare marine microplankton and freshwater algae. Marine microplankton (<1% of the total sample) includes the marine alga *Tasmanites* sp., the dinocysts *Operculodinium* sp. and *Spiniferites* sp., and a foraminiferal test lining. The freshwater alga *Botryococcus* sp. (typical of still-standing water) is very common. The *Botryococcus* specimens are structureless or almost structureless and occur as fragments. This type of preservation suggests stressed environmental conditions in the depocenter, possibly brackish and with a short growth period (Guy-Ohlson, 1992).

The miospore assemblage is dominated by fern spores (Appendix 1), including abundant specimens of *Laevigatosporites* (parent plant ?*Thelypteris*) and common specimens of *Cyathidites* (?*Pteridium*) and *Verrucatosporites* (*Polypodium* and *Stenochlaena*). Rare specimens indicate a higher diversity and ecological range of ferns, including grains associated with *Blechnum* and *Stenochlaena* (Blechnaceae), *Ctenitis* (Dryopteridaceae), *Osmunda* (Osmundaceae), and *Ceratopteris* and *Pteris* (Pteridaceae). Rare but diverse specimens of lycophyte spores correspond to *Isoetes* (Isoetaceae), *Huperzia*, *Lycopodiella*, and *Lycopodium* (Lycopodiaceae), and *Selaginella* (Selaginellaceae).

Mangrove pollen at Berakas Beach includes rare specimens of *Avicennia*, *Rhizophora*, and the mangrove palm *Nypa*. Tropical rainforest pollen include low numbers of Euphorbiaceae, Malvaceae, Ctenolophonaceae, Dilleniaceae, Dipterocarpaceae, ?Fabaceae, Lecythidaceae (*Barringtonia*), Myristicaceae, Rubiaceae (*Lasianthus*), Rutaceae, and Sapotaceae. Likely lianas and parasites include grains affiliated with *Calamus* (rattan palm, Arecaceae), Loranthaceae, and *Merremia* (Convolvulaceae). Specimens of potentially upland-derived *Podocarpus* (Podocarpaceae) and *Ilex* (Aquifoliaceae) are present in low numbers, although both also occur in lowlands today.

Fungal bodies occur at very high abundance. These include very common fungal hyphae, abundant and diverse fungal spores (mainly *Inapertisporites* and *Mediaverrunites*, with *Alleppeysporonites*, *Brachysporosporites*, *Dicellaesporites*, *Diporisporites*, *Dyadosporites*, *Fusiformisporites*, *Multicellaesporites*, *Multicellites*, and *Pluricellaesporites*), and common fungal fruiting bodies (mainly *Phragmothyrites*). Kerogenaceous material is predominantly of terrestrial origin, including abundant plant cuticle, common degraded vitrinite, relatively common structured inertinite and structured dark vitrinite, and rare plant tracheids. The cuticle occurs as fragments of ca. 50 to > 300 micron size, some showing good cell structure and stomata with potential for future study through a dedicated maceration effort. Reworked or transported material includes one specimen each of the bisaccate gymnosperm pollen *Pityosporites* from Mesozoic or older sediments, *Spinizonocostites echinatus* (Maastrichtian–Eocene), and *Cicatricosisporites* (Cretaceous or younger), as well as taxodioid conifer pollen presumably transported from mainland Asia.

Palynotaxa from Berakas Beach reported (from a higher stratigraphic level; see Fig. 2) by Roslim et al. (2021) without likely counterparts in our samples included representatives of Lythraceae (*Florschuetzia levipoli*, equivalent to the mangrove *Sonneratia* and present in this study at Kampong Lugu), Anacardiaceae, Annonaceae, and coryphoid Arecaceae (*Borassus*). Roslim et al. (2021) did not report any dipterocarp pollen from the site, and the present study indicates a wider variety of angiosperms and ferns.

Overall, the Berakas Beach palynological assemblage is dominated by ferns and marsh- or swamp-derived fungi, along with freshwater algae, rare mangrove pollen, and low numbers of lowland forest and slope-forest pollen. The data indicate that the depositional environment was a lowland, fern-dominated swamp with some restricted marine influence, into which occasional palynomorphs (and abundant leaves) were deposited from the adjacent lowland tropical rainforest, with additional pollen input from more distant areas.

#### Kampong Lugu

At Kampong Lugu, a very high abundance assemblage of well-preserved palynomorphs was found in both samples, which are self-similar (Appendix 1). Terrestrially derived miospores and fungal bodies dominate this assemblage, which also includes rare marine microplankton such as dinocysts (*Impletosphaeridium*, *Operculodinium*, and *Spiniferites*), the marine algae *Leiosphaeridia* and *Tasmanites*, and two foraminiferal test linings. Freshwater algae include common to very common *Chomotriletes* and abundant *Botryococcus* (typical of still-standing water). The specimens of *Botryococcus*, as at Berakas Beach, are structureless or almost so, a type of preservation suggesting stressed, possibly brackish environmental conditions (Guy-Ohlson, 1992).

The Kampong Lugu miospore assemblage is dominated by fern spores, including abundant specimens of *Laevigatosporites* and *Cyathidites*; common *Verrucatosporites* (including *V. favus* and *V. usmensis*); and diverse but rare spores affiliated with the mangrove fern *Acrostichum* (Pteridaceae), *Asplenium* (Aspleniaceae), *Blechnum* and *Stenochlaena* (Blechnaceae), *Ctenitis* (Dryopteridaceae), Gleicheniaceae, *Hymenophyllum* (Hymenophyllaceae), *Lygodium* (Lygodiaceae), *Osmunda* (Osmundaceae), *Drynaria* (Polypodiaceae), and *Pteris* (Pteridaceae). These taxa indicate a wide range of pteridophyte life habits, from ground cover and potential tree ferns to likely climbers (*Lygodium*, *Stenochlaena*) and epiphytes (*Hymenophyllum*, *Asplenium, Drynaria*). Lycophytes include rare spores affiliated with *Huperzia* and *Lycopodium*.

Mangrove pollen include common to very common *Zonocostites ramonae* (*Rhizophora*) and rare *Avicennia*, *Florschuetzia levipoli* (linked to *Sonneratia*, Lythraceae; see Roslim et al., 2021), and *Nypa*. Tropical rainforest pollen is rare but represents diverse taxa, including Combretaceae, Convolvulaceae (*Merremia*), Dipterocarpaceae, Euphorbiaceae, ?Fabaceae, Lecythidaceae (*Barringtonia*), Malvaceae, Myristicaceae, Polygonaceae, Rubiaceae (*Lasianthus*), and Sapotaceae. Potential upland-derived pollen includes *Myrica*, *Ilex*, and *Podocarpus*, with the caveats previously stated.

Fungal bodies were found at very high abundance, dominated by hyphae. Common fungal spores mainly comprise *Brachysporisporonites*, *Inapertisporites*, and *Mediaverrunites*. Fungal fruiting bodies (mainly *Phragmothyrites*) were also abundant. *Cirrenalia pygmea*, found at low abundance, is associated with decaying submerged, intertidal wood, especially mangrove wood (Abdel-Wahab et al., 2010). Kerogenaceous material was predominantly terrestrially derived, mainly including abundant plant cuticle (like Berakas Beach), with relatively common structured and unstructured inertinite, structured vitrinite, and rare plant tracheids.

In summary, the Kampong Lugu palynological assemblage is dominated by ferns and mangroves, with marsh-swamp-derived fungi, freshwater algae, and rare but diverse additions from the nearby tropical lowland rainforest and potentially more distant hill forest. The sample indicates that the depositional environment was a mangrove swamp with a restricted marine influence, into which occasional palynomorphs were deposited from the nearby lowland tropical rainforest (also the source of the abundant leaves) and more distant hill forests.

### Macroflora

The macrofloral collection from Berakas Beach and Kampung Lugu, combined, totaled 339 fossiliferous slabs, each of them containing one or more fossil leaves or other plant specimens, of which 136 slabs (40%) preserved material of 142 individual plant fossils that we sorted into 25 distinct morphotypes (Table 1; Appendix 2). Fossils on the remaining 60% of slabs lacked preservation of distinctive features and were categorized as unidentifiable (“Un”; Appendix 2). Preservation was significantly better at Kampong Lugu, where a majority of slabs had identifiable material, compared with less than a fourth at Berakas Beach (Table 1). However, despite the much smaller sample size of identifiable material at Berakas Beach, morphotype diversity was a third higher, including more unique morphotypes than Kampong Lugu and the only reproductive material in the collections (Table 1).

Nearly all specimens identified to morphotype are “dicot” (non-monocot angiosperm) leaves, with rare reproductive structures and monocot leaf fragments. Even though the total sample size was limited by preservation, it is clear (Table 1) that dipterocarp remains overwhelmingly dominate both assemblages by relative abundance, comprising 79% of total identified specimens (42% at Berakas Beach and 90% at Kampung Lugu); the four dipterocarp morphotypes also occupy the four highest-ranked abundances overall. Dipterocarps also rank highest in observed diversity, with evidence for at least four species in three genera (*Dipterocarpus*, *Dryobalanops*, and *Shorea*). *Dryobalanops* leaves were, by far, the most abundant taxon in the whole collection, especially at Kampong Lugu, where they comprised 78% of specimens (Table 1). Several other morphotypes (Table 1) and many unidentified leaves also appear to represent dipterocarps, although we did not assign them to the group. Other groups present include Melastomataceae, Rhamnaceae (*Ziziphus*), Araceae (*Rhaphidophora*), and probable Malvaceae, Myrtaceae, and Arecaceae. Notably, the dipterocarp-rich macrofloras lack a single fern fossil and are strikingly different from the fern-dominated, dipterocarp-poor palynofloras derived from the same strata, reflecting significant differences in preservational filters and pathways. Several factors inhibit the preservation of tropical fern macrofossils, including low biomass, lack of dehiscence, and the low potential of epiphytic fern remains to reach depocenters before they decompose (Scheihing & Pfefferkorn, 1984; see Introduction regarding dipterocarp pollen preservation). Insect damage was rare (Appendix 2), presumably because of overall preservation quality, and included several types of external feeding (hole feeding and skeletonization) as well as possible galls and a possible mine (Appendix 2). No domatia were observed on fossil dipterocarp leaves (Guérin, 1906), probably due to preservation limitations.

***Morphotype Descriptions***

Family Dipterocarpaceae Blume

Genus *Dipterocarpus* C.F. Gaertn.

**Figure 5:**
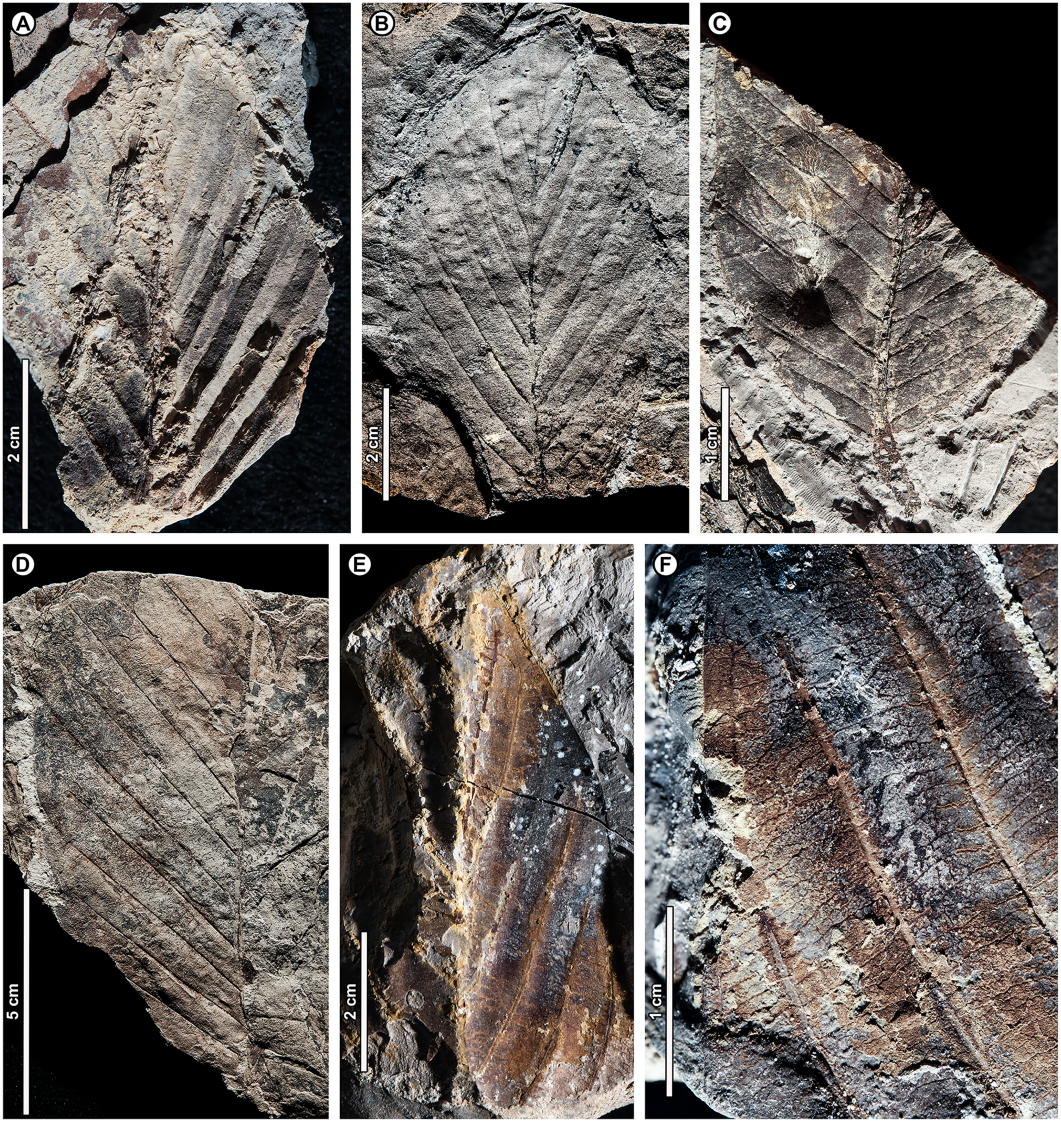
*Dipterocarpus* sp. BR01. (A) UBDH F00253b (Kampong Lugu), with well-preserved plications; (B) UBDH F00090a (Berakas Beach), with subtle preservation of plications; (C) UBDH F00156 (Kampong Lugu), preserving base and stout petiole; (D) UBDH F00096 (Berakas Beach), large leaf fragment with good vein preservation; (E) UBDH F00140b (Kampong Lugu), under low-angle unidirectional light to show the stout midvein and strong longitudinal folding of the blade. (F) UBDH F00140a, counterpart of specimen in E, preserving margin and venation details.

*Dipterocarpus* sp. BR01 (Fig. 5)

*Exemplar specimen*. UBDH F00090a,b (field number PW1501-90a,b, from Berakas Beach; Fig. 5B).

*Additional material*. Six specimens from Berakas Beach and eight from Kampong Lugu, as illustrated in Fig. 5 and tabulated in Appendix 2.

*Distinguishing features*. Morphotype BR01 (= *Dipterocarpus* sp. BR01) has a thick midvein with strong relief, a longitudinally well-folded blade, and plicate vernation, each of these features making marked impressions in the sediment (Fig. 5). Depending on the amount of compression, the plications preserve with a strongly corrugated texture (Fig. 5A) or, more commonly, as subtle to pronounced longitudinal bulges in the intercostal areas (Figs. 5B–5F). The petiole is stout (Fig. 5C). Secondary veins are robust, up to at least 11 pairs, and regular, course eucamptodromous and unbranched, angle gradually becoming more acute apically. Tertiaries are numerous, thin, and opposite percurrent (Fig. 5F). The margin is entire and not visibly sinuate.

*Description*. Petiole stout, length > 7.4 mm, width 2.4 mm (*n* = 1); blade attachment marginal; blade plicate and strongly folded longitudinally. Lamina length to > 12.9 cm; width 4.1–9.1 cm (*n* = 8); length:width (L:W) ratio 1.9:1 (*n* = 3). Base symmetrical. Margin unlobed and entire, not sinuous or crenate. Base angle acute; base shape convex. Apex angle acute. Primary venation pinnate with thick, raised midvein; agrophic veins absent. Major secondaries robust, up to at least 11 pairs, eucamptodromous, straight to gently curved approaching margin, not branching, spacing basally crowded, angle to the midvein smoothly decreasing and becoming much more acute apically. Intercostal tertiary veins thin, dense, straight opposite percurrent, obtuse to midvein; tertiary vein angle consistent. Epimedial tertiaries opposite percurrent; proximal course obtuse to midvein; distal course parallel to the intercostal tertiaries. Quaternary and quinternary vein fabric regular reticulate. Hole-feeding damage is present (DT2).

*Remarks*. Features of the fossils commonly found in Dipterocarpaceae (Ashton, 1964, 1982) include their elliptic blades with thick, high-relief midveins, prominent longitudinal folding, and thickened petioles; regular, unbranched secondaries; and dense, opposite percurrent tertiary veins. Combined with the well-preserved plications, these features place the fossils with high confidence in Dipterocarpaceae. The two living dipterocarp genera that often have plicate vernation are *Dipterocarpus* and *Parashorea* Kurz, which nests within *Shorea* Roxb. ex C.F. Gaertn. in molecular phylogenetic analyses (Heckenhauer et al., 2017; Ashton et al., 2021). Ashton (1982) described *Parashorea* species as having unthickened or barely thickened petioles, unlike the prominently swollen petioles in *Dipterocarpus* and the fossils. Although the fossils do not have sinuate margins, many living species of *Dipterocarpus* also lack this feature. From all the evidence, we consider the fossils to represent one or perhaps more species of *Dipterocarpus*. Specimens from Berakas Beach and Kampong Lugu have no discernible differences, although they may well have originated from different species with similar leaf morphology. See Remarks for morphotypes BR02 (*Dipterocarpus* sp. BR02), BR13, and BR18 for additional comparisons within the fossil assemblage.

*Dipterocarpus* (Keruing) is a widespread genus of medium to large trees, with ca. 70 species from Sri Lanka through India and Indochina and the Malay Archipelago to the Philippines; in Brunei, there are ca. 26 species, most occurring below ca. 900 m altitude, especially in habitats with high insolation such as riparian corridors, heath forests, and ridges (Ashton, 1964; Ashton, 2004; Coode et al., 1996). Borneo is the center of diversity and endemism for the genus (Ashton, 1982).

**Figure 6:**
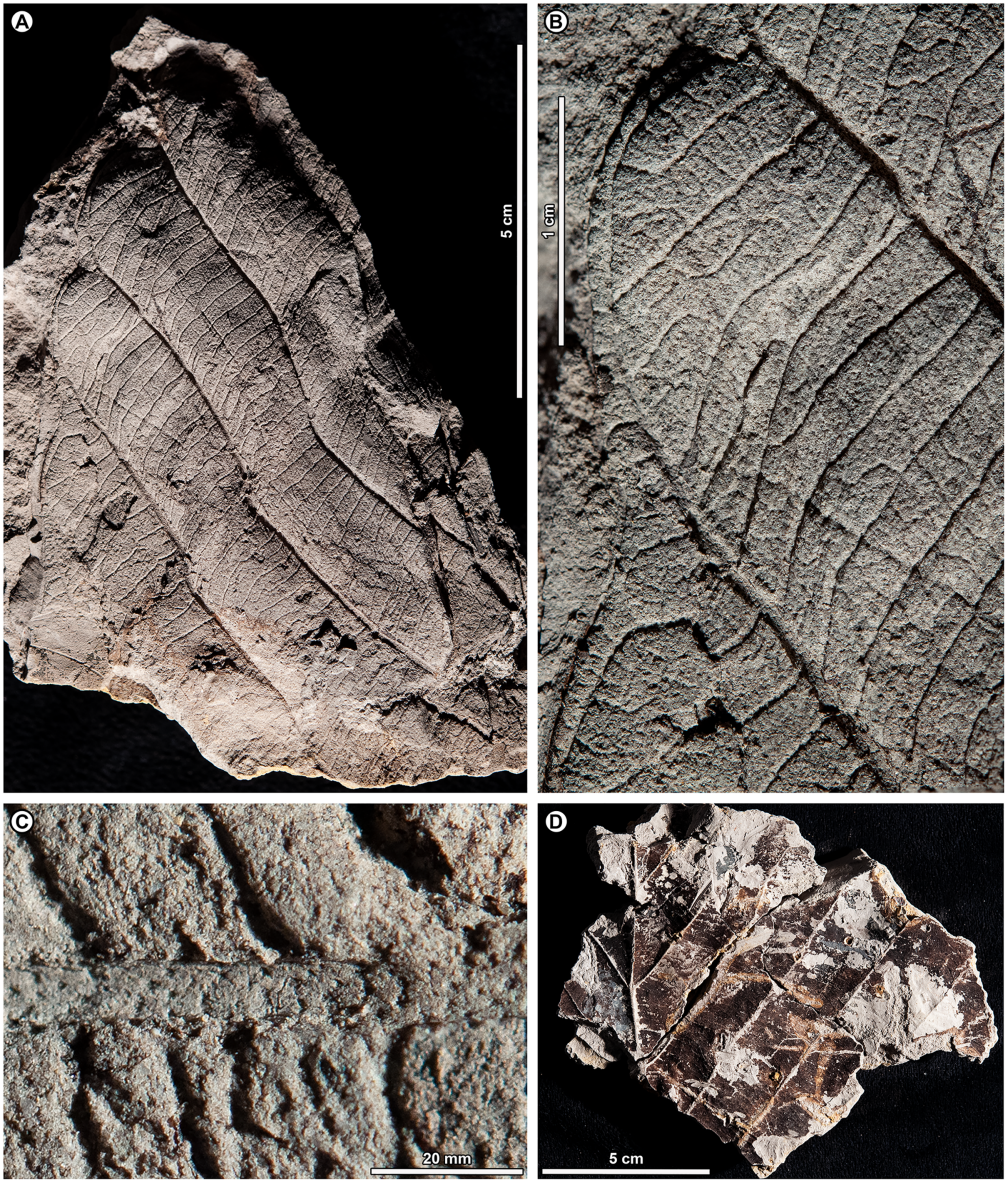
*Dipterocarpus* sp. BR02. All from Kampong Lugu. (A–C) UBDH F00137b, large fragment with well-preserved venation, plications, marginal sinuations, and numerous hair bases preserved on secondary veins and lamina (detailed in C); (D) UBDH F00150, large fragment preserving the midvein and widely spaced secondary veins.

*Dipterocarpus* sp. BR02 (Fig. 6)

*Exemplar specimen*. UBDH F00137a,b (PW1503-1a,b, from Kampong Lugu; Figs. 6A–6C).

*Additional material*. Four specimens from Kampong Lugu (Fig. 6D; Appendix 2).

*Distinguishing features*. Morphotype BR02 has very large leaves, along with brochidodromous, widely spaced major secondaries and a prominently sinuous (per Ashton, 1964) margin following the secondary loops, combined with plications preserved as longitudinal furrows in the intercostal areas (Figs. 6A, 6B). Tertiary veins are strongly opposite percurrent with convex course, prominent, and regular (Fig. 6B). The lamina and secondary veins (and not the adjacent sediment) are covered with minute pits inferred to be hair-base impressions (Fig. 6C).

*Description*. Midvein stout with high relief, blade preserving plications; length not measurable; width on one specimen inferred as >> 22 cm, the largest in the collection (Fig. 6D); margin strongly sinuous following secondary loops. Primary venation pinnate; major secondaries simple brochidodromous with strong loops closely aligned with the marginal sinuations, spacing regular, wide (to ca. 2 cm), angle smoothly decreasing apically. Intercostal tertiary veins prominent, opposite percurrent, course convex, obtuse to midvein; vein angle consistent; epimedial tertiary veins perpendicular to midvein at departure, then parallel to intercostal tertiaries. Quaternary veins mixed percurrent, quinternary veins regular reticulate. Base not preserved, apex poorly preserved. Hair-base pits ubiquitous on the major veins and laminar surface of the presumably once-tomentose blade.

*Remarks*. The combination of a plicate, tomentose blade, a prominently sinuous margin that follows strong brochidodromous secondary loops, a thick and raised midvein, regular stout secondaries, and regular opposite percurrent tertiaries clearly points to affinity with *Dipterocarpus*, even in the absence of a preserved leaf base. Some specimens have very widely spaced secondary veins, indicating notably large leaf sizes based on scaling relationships (Sack et al., 2012). Even as a fragment, the exemplar specimen is already one of the largest fossils in the collection (Fig. 6A), and we estimate its original leaf area at ca. 16,000 mm^2^ (large mesophyll) based on the vein-scaling method of Sack et al. (2012). Among living *Dipterocarpus* species in Brunei, the preserved features of the fossil, including size, brochidodromy, prominent percurrent tertiaries, and conspicuous marginal sinuations, are most similar to three species noted by Ashton (1964) for their very large leaves: *D. confertus* Slooten, *D. elongatus* Korth (*D. apterus* Foxw.), and *D. humeratus* Slooten. The marginal curvature and strong sinuations observed in the fragmentary fossils seem incompatible with the elongate, comparatively straight-sided leaves with relatively shallow sinuations of *D. elongatus*. However, the fossils are very similar in their preserved architecture to the other two species listed. Of those, *D. confertus* has a more conspicuously hairy leaf surface, especially on the veins, corresponding to the numerous hair bases preserved in the fossils (Fig. 6C). *Dipterocarpus confertus* is a Near Threatened, Borneo endemic species to 50 m tall, occurring in mixed dipterocarp forests below 800 m (Ashton, 1964; Ashton, 1982; Ashton, 2004).

Morphotypes BR01 and BR02, both assigned to *Dipterocarpus*, are distinguished based on the non-sinuate margin and mostly eucamptodromous secondaries in BR01, compared with the strongly sinuate margin, brochidodromous secondaries, and numerous surficial hair-base pits in BR02. The widely spaced secondaries and very large leaf size in some BR02 specimens further distinguish it from BR01.

Genus *Dryobalanops* C.F. Gaertn.

**Figure 7:**
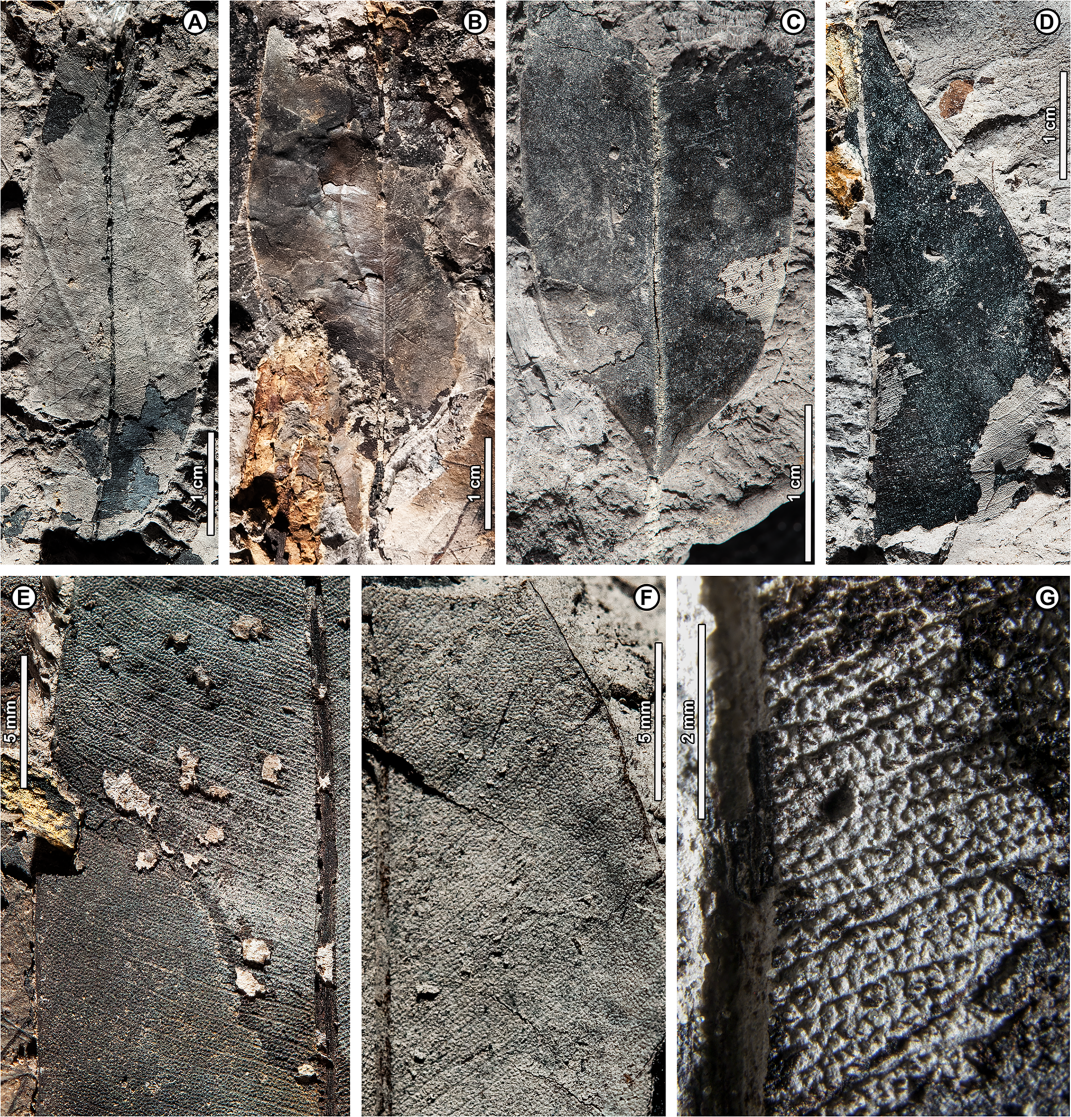
*Dryobalanops* sp. BR03. All shown are from Kampong Lugu. (A) UBDH F00332, relatively complete specimen preserving overall leaf form and venation; (B) UBDH F00151, preserving upper petiole, base, strong longitudinal folding along midvein, and general venation; (C) UBDH F00292, preserving upper petiole, intramarginal vein emerging near the base, and longitudinal folding; (D) UBDH F00183 (also G), preserving part of a broad-acuminate apex and showing bright patches of well-preserved venation where the coalified surface has flaked off; (E) UBDH F00192a, leaf portion with well-preserved venation, including intersecondary veins, rectangular tertiary-vein fields, and intramarginal vein running very close to the margin; (F) UBDH F00323, leaf portion with preservation similar to E; (G) Detail of venation exposed along the midvein in D, showing sediment pushing through the rectangular tertiary-vein fields.

*Dryobalanops* sp. BR03 (Fig. 7)

*Exemplar specimen*. UBDH F00032a,b (PW1501-32a,b, from Kampong Lugu; Fig. 7A).

*Additional material*. One specimen from Berakas Beach and 86 from Kampong Lugu (Fig. 7; Appendix 2).

*Distinguishing features*. Morphotype BR03 is an elliptic to ovate-lanceolate microphyll with a straight (cuneate) base, prominent midvein with high relief (Figs. 7B, 7C), and broad-acuminate apex (Fig. 7D). The blade is longitudinally folded (Figs. 7B, 7C), making a strong impression in the sediment along with the midvein. Major secondaries (Figs. 7E–7G) are numerous, very thin, unbranched, high-angled, and closely spaced, entering an intramarginal vein that originates near the base and runs barely inside the margin (Fig. 7C); secondaries alternate with very thin intersecondaries that are more deflected than, but with length nearly as long as, the secondaries. Tertiaries are regular reticulate, in small, well-defined rectangular or other polygonal fields, the fields packed in ca. two rows per secondary-intersecondary pair. The fine vein mesh is often pushed through with tiny sediment plugs (Fig. 7G). The blade is often coalified; cracking in the coal presents artifactual patterns resembling venation, which is best seen where the coal has flaked off or is manually removed with a needle to reveal the venation impression underneath.

*Description*. Blade attachment marginal. Petiole length > 9.3 mm, width to ca. 1.6 mm (*n* = 2); petiole not thickened at insertion and often preserved in a microstratigraphically offset position from the well-impressed, folded blade and thickened, high-relief midvein. Blade apparently coriaceous, based on the extensive coalification observed. Midvein prominent with strong relief and blade longitudinally folded, each feature impressing the sediment. Lamina length 4.0–7.7 cm (*n* = 6); width 0.7–5.0 cm (*n* = 44); L:W ratio 2.8:1 (*n* = 6); lamina shape elliptic or ovate-lanceolate, symmetrical. Margin unlobed and entire, thickened, and slightly revolute; basal margin not inrolled. Base angle acute; base shape straight with slight decurrence at insertion. Apex broad-acuminate. Primary venation pinnate. Major secondaries parallel, very thin, dense, >60 pairs, diverge from primary at a high angle, uniformly spaced, course without branching, then tightly loop barely inside the margin to join a slightly irregular intramarginal vein that arises near the leaf base and is difficult to discern from the margin. Intersecondaries parallel to major secondaries, nearly as long as the secondaries then reticulating toward the margin, course deflected by tertiaries, frequency usually one per intercostal area. Tertiary venation conspicuously regular reticulate, making small, densely packed rectangular or other polygonal fields of somewhat variable size that are packed in ca. two rows between each secondary-intersecondary pair. Quaternary and quinternary veins indistinct, apparently reticulate. Sediment plugs often push through and slightly distort the appearance of the vein mesh. Elongate slot feeding was observed, oriented parallel to secondary veins (DT8).

*Remarks*. Morphotype BR03 is by far the most common form at Kampong Lugu and in the whole collection (Table 1), also occurring as a single specimen at Berakas Beach. The distinctive venation pattern makes the morphotype easily recognizable, even from small fragments. At Kampong Lugu, there are numerous fragments of the morphotype in the sediment, attesting to even higher dominance than we could reliably tabulate. Characters of living *Dryobalanops* species (Ashton, 1964) match some to all the features of the fossils, including microphyll size, elliptic to lanceolate shape, prominent midveins, folded blades, broad-acuminate apices, dense and parallel major secondaries alternating with intersecondaries, intramarginal veins almost on the margin, and tertiary veins in small, regular fields. Although most *Dryobalanops* species are described as not having intersecondary veins, which may be obscure in fresh or herbarium material, the intersecondaries are noted in older literature (van Slooten, 1932) and are easily visible in cleared-leaf specimens. When parallel secondary venation occurs in other dipterocarps, namely *Cotylelobium* Pierre and *Hopea* Roxb., it is considerably less dense and entirely different in appearance from *Dryobalanops* (Ashton, 2004) and the fossils.

The general combination of thin, dense secondary venation and intramarginal veins occurs in several unrelated plant families (Hickey & Wolfe, 1975). Examples in the Brunei flora include Myrtaceae (*i.e*., *Syzygium* P. Browne ex Gaertn. spp.), Sapotaceae (*Payena* A. DC.), Ochnaceae (*Ouratea* Aubl.), Moraceae (*Ficus* L.), and Calophyllaceae (*Calophyllum* L.; Coode et al., 1996). However, those families and others with some comparable leaves (*e.g*., Anacardiaceae, Vochysiaceae) lack most or all key characters of the fossils, especially the high-rank reticulate tertiary mesh. For example, in Myrtaceae and Sapotaceae, the major secondaries are not nearly so densely spaced as in the fossils and living *Dryobalanops* and are less regular, the tertiaries are much less organized, and the intramarginal vein is located farther from and is easily distinguished from the margin (see also possible myrtaceous morphotypes BR07 and BR08). Other genera with species that are superficially similar to the fossils, such as *Calophyllum* and *Ouratea* spp., do not usually have a broad-acuminate apex; their tertiaries are less organized and do not form a similar mesh (a leaf fragment similar to *Calophyllum* was found at Berakas Beach: Appendix 2).

*Dryobalanops* (Kapur) is a Malesian genus of very tall to emergent (to 65 m tall) large-crowned trees with seven species; northern Borneo is the center of diversity and endemism (van Slooten, 1932; Meijer & Wood, 1964; Ashton, 1982). Four species are found in Brunei, all below ca. 800 m altitude but each in different habitats (Ashton, 1964; Coode et al., 1996): *D. aromatica* C.F. Gaertn., *D. beccarii* Dyer, *D. lanceolata* Burck, and *D. rappa* Becc. The distinctive, well-organized tertiary vein fields of the fossils are most comparable with those of *D. aromatica* (also in Sumatra, Peninsular Malaysia, and elsewhere in northern Borneo), as well as two species with ranges nearby: *D. fusca* Slooten (Sarawak and West Kalimantan) and *D. oblongifolia* Dyer (Sumatra, Kalimantan, Sarawak, and Peninsular Malaysia). Of those three species, *D. aromatica* and *D. oblongifolia* have very different leaf shapes (orbicular and oblong, respectively) from the fossils (elliptic to ovate-lanceolate), and the intramarginal vein of *D. aromatica* often arises from a pair of secondaries that diverge noticeably above the leaf base, unlike the near-basal divergence in the fossils (Fig. 7C). Overall, the fossils’ general features, including size range, petiole length, base and blade shapes, and details of the tertiary reticulation are closest to *D. fusca*, a Critically Endangered low-elevation *kerangas* species (van Slooten, 1932; Ashton, 1982; Ashton, 2004; Randi et al., 2019). However, the fossils lack or did not preserve the characteristic, dense hairs found on the lower leaf surface of *D. fusca*.

Genus *Shorea* Roxb. ex C.F. Gaertn.

**Figure 8:**
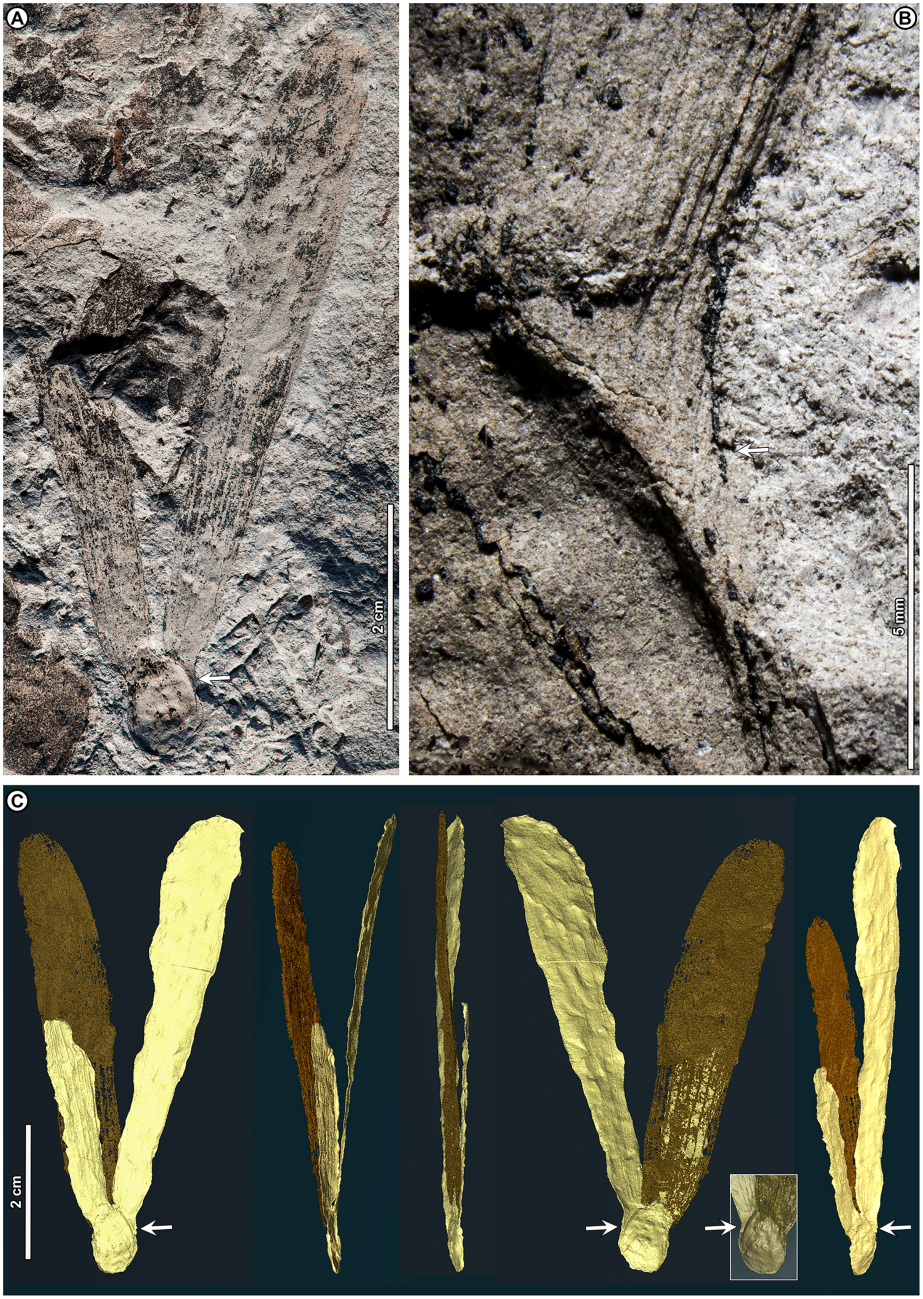
*Shorea* sp. BR04. UBDH F00097b (Berakas Beach). White arrow, shared visual reference point at a wing juncture with the nut body. (A, B) Surface view, showing an ovoid nut with two clasping, obovate, apparently subequal fruit wings (calyx lobes); the wing at left is missing its apex (fragments distal to the wing are dark-stained matrix, not fossil), but the wing at right is relatively complete; (C) Rotational views of CT scans, showing an additional large wing (dark color) embedded in the sediment directly underneath the broken wing at the surface, subequal in size and shape to the more complete wing at the surface. Inset, initial scan that captured fragments of the embedded wing (dark) clasping the obverse surface of the nut well toward the base.

**Figure 9:**
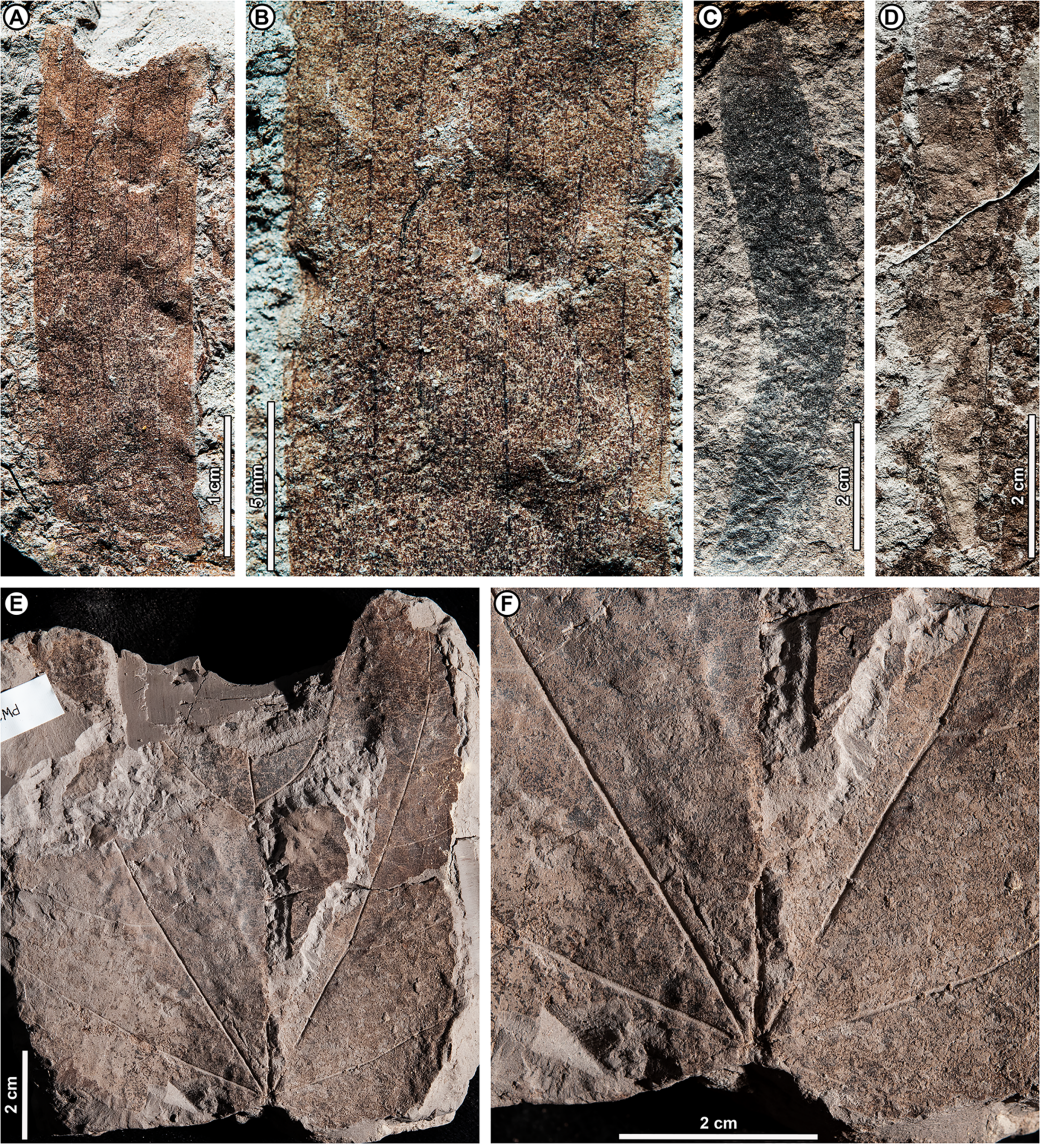
*Shorea* sp. BR04 and possible Malvaceae (sp. BR05). All from Berakas Beach. (A–D) Dispersed *Shorea* fruit-wing fragments. (A, B) UBDH F00110a, preserving parallel venation and variably angled and curved percurrent cross-veins. (C) UBDH F00022, preserving the obovate apex and part of the tapered base, coalified with weak preservation of venation. (D) UBDH F00111a, specimen with narrow wing base and poorly preserved venation. (E, F) UBDH F00136, cf. Malvaceae, with cordate base, seven basally actinodromous primary veins, well-developed agrophic veins, and concentric, opposite-percurrent interior secondary veins.

*Shorea* sp. BR04 (Figs. 8, 9A–9D)

*Exemplar specimen*. UBDH F00097a,b (PW1501-FS1a,b, from Berakas Beach; Fig. 8).

*Additional material*. Four wing fragments from Berakas Beach (Figs. 9A–9D; Appendix 2).

*Distinguishing features*. The exemplar specimen (Fig. 8) is a winged fruit consisting of a nut with an ovoid body and two attached, apparently subequal, obovate wings (calyx lobes) visible on the surface with ca. nine parallel veins each. A third wing of similar size and shape is preserved within the sediment, visible under CT scan (Fig. 8C). The wings extend basally, adpressed around the nut. The isolated wing fragments (Figs. 9A–9D) are large (to ca. 9 cm length, 1.4 cm width), obovate, with ca. 10 parallel veins, joined by percurrent, variably oriented and curved cross veins. All wing apices, when preserved, are asymmetrically rounded.

*Description*. Nut ovoid, 9.3 by 6.8 mm, making a rounded impression in the sediment, with traces of the nut wall preserved. All internal material degraded and coalified, apiculus and styles not preserved. Two attached wings (calyx lobes) of exemplar specimen visible at surface, apparently subequal, one (at left in Fig. 8A) with apical portion broken off, the other relatively complete. Wings adpressed to the nut body near the nut apex, following the remaining positive relief of the nut body basally (where still preserved) along one margin for more than 60% of the nut length, then broken off preservationally over most of the nut body (Fig. 8, white arrows). Wings obovate, length of free portion 5.8 cm, maximum width 0.9 cm at ca. 80% of the wing length, with asymmetrical, subrounded apices and ca. nine parallel veins, each arising separately from the base. Cross veins percurrent, with variable course, angle, and spacing. One wing preserved within the sediment directly underneath the broken surface wing, visible under CT (Fig. 8C), subequal to the surface wings in dimensions, shape, and venation, the base closely adpressed to the obverse face of the nut (Fig. 8C, inset).

Isolated wings obovate, preserved length to 8.7 cm, width 1.0–1.4 cm (*n* = 3), gently tapered basally and apically. Base not preserved; apex acute, subrounded, and slightly asymmetrical. Venation well preserved only in one specimen (Figs. 9A, 9B). Parallel veins ten or more; two parallel veins run close to the margin, thinner than the medial parallel veins. Cross veins percurrent, closely spaced, with angle, spacing, and course variable. Higher-order venation reticulate.

*Remarks*. The configuration of the exemplar specimen is unique to Dipterocarpaceae, including obovate, subequal, parallel-veined wings attached laterally to and clasping an ovoid nut (*e.g*., Ashton, 1982). For the dispersed wing fragments, the large size, numerous parallel veins, variable-percurrent cross venation, and presence of visually distinct higher-order venation also distinguish them as dipterocarpaceous and separate them from a suite of other extant and fossil taxa with fruit wings derived from perianth lobes, as detailed previously with regard to other dipterocarp fruit fossils (Shi & Li, 2010; Feng et al., 2013; Shi, Jacques & Li, 2014). The cross veins and lack of conspicuous resin ducts also distinguish the isolated fossils from leaves of parallel-veined gymnosperms in the region, such as *Agathis* Salisb.; the high cross-vein variability, combined with the obovate shape, are not found in any monocot leaves to our knowledge.

The exemplar specimen and the isolated wings are generally similar in having a large number of parallel veins (at least 9–10), which is typical among the dipterocarps only of *Hopea* and *Shorea* species (Shi & Li, 2010). Molecular analyses have resolved *Hopea* as a derived subclade of *Shorea* (*e.g*., Heckenhauer et al., 2017; Ashton et al., 2021). Both traditional genera have their wing bases adpressed to the nut body and no calyx tube, as in the exemplar specimen, and are usually five-winged. In most *Hopea* species, there are two extended, prominent, subequal outer calyx lobes (wings) and three reduced, non-aliform inner lobes that are mostly adpressed to the nut (and would not be preserved in these specimens). In contrast, *Shorea* most often has two small inner wings with very narrow bases and three larger outer wings with wider bases, all well extended beyond the nut body.

The exemplar specimen was found in the field with only the apex of one wing visible. After mechanical preparation, the two subequal wings and attached nut were revealed at the surface (as in Fig. 8A), appearing from all visible cues to represent *Hopea*. However, because the isolated wings from the same site (Figs. 9A–9D) appeared to represent *Shorea*, we used CT scanning to test the idea that the exemplar specimen might also represent *Shorea*, which would be the case if additional wings were present. One large wing was recovered from CT scanning (Fig. 8C), with the same size and venation as the better-preserved wing at the surface. This fortuitous discovery eliminated the possibility of *Hopea* and validated the *Shorea* hypothesis, which only requires the further, likely, presumption that the two smaller wings were lost to preservation or not detected in the CT scan. We considered the possibility that the broken surface wing (Fig. 8A, left), which appears small in CT scans (Fig. 8C), is a small wing; however, in surface view its preserved width, including at the base, its vein spacing, and its attachment to the nut are nearly the same as the more complete surface wing.

*Shorea* is favored over *Hopea* for the dispersed wings as well as the articulated exemplar, although the dispersed wings are somewhat larger, and thus more than one *Shorea* species could be present. In our observation of extant material, *Hopea* cross veins are sparse, whereas *Shorea* cross veins are denser as in these (Fig. 9B) and other *Shorea* fossils (*e.g*., Shi, Jacques & Li, 2014). In addition, the large wing size is far more typical of *Shorea* than *Hopea* species. The enlarged dispersed wings and their well-marked, densely percurrent and variable cross veins resemble some living Brunei species in the Red Meranti group (*S*. subgenus *Rubroshorea* Meijer; see Ashton et al., 2021) that have broad wing bases (*e.g*., *S. ferruginea* Dyer ex Brandis; see Ashton, 1964). A few leaves with potential affinity to *Shorea* were found at Kampong Lugu (see morphotype BR13; Figs. 13D–13F).

Both *Hopea* (Selangan) and *Shorea* (no common name applies to the whole genus; Ashton, 1964) are widespread and diverse in Brunei (Ashton, 1964; Coode et al., 1996) and beyond, although their numbers are drastically reduced due to anthropogenic pressures (Ashton, 2014). Borneo is the center of diversity and endemism for both genera (Ashton, 1982). *Hopea* is a lowland genus of the subcanopy to canopy with over 100 species in total, usually occurring below 800 m elevation from southern India and southern China into the Malay Archipelago to New Guinea; *Shorea* has nearly 200 species, often of dominant to emergent trees in varied lowland habitats (mostly below 1,200 m), from India to the Philippines, Java, and Wallacea (*e.g*., Ashton, 2004). The probable tallest angiosperm tropical tree found globally to date is the Menara Tree, a *Shorea faguetiana* F. Heim of 100.8 m height from the nearby Danum Valley Conservation Area in Sabah (Malaysian Borneo; Shenkin et al., 2019).

cf. Family Malvaceae Juss.

cf. Malvaceae sp. BR05 (Figs. 9E, 9F)

*Exemplar and only specimen*. UBDH F00136 (PW1502-39, from Berakas Beach; Figs. 9E, 9F).

*Distinguishing features*. Morphotype BR05 is cordate with seven basally actinodromous primary veins, compound agrophic veins, and opposite percurrent interior secondaries and tertiaries.

*Description*. Blade attachment marginal. Lamina length > 12 cm, width > 9.7 cm; base cordate; margin not preserved; toothing, lobing, and symmetry unclear. Primary venation basal actinodromous with seven primary veins; agrophic veins compound with robust, apparently unbranched minor secondaries directed toward the margin. One pair of non-interior major secondaries preserved, diverging far above the base. Interior secondaries, becoming tertiary veins distally, are thin, closely spaced, opposite percurrent, and concentric in appearance from joining the primaries at right angles. Higher-order venation reticulate.

*Remarks*. Morphotype BR05 matches the general features of the family Malvaceae Juss., which has many species with cordate bases, basally actinodromous primaries, compound agrophic veins, and opposite percurrent, concentric tertiaries (Carvalho et al., 2011). However, the family identification is not definite without a sufficiently preserved margin to detect potential teeth or lobes, which have distinctive characters in Malvaceae that allow separation from other families with broadly similar features (Carvalho et al., 2011). Species with similar leaves are found in several malvaceous genera in the region today, including *Firmiana* Marsili, *Grewia* L., *Sterculia* L., and *Trichospermum* Blume.

Family Melastomataceae Juss.

Subfamily Melastomatoideae Seringe

**Figure 10:**
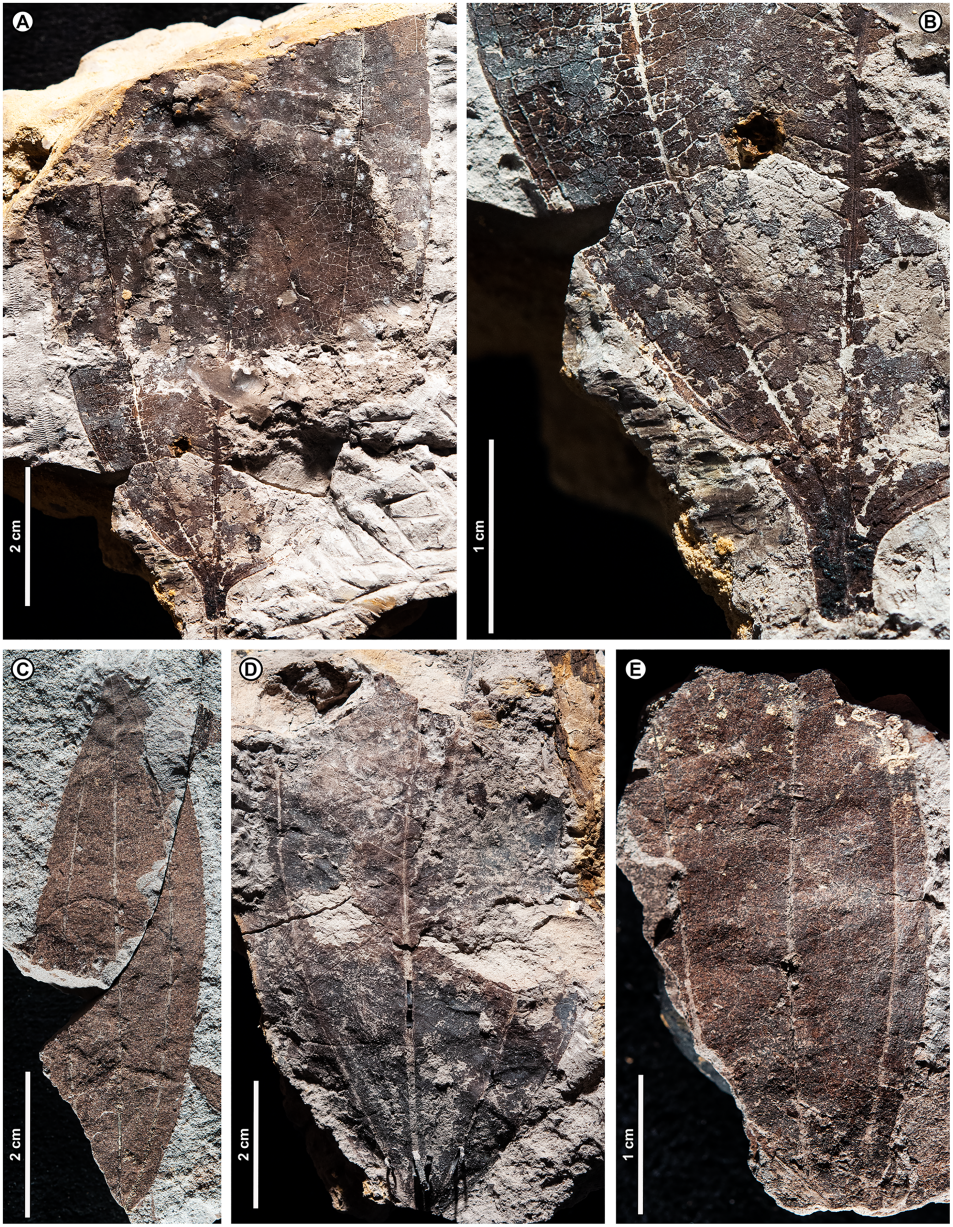
Melastomataceae sp. BR06. (A, B) UBDH F00149a (Kampong Lugu), general aspect and detail of base, preserving perfect-acrodromous primary venation, irregular-percurrent convex interior secondaries (transverse veins), and looped marginal venation; (C) UBDH F00099a (Berakas Beach), blade with high aspect ratio and partial apex preserved, showing all primary veins reaching the apex; (D) UBDH F00275a (Kampong Lugu), preserving similar features to the specimen in A and B; (E) UBDH F00131 (Berakas Beach), fragmentary specimen.

Melastomataceae sp. BR06 (Fig. 10)

*Exemplar specimen*. UBDH F00149a,b (PW1503-13a,b, from Kampong Lugu; Figs. 10A, 10B).

*Additional material*. Two specimens from Berakas Beach and one from Kampong Lugu (Figs. 10C–10E; Appendix 2).

*Distinguishing features*. Morphotype BR06 has an elliptic blade with five basal perfect-acrodromous, unbranched primaries that reach the apex (Fig. 10C) and numerous, closely spaced, percurrent, convex interior secondaries departing the primaries at acute angles. The marginal venation is looped (Fig. 10B).

*Description*. Blade attachment marginal. Lamina length to > 8.7 cm (*n* = 1), width to > 5.6 cm (*n* = 4); estimated length L:W ratio 3.3:1 (*n* = 1); lamina symmetrical. Margin unlobed and entire. Base and apex angle acute; base shape concavo-convex. Primary venation basal perfect-acrodromous with five primaries extending from base to apex, the lateral primaries close to the margin and much thinner than the medials. Secondaries interior, percurrent (scalariform), unbranched, course slightly to markedly convex, spacing slightly irregular, angle to the midvein acute and slightly irregular. Tertiary and higher-order venation irregular reticulate. Marginal ultimate venation in ca. two series of small, well-developed loops flattened inside the margin, in places forming a weak intramarginal vein.

**Figure 11:**
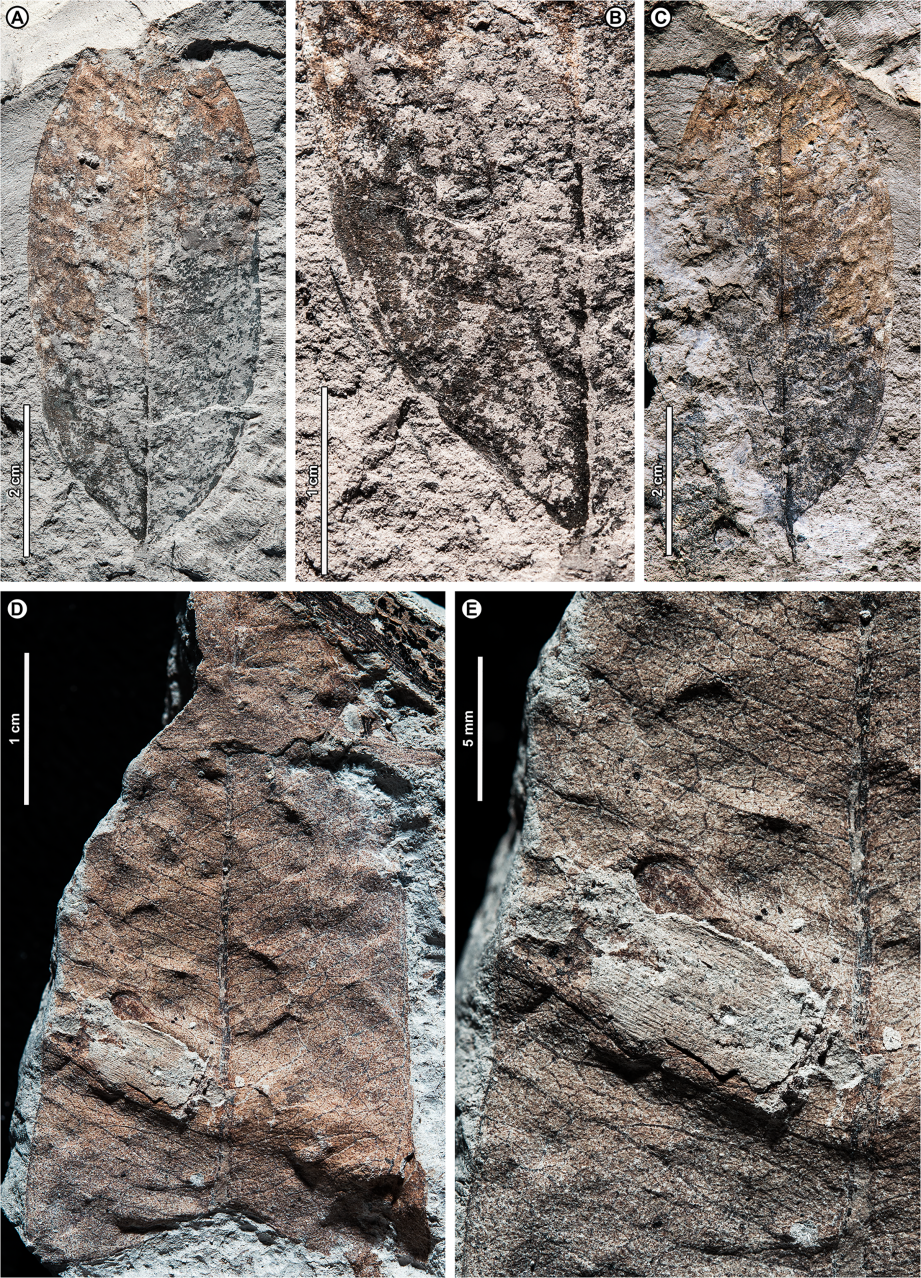
Possible Myrtaceae. Pinnate leaves with numerous secondary and intersecondary veins, intramarginal veins, and reticulate higher-order venation, Berakas Beach. (A–C) cf. Myrtaceae sp. BR07. (A, B) UBDH F00017a, general aspect and detail of left-basal margin. (C) Counterpart F00017b; (D, E) cf. Myrtaceae sp. BR08, UBDH F00129a. (D) General aspect. (E) Detail.

*Remarks*. Morphotype BR06 has several well-known characters of Melastomataceae subfamily Melastomatoideae, which has many species with perfect-acrodromous primaries extending to the apex, ladder-like interior secondaries (transverse veins), and looped marginal venation or an intramarginal vein. Those features, along with a non-cordate base, do not occur together in other families (Carvalho et al., 2021) and make many of the melastomes instantly recognizable in the field (Gentry, 1993). The family is very diverse in Brunei today, with about 25 genera (Coode et al., 1996). Among those, species of *Pternandra* Jack show some similarities to the fossils in general aspect, including their slightly irregular interior-secondary venation patterns (M. Carvalho, 2021, personal communication). Outside of Melastomataceae, the most similar taxon in the living Brunei flora is probably *Anisophyllea* R. Br. ex Sabine (Anisophylleaceae), which also has acrodromous venation with five or more primaries. However, the venation in that genus is much less organized than in Melastomataceae, including irregular basal offsets of the primaries that are very different from the perfect-acrodromous fossils. See *Ziziphus* sp. BR09 for additional comparisons within the fossil assemblage.

cf. Family Myrtaceae Juss.

cf. Myrtaceae sp. BR07 (Figs. 11A–11C)

*Exemplar and only specimen*. UBDH F00017a,b (PW1501-17a,b, from Berakas Beach, Figs. 11A–11C).

*Distinguishing features*. Morphotype BR07 has an oblong blade and a straight base with a ca. 90° angle. It has thin, closely spaced major secondary veins, with flattened loops near the margin forming a weak intramarginal vein, and intersecondary veins with frequency usually one per intercostal area and length less than 50% of subjacent secondaries. Tertiaries are irregular and reticulate.

*Description*. Blade attachment marginal, petiole short. Lamina length 7.3 cm; width 3.0 cm; L:W ratio ca. 2.4:1 (*n* = 1 for all); laminar shape oblong with basal and medial symmetry. Margin unlobed and entire, slightly thickened. Base angle ca. 90°; base shape straight. Apex angle acute. Primary venation pinnate. Major secondaries thin, numerous (ca. 25 pairs), closely and regularly spaced, angle uniform, brochidodromous with flattened loops near the margin grading into an irregular intramarginal vein. Intersecondary veins parallel to major secondaries, length less than 50% of subjacent secondary, frequency usually one per intercostal area, usually terminating in the subjacent secondary. Intercostal and epimedial tertiary veins and quaternary veins irregular reticulate. Marginal ultimate venation looped.

*Remarks*. The general characteristics of the single BR07 specimen, including parallel venation and an intramarginal vein, are typical of several genera of Myrtaceae, such as *Syzygium*, which has over 65 species in Brunei today (Coode et al., 1996). However, as discussed earlier (see *Dryobalanops* sp. BR03), these features appear convergently in several other families, and additional evidence is needed for a definite familial assignment. See morphotypes BR08 and BR21 for further comparisons within the fossil assemblage.

cf. Myrtaceae sp. BR08 (Figs. 11D, 11E)

*Exemplar specimen*. UBDH F00129a,b (PW1502-32a,b, from Berakas Beach; Figs. 11D, 11E).

*Additional specimen*. One specimen from Berakas Beach (Appendix 2).

*Distinguishing features*. The specimens in morphotype BR08 are likely to be fragments of long-elliptic leaves. The thin, dense major secondaries have irregular spacing and nearly uniform angle to the midvein, terminating in a well-marked intramarginal vein. Intersecondaries are present but difficult to distinguish from the random reticulate tertiaries.

*Description*. Lamina elliptic with medial symmetry. Leaf length > 7.0 cm; width 2.4–3.6 cm (*n* = 2). Margin unlobed and entire. Primary venation pinnate. Base and apex not preserved. Major secondaries thin, numerous (>20 pairs preserved), course to the intramarginal vein, spacing irregular, angle nearly uniform. Intersecondaries irregular, reticulating or joining subjacent secondary, difficult to distinguish from the irregular reticulate tertiary veins. Quaternary vein fabric irregular reticulate.

*Remarks*. Comparing morphotype BR08 with BR07 (the other possible Myrtaceae), BR07 has brochidodromous major secondaries, stronger intersecondaries, and a weaker intramarginal vein, whereas BR08 lacks distinct secondary loops, has weaker intersecondaries, and has a stronger intramarginal vein that is closer to the margin; BR08 is also larger than BR07 and apparently long-elliptic. Compared with BR03 (*Dryobalanops*), both BR07 and BR08 have intramarginal veins that are more distinct from the margin and significantly looser tertiary-vein organization. As for BR07, other familial assignments are possible. See morphotype BR21 for additional comparisons.

Family Rhamnaceae Juss.

Genus *Ziziphus* Mill.

**Figure 12:**
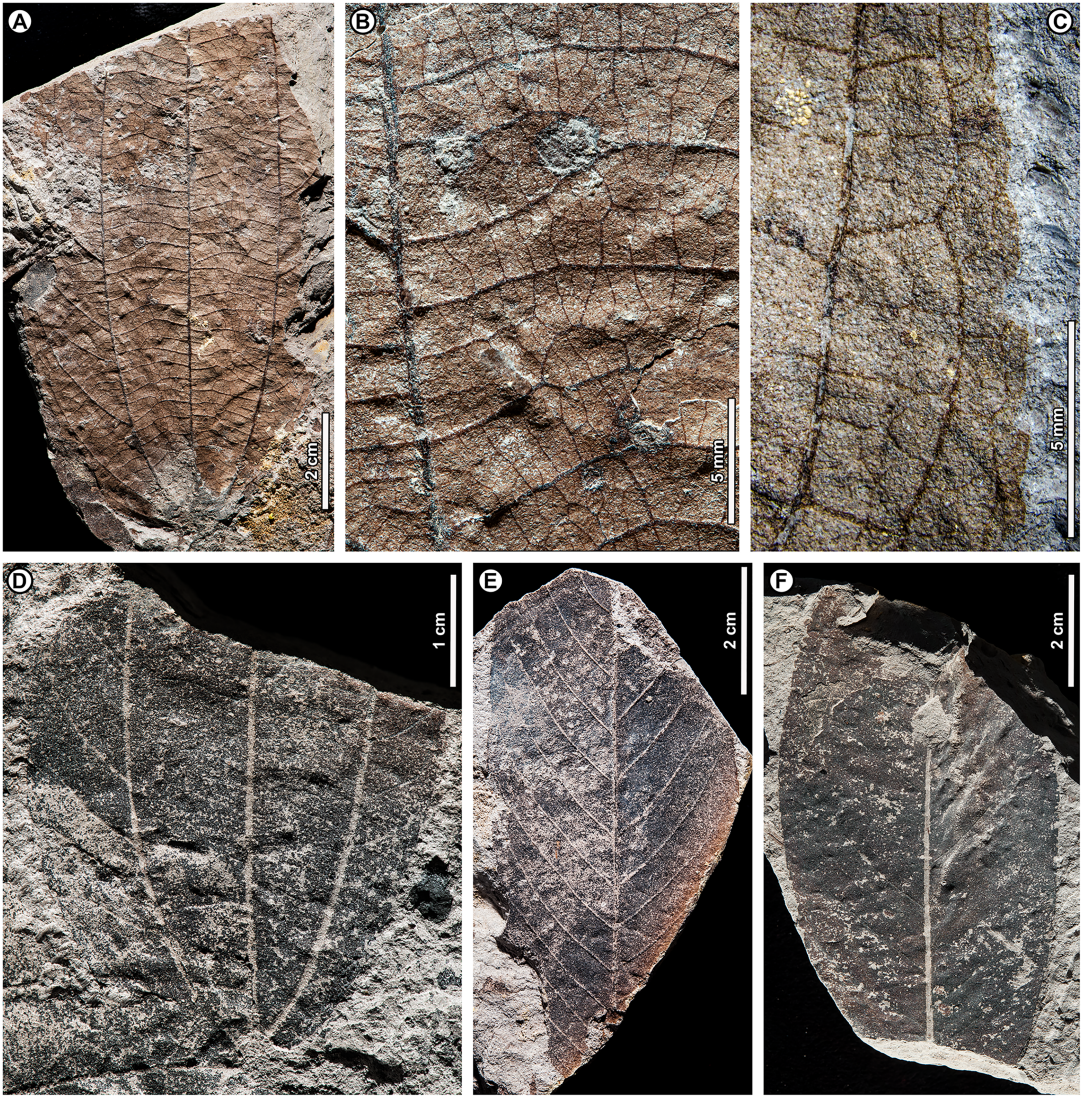
*Ziziphus* sp. BR09 and dicot sp. BR10. All from Berakas Beach. (A–D) *Ziziphus* sp. BR09, with asymmetrical blade, three acrodromous primaries, strong agrophic veins, and all major secondary veins interior (transverse). (A, B) UBDH F00117a, general aspect and venation detail near the center of the blade. (C) Counterpart F00117b, margin detail showing ultimate venation and serrulations. (D) UBDH F00026a. (E, F) Dicot sp. BR10, a likely dipterocarp. (E) UBDH F00058. (F) UBDH F00068.

*Ziziphus* sp. BR09 (Figs. 12A–12D)

*Exemplar specimen*. UBDH F00117a,b (PW1502-20a,b, from Berakas Beach; Figs. 12A–12C).

*Additional material*. One specimen from Berakas Beach (Fig. 12D; Appendix 2).

*Distinguishing features*. Morphotype BR09 has three basal perfect-acrodromous primary veins and is markedly asymmetrical (Figs. 12A, 12D), with tiny marginal serrulations (Fig. 12C). The lateral primaries form agrophic vein complexes with weakly looping minor secondaries; the agrophic-vein field is larger on one side of the blade than the other (Figs. 12A, 12D). All major secondary veins are interior (Fig. 12B), percurrent, thick, well spaced, and slightly irregularly angled; their course is convex, deflected by tertiaries, and nearly perpendicular to the primaries at departure. Tertiary and higher-order venation is clearly visible.

*Description*. Blade attachment marginal, insertion area not preserved. Lamina length to > 10.6 cm, width 3.3–6.6 cm (*n* = 2); lamina shape elliptic and strongly asymmetrical. Margin unlobed and serrulate. Base angle acute; base shape convex. Primary venation basal perfect-acrodromous with three strong primary veins; agrophic veins simple, prominent, weakly looped, developed into a larger field on one side of the blade with more than nine minor secondaries per field. Major secondaries all interior, percurrent, unbranched or branched, thick and apparently raised, departure from primaries nearly perpendicular or slightly acute, spacing wide (ca. 2.5–5.0 mm), angle somewhat irregular, course slightly to markedly convex and deflected at junctions with the tertiaries. Non-interior major secondaries and intramarginal vein absent. Tertiary, quaternary, and quinternary veins irregular reticulate, clearly visible, apparently raised. Marginal ultimate venation a series of small loops inside the margin, giving off short exterior tertiary veins that connect the loops to a thin fimbrial vein. Serrulations minute, closely spaced (ca. 5 per cm), shape straight-convex or convex-convex, vascularized by the exterior tertiary veins and fimbrial vein. Laminar glands, hairs, domatia, and marginal callosities not observed, presumably due to preservation. Small hole-feeding marks present, to ca. 3 mm length (DT1, DT2; Figs. 12A, 12B).

*Remarks*. The markedly asymmetrical blade with three strong acrodromous primaries, well-developed, percurrent interior secondaries, looping agrophic veins, and serrulate margin present a distinctive combination that diagnoses the fossils as Rhamnaceae. These features are considered typical of the ziziphoid genera *Ziziphus*, *Paliurus* Mill., *Ceanothus* L., and a few others, although there is a consensus that these taxa cannot be distinguished using leaf architecture alone (Meyer & Manchester, 1997; Burge & Manchester, 2008; Jud et al., 2017). Nevertheless, *Ceanothus* and *Paliurus* species almost always have some non-interior major secondaries, and their venation is thus quite different from the strongly percurrent, entirely interior major secondaries of the fossils and many *Ziziphus* species. Instead, major secondaries of *Ceanothus* and *Paliurus* may be eucamptodromous or reticulodromous, not reaching the lateral primaries except through branching. Accordingly, placement of the fossils in *Ziziphus* is warranted and consistent with the distribution of 13 living *Ziziphus* species now recognized in Borneo, comprising the most speciose island assemblage for the Old World genus of ca. 80 species (Cahen, Rickenback & Utteridge, 2021). *Paliurus* (Europe to mainland SE Asia) and *Ceanothus* (North America) do not occur in the Malay Archipelago today.

Among the living Borneo species recently revised by Cahen, Rickenback & Utteridge (2021), the fossils most closely resemble *Z. kunstleri* King (including *Z. cupularis* Suess. & Overkott by synonymy) in having very similar well spaced, often-branching interior secondary veins (transverse veins) and well-marked higher-order venation that deflects the interior secondaries at the junctions. Additional shared features include closely comparable blade size and shape, marginal ultimate venation, and density and type of marginal serrulations, as well as the lack of an intramarginal vein. The only significant differences appear to be that the blade of *Z. kunstleri* is more symmetrical and has more numerous minor secondary veins in the agrophic complexes than the fossils. *Ziziphus kunstleri* is a Near Threatened liana distributed in the lowlands of Borneo (including Brunei), Peninsular Malaysia, and Thailand (Cahen, Rickenback & Utteridge, 2021). Consistent with the idea that these leaf fossils could represent lianas, leaf lengths less than 20 cm, as in both fossil specimens, are found in the climbers but not in the arborescent *Ziziphus* species of Borneo (Cahen, Rickenback & Utteridge, 2021).

Within this study, morphotype BR09 is superficially similar, because of its perfect-acrodromous primary venation, only to morphotype BR06 (Melastomataceae). However, BR09 is asymmetrical with three primaries, whereas BR06 is symmetrical with five, and BR06 has lateral primaries near the margin, which is entire; in BR09, well-developed, looping agrophic veins dominate the lateral venation, and the margin is serrulate.

Family *Incertae sedis*

Dicot sp. BR10 (Figs. 12E, 12F)

*Exemplar specimen*. UBDH F00058 (PW1501-58, from Berakas Beach, Fig. 12E).

*Additional material*. Two specimens from Berakas Beach (Fig. 12F; Appendix 2).

*Distinguishing features*. Morphotype BR10 is long-elliptic, with eucamptodromous, regularly spaced, numerous (up to at least 12 pairs) major secondaries, whose angle to the midvein decreases smoothly proximally.

*Description*. Lamina length to > 8.1 cm; width 2–4.7 cm (*n* = 3); lamina shape elliptic with medial symmetry. Base and apex not preserved. Margin unlobed and entire. Primary venation pinnate. Major secondaries eucamptodromous, at least 12 pairs preserved, spacing regular, angle to the midvein smoothly decreasing proximally. Intercostal tertiary veins thin, opposite percurrent, closely spaced.

*Remarks*. Morphotype BR10 is a generalized category representing the combination of near-oblong shape, numerous eucamptodromous and unbranched major secondary veins, and opposite percurrent tertiary veins. Based on these features, BR10 is likely to represent one or more dipterocarp species of uncertain generic affinities.

**Figure 13:**
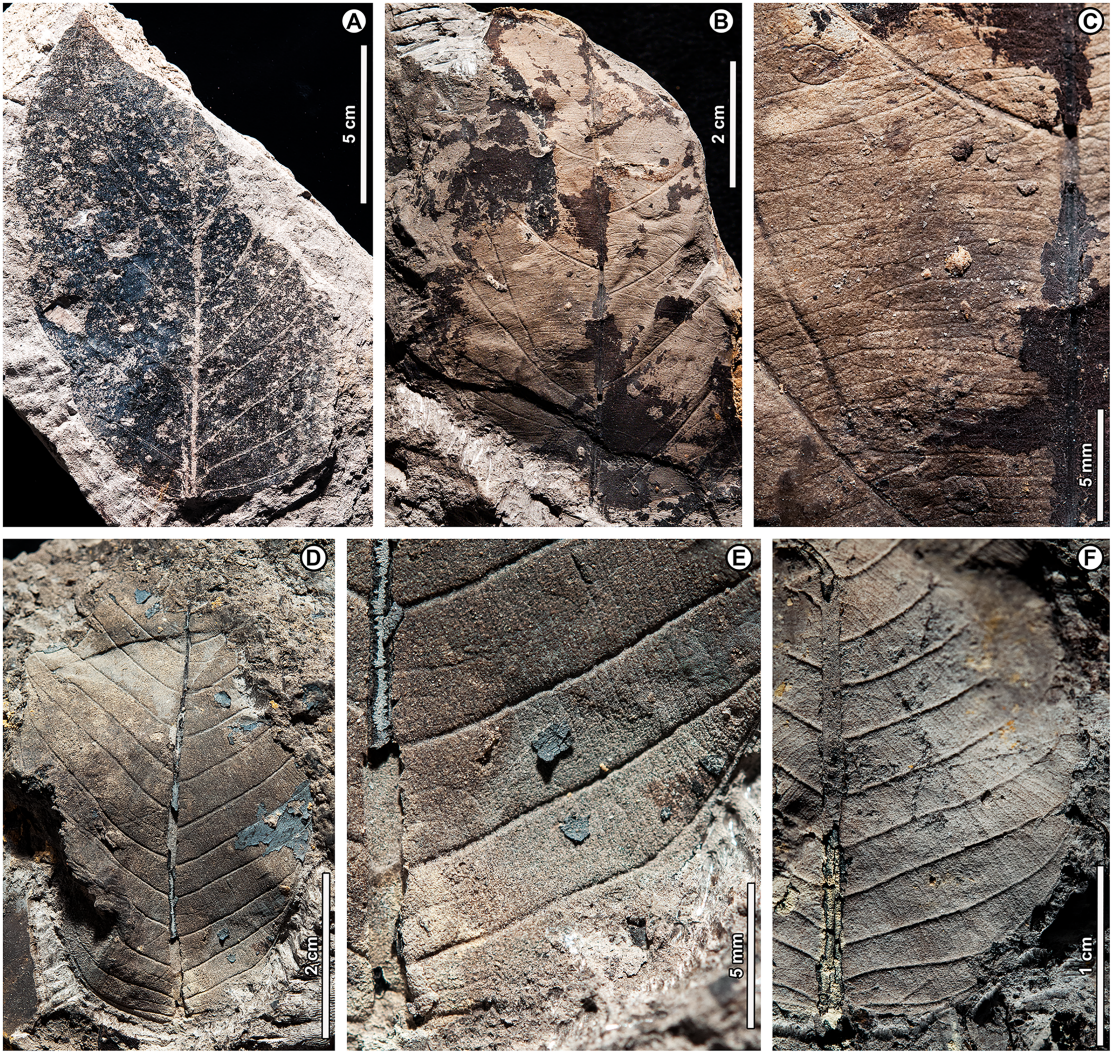
Dicot spp. BR11–BR13. (A) Dicot sp. BR11, UBDH F00037a (Berakas Beach), with major secondary veins abruptly obtuse near the base on one side; (B, C) Dicot sp. BR12, UBDH F00154 (Kampong Lugu), general aspect and venation detail at center of lamina; (D–F) Dicot sp. BR13 (Kampong Lugu), similar to some *Shorea* species, preserving short intersecondary veins and high-angle percurrent tertiary veins. (D, E) UBDH F00336b, general aspect and detail of venation near base. (F) UBDH F00219a, detail near base.

Dicot sp. BR11 (Fig. 13A)

*Exemplar and only specimen*. UBDH F00037a,b (PW1501-37a,b, from Berakas Beach; Fig. 13A).

*Distinguishing features*. Morphotype BR11 has an asymmetrical, subrounded base, with two pairs of basal major secondary veins at an abruptly large angle to the midvein, more so on one side than the other, and thin, opposite percurrent tertiary veins.

*Description*. Lamina length > 16 cm; width 10.6 cm (*n* = 1 for both); lamina shape obovate with asymmetrical base. Margin unlobed and entire. Base angle obtuse; base shape subrounded. Primary venation pinnate. Major secondaries eucamptodromous, ca. eight pairs preserved, spacing decreasing proximally, angle abruptly increasing proximally, more so on one side than the other. Intercostal tertiary veins thin, dense, straight opposite percurrent and obtuse to midvein; epimedial tertiaries opposite percurrent with proximal course perpendicular to midvein, distal course parallel to intercostal tertiaries. Higher-order venation poorly preserved.

Dicot sp. BR12 (Figs. 13B, 13C).

*Exemplar and only specimen*. UBDH F00154 (PW1503-18, from Kampong Lugu; Figs. 13B, 13C).

*Distinguishing features*. Morphotype BR12 is a fragment of a pinnate leaf preserving irregularly spaced and angled secondary veins and strong, numerous, opposite percurrent but irregularly spaced and curved intercostal and epimedial tertiary veins. The tertiaries are nearly perpendicular to the midvein at departure and only slightly increase in angle exmedially.

*Description*. Primary venation pinnate; major secondary spacing and angle irregular (ca. five pairs preserved); length > 8.2 cm, width > 5.2 cm (*n* = 1 for both). Intercostal tertiary veins opposite percurrent, straight or convex, densely but irregularly spaced, obtuse to midvein with variable curvature; tertiary vein angle increasing exmedially. Epimedial tertiaries opposite percurrent; proximal course perpendicular to midvein; distal course parallel to intercostal tertiaries. Quaternary and quinternary vein fabric reticulate. Hole feeding present (DT3; Fig. 13B).

*Remarks*. Even though the single specimen of morphotype BR12 is only partially preserved, it is distinctive within the collection for its strong, numerous, straight opposite percurrent intercostal and epimedial tertiaries and its irregular secondaries, which must be carefully distinguished from linear, vein-like impressions of other material (probably of small twigs) in the fossil. The most similar morphotype here is *Dipterocarpus* sp. BR02, which has regular and much wider secondary and tertiary-vein spacing and a strongly impressed midvein, features lacking in morphotype BR12.

Dicot sp. BR13 (Figs. 13D–13F)

*Exemplar specimen*. UBDH F00336a,b (PW1503-202a,b, from Kampong Lugu; Figs. 13D, 13E).

*Additional specimen*. One specimen from Kampong Lugu (Fig. 13F).

*Distinguishing features*. Morphotype BR13 has strong, regular, eucamptodromous, unbranched major secondaries upturned near the margin, with angle to the midvein increasing proximally and spacing decreasing proximally. Short intersecondary veins are variably present. The tertiary veins have a very high angle and nearly parallel the midvein (Figs. 13E, 13F). The midvein is thick, especially near insertion, suggesting that a broad petiole was present.

*Description*. Blade attachment marginal. Laminar shape elliptic and symmetrical. Leaf length to > 5.4 cm; width to > 3.8 cm; margin unlobed and entire. Primary venation pinnate. Major secondaries eucamptodromous (ca. 12 pairs preserved), upturned near the margin, spacing decreasing proximally, angle increasing proximally. Intersecondaries very short, less than 50% of the subtending secondary length, frequency one per secondary or less. Intercostal tertiary veins straight opposite percurrent, course markedly obtuse to midvein; vein angle decreasing exmedially. Epimedial tertiaries’ proximal course obtuse to midvein, distal course parallel to intercostal tertiaries. Quaternary vein fabric orthogonal reticulate.

*Remarks*. Morphotype BR13 is similar to *Dipterocarpus* sp. BR01 in that they both have elliptic shape, unbranched major secondaries, and opposite percurrent tertiaries. However, BR13 appears to lack plication (as in BR01), and it has basally crowded, obtuse secondaries, short intersecondaries, and very high angle tertiaries, all features lacking in BR01. Although we do not make an assignment here due to the limited material, the identical features just listed are seen in several living *Shorea* species, such as *S. albida* Symington, the dominant tree of northern Borneo peat swamps.

**Figure 14:**
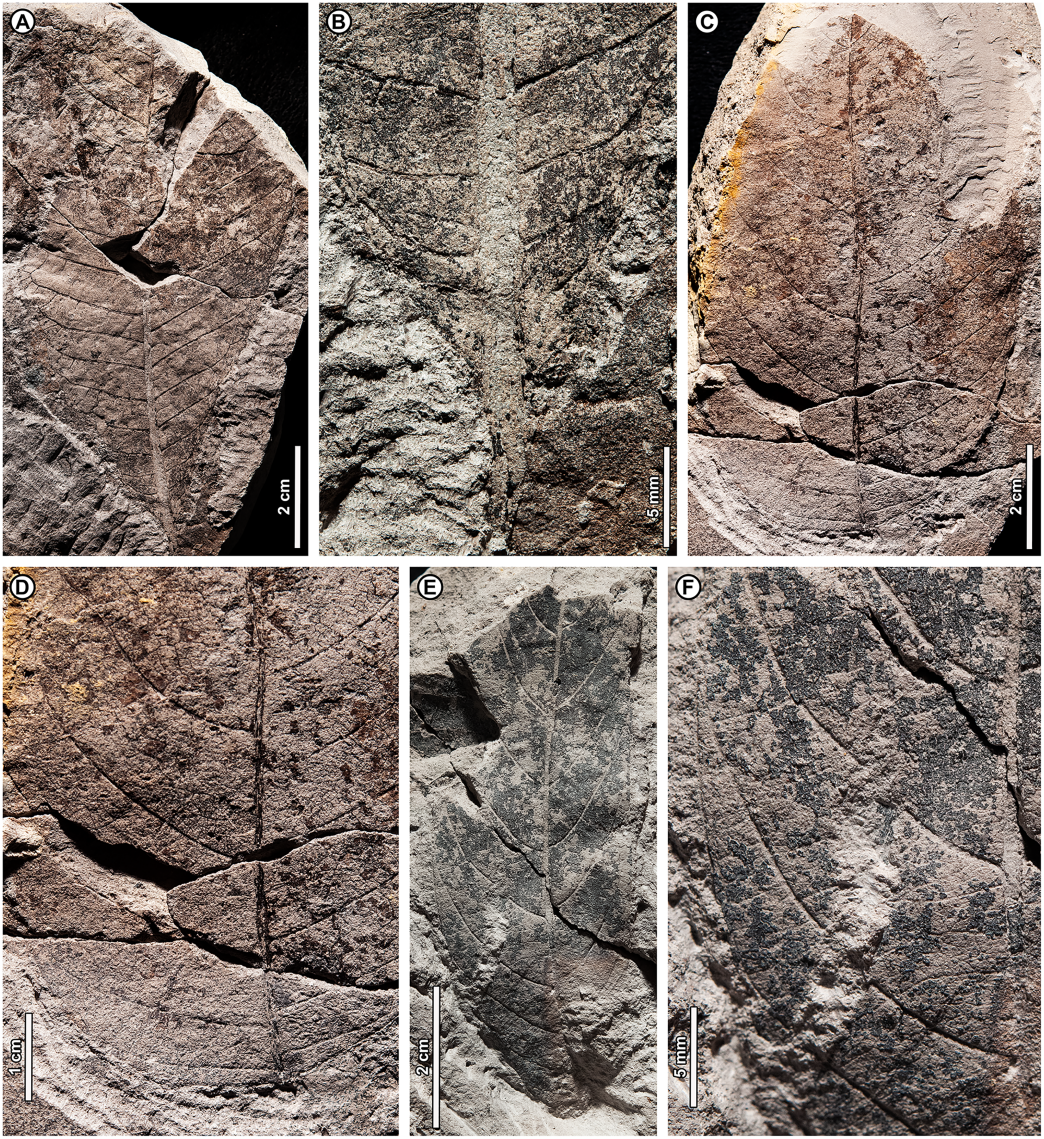
Dicot spp. BR14–BR16. (A, B) Dicot sp. BR14, UBDH F00027 (Berakas Beach), general aspect showing asymmetrical blade, irregular secondary venation, and detail of acute, asymmetrical base; (C, D) Dicot sp. BR15, UBDH F00053a (Berakas Beach), general aspect and detail of rounded, asymmetrical base; (E, F) Dicot sp. BR16, UBDH F00139 (Kampong Lugu), general aspect and detail of margin, upturned major secondary veins, and fimbrial vein.

Dicot sp. BR14 (Figs. 14A, 14B)

*Exemplar and only specimen*. UBDH F00027 (PW1501-27, from Berakas Beach; Figs. 14A, 14B).

*Distinguishing features*. Morphotype BR14 has a straight, acute asymmetrical base. The major secondaries are eucamptodromous to reticulodromous, numerous, weak, often deflected by tertiaries, at slightly different angles on either side of the midvein, basally crowded, and irregularly spaced. Very short intersecondaries are present, frequency one per secondary; the tertiary vein angle decreases near the margin but increases basally to nearly parallel the midvein.

*Description*. Blade attachment marginal, petiole fragment slender. Lamina length > 10.2 cm; width > 6.0 cm (*n* = 1 for both); lamina has medial, basal, and insertion asymmetry. Margin unlobed and entire; base acute. Primary venation pinnate. Major secondaries eucamptodromous or reticulodromous (ca. 10 pairs preserved), deflected at tertiary junctions, spacing basally crowded and irregularly spaced, angle varies on either side of the midvein. Short intersecondaries present. Tertiary veins thin, weakly opposite percurrent, deflected, obtuse to midvein; angle decreasing exmedially and increasing basally to parallel the midvein. Quaternary vein fabric reticulate.

Dicot sp. BR15 (Figs. 14C, 14D)

*Exemplar and only specimen*. UBDH F00053a,b (PW1501-53a,b, from Berakas Beach; Figs. 14C, 14D).

*Distinguishing features*. The base of morphotype BR15 is asymmetrical and smoothly rounded. Major secondaries are eucamptodromous and diverge from the midvein nearly at a right angle, curving smoothly; they become crowded and more obtuse basally and are otherwise widely and irregularly spaced.

*Description*. Lamina length > 10 cm; width > 5.7 cm (*n* = 1 for both). Margin unlobed and entire. Base shape asymmetrical and smoothly rounded; base angle obtuse. Primary venation pinnate; agrophic veins absent. Major secondaries eucamptodromous, in at least 12 pairs, slender, nearly perpendicular to midvein at divergence, smoothly curved, spacing irregular and decreasing proximally and apically, angle increasing proximally. Tertiary and quaternary vein fabric reticulate.

*Remarks*. The most similar morphotype in the collection to BR15 is BR11, which has a less smoothly rounded base, much straighter secondaries, and opposite percurrent (not reticulate) tertiaries.

Dicot sp. BR16 (Figs. 14E, 14F)

*Exemplar and only specimen*. UBDH F00139 (PW1503-3, from Kampong Lugu; Figs. 14E, 14F).

*Distinguishing features*. Morphotype BR16 is long-elliptic and asymmetrical, with eucamptodromous, irregularly spaced major secondaries that recurve apically and are strongly upturned inside the margin. Tertiaries are opposite percurrent, and a fimbrial vein is present.

*Description*. Blade attachment marginal. Lamina length > 7.9 cm; width 2.8 cm (*n* = 1 for both); lamina with medial and basal asymmetry. Margin unlobed and entire. Base angle acute; primary venation pinnate. Major secondaries eucamptodromous, in at least nine pairs, spacing decreasing proximally, angle to the midvein uniform, slightly more acute on one side, strongly upturned distally against the margin. Fimbrial vein present. Intercostal tertiary veins opposite percurrent, obtuse to midvein; angle decreases exmedially. Epimedial tertiaries opposite percurrent, straight or convex; proximal course nearly perpendicular to the midvein; distal course parallel to intercostal tertiaries. Quaternary vein fabric reticulate.

**Figure 15:**
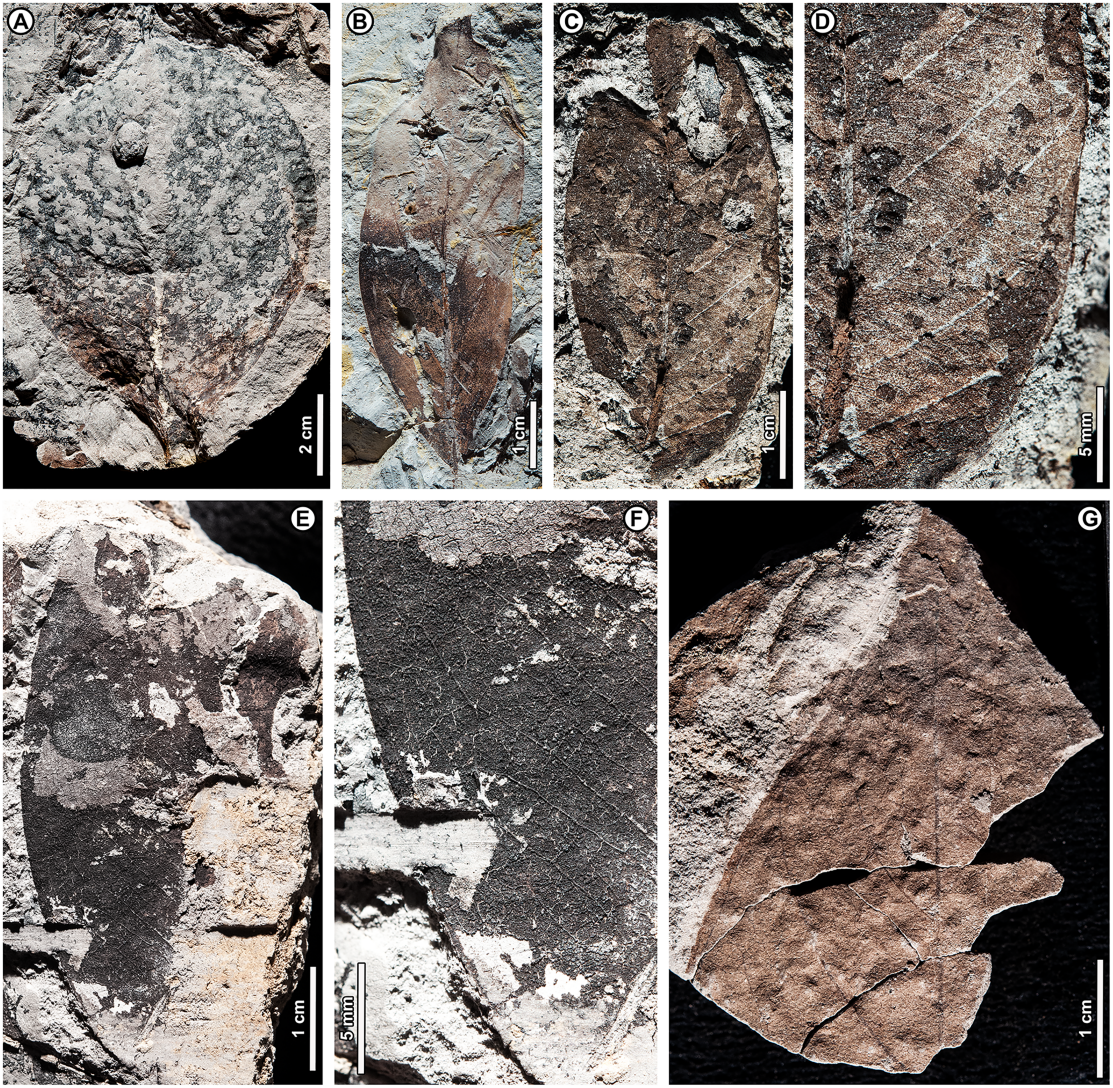
Dicot spp. BR17–BR21. (A) Dicot sp. BR17, UBDH F00056 (Berakas Beach), showing decurrent base and strongly convex margin; (B) Dicot sp. BR18, UBDH F00145a (Kampong Lugu), a likely dipterocarp with densely spaced, opposite-percurrent tertiary veins; (C, D) Dicot sp. BR19, UBDH F00212a (Kampong Lugu), general aspect and margin detail, preserving asymmetrical, elliptic blade and dense, percurrent tertiary venation; (E, F) Dicot sp. BR20, UBDH F00263 (Kampong Lugu), general aspect and detail of leaf margin, preserving egg-shaped, elliptic blade and thin, irregular, reticulodromous secondary venation. (G) Dicot sp. BR21, UBDH F00033 (Berakas Beach), preserving a long-acuminate apex and thin, brochidodromous secondary venation.

Dicot sp. BR17 (Fig. 15A)

*Exemplar and only specimen*. UBDH F00056 (PW1501-56, from Berakas Beach; Fig. 15A).

*Distinguishing features*. Morphotype BR17 has a markedly decurrent base and a convex, smoothly curved, almost rounded margin with a short-acuminate apex; the blade is basally and medially asymmetrical. The major secondaries are brochidodromous with flattened exterior loops.

*Description*. Blade attachment marginal. Lamina shape elliptic with medial and basal asymmetry; margin strongly convex, unlobed, and entire. Lamina length 11.4 cm; width 7.2 cm; L:W ratio 1.6:1 (*n* = 1 for all). Base decurrent; apex short-acuminate. Primary venation pinnate. Major secondaries slender, brochidodromous with flattened loops against the margin. Higher-order venation poorly preserved.

Dicot sp. BR18 (Fig. 15B)

*Exemplar and only specimen*. UBDH F00145a,b (PW1503-9a,b, from Kampong Lugu; Fig. 15B).

*Distinguishing features*. Morphotype BR18 is narrow-elliptic with an acute base and apex, and it has strong, regular, eucamptodromous major secondaries with regular spacing and a uniform, low angle to the midvein. The tertiaries are thin and opposite percurrent.

*Description*. Blade attachment marginal. Lamina elliptic and symmetrical. Lamina length 7.2 cm; width 2.3 cm; L:W ratio 3.1:1 (*n* = 1 for all); margin unlobed and entire. Base angle acute; base shape straight; apex angle acute. Primary venation pinnate. Major secondaries eucamptodromous without branching, in at least 10 pairs, spacing regular, angle to the midvein uniformly low. Intercostal tertiary veins straight opposite percurrent, thin and dense; angle obtuse to midvein, decreasing exmedially, increasing basally. Epimedial tertiaries opposite percurrent; proximal course nearly perpendicular to midvein; distal course parallel to intercostal tertiaries. Quaternary vein fabric reticulate. Small galls present (DT11; Fig. 15B).

*Remarks*. Based on its regular, robust secondary veins and dense, opposite-percurrent tertiaries, the specimen is probably a small leaf of Dipterocarpaceae and could have affinities with *Dipterocarpus* sp. BR01. We report morphotype BR18 separately because its secondary vein angle is lower, no traces of plications are visible, and the blade is much smaller and narrower than BR01.

Dicot sp. BR19 (Figs. 15C, 15D)

*Exemplar and only specimen*. UBDH F00212a,b (PW1503-78a,b, from Kampong Lugu; Figs. 15C, 15D).

*Distinguishing features*. Morphotype BR19 is an elliptic, asymmetrical microphyll. Major secondaries are thin and eucamptodromous with mostly uniform angles, increasing slightly at the base; secondary spacing decreases proximally. Tertiary veins are thin, dense, and opposite percurrent, with angle increasing basally.

*Description*. Insertion marginal. Lamina elliptic and asymmetrical. Leaf length 5.5 cm; width 2.5 cm; L:W ratio 2.2:1 (*n* = 1 for all); margin unlobed and entire. Primary venation pinnate. Major secondaries eucamptodromous, at least 11 pairs, spacing decreasing and angle increasing slightly toward the base. Intercostal tertiary veins opposite percurrent, convex, obtuse to midvein; vein angle increasing basally. Epimedial tertiaries opposite percurrent; proximal course nearly perpendicular to midvein; distal course parallel to intercostal tertiaries. Quaternary and quinternary vein fabric irregular reticulate.

*Remarks*. Morphotype BR16 has some features of BR19, but BR16 has a longer aspect, a fimbrial vein, and irregular secondaries that are upturned against the margin, unlike BR19.

Dicot sp. BR20 (Figs. 15E, 15F)

*Exemplar and only specimen*. UBDH F00263 (PW1503-129, from Kampong Lugu; Figs. 15E, 15F).

*Distinguishing features*. Morphotype BR20 is microphyll in size and elliptic, egg-shaped with a smoothly curved margin. It has numerous weak, thin major secondaries with irregular courses and angles that reticulate approaching the margin.

*Description*. Laminar shape elliptic, smoothly curved. Leaf length > 6.0 cm; width 3.9 cm; margin unlobed and entire. Primary venation pinnate. Major secondaries thin, reticulodromous with irregular course, spacing irregular, angle to the midvein irregular, in at least 25 pairs. Intercostal tertiary veins reticulate to weakly percurrent, obtuse to midvein; vein angle decreasing exmedially.

Dicot sp. BR21 (Fig. 15G)

*Exemplar and only specimen*. UBDH F00033 (PW1501-33, from Berakas Beach; Fig. 15G).

*Distinguishing features*. Morphotype BR21 has a long-acuminate apex. Major secondaries are thin and brochidodromous, with flattened loops close to the margin. Tertiary fabric is reticulate.

*Description*. Only the apical region is preserved. Length > 5.0 cm; width > 3.1 cm (*n* = 1); margin unlobed and entire. Apex angle acute; apex shape long-acuminate. Primary venation pinnate; major secondaries brochidodromous, thin, widely spaced with loops flattened near the margin. Third-through fifth-order vein fabrics irregular reticulate. Ultimate marginal venation looped.

*Remarks*. Although only the apical portion of a single specimen is preserved and its affinities are unknown, morphotype BR21 is distinct from comparable morphotypes, such as BR07 and BR08, due to its long-acuminate apex and thin, widely spaced, brochidodromous major secondaries with flattened loops near the margin. Morphotype BR17 also has some similarities, but its secondary veins are denser and higher-angled, with a shorter apex than BR21.

Family Araceae Juss.

Tribe Monstereae Engl.

Genus *Rhaphidophora* Hassk.

**Figure 16:**
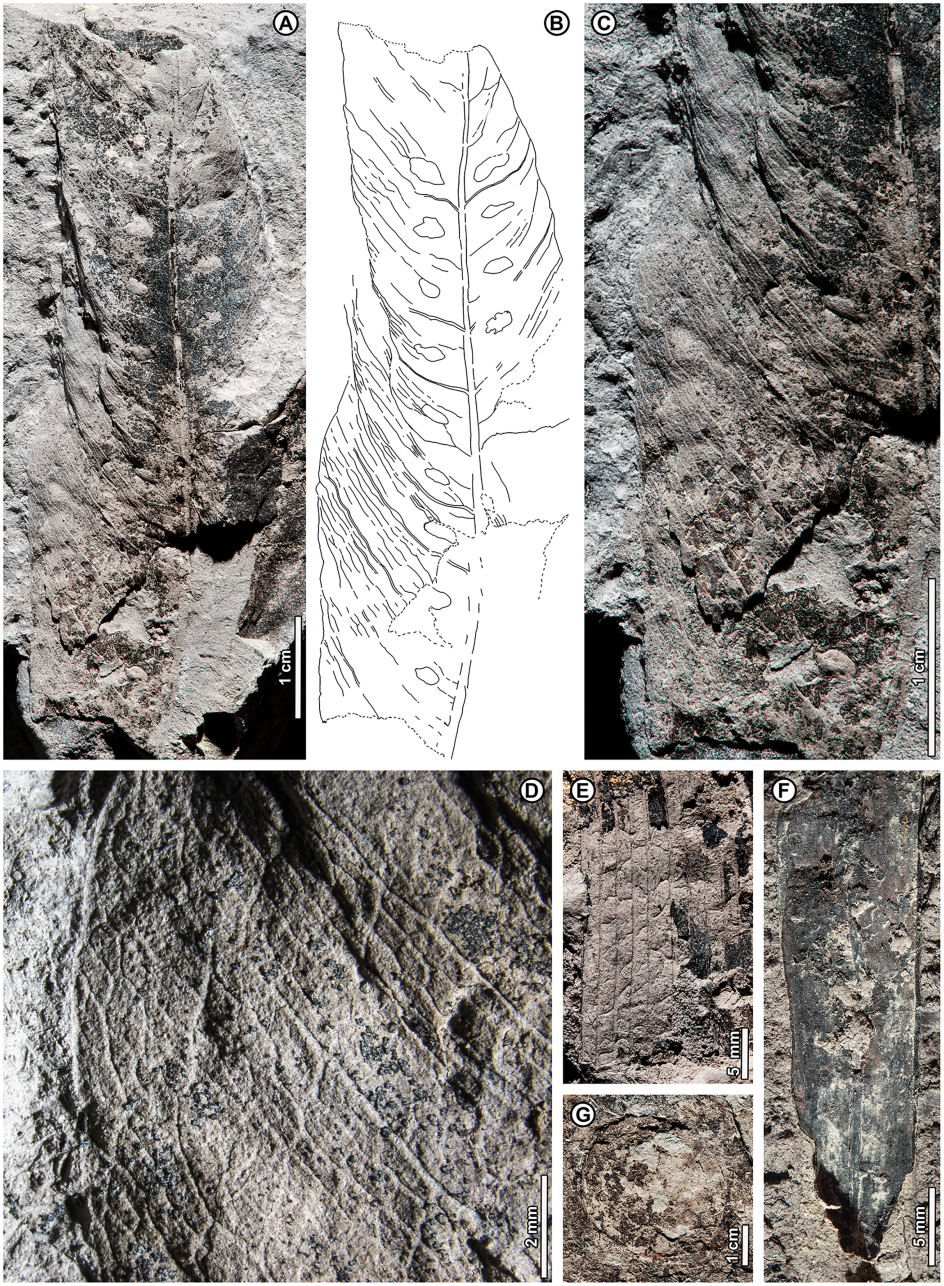
Monocots and *incertae sedis*. (A–D) *Rhaphidophora* sp. BR22, UBDH F00039a (Berakas Beach). (A, B) General aspect and corresponding overlay drawing, showing quilted texture, elliptic shape, stout costa, primary (lateral) veins, subparallel-to-reticulating interprimary veins, and conspicuous elliptical perforations arrayed in longitudinal rows on either side of the costa. (C) Detail of preserved margin, showing quilted texture and weak interprimary veins. (D) Detail near middle of preserved left margin showing reticulate higher-order venation and fimbrial vein; (E) possible palm-leaf fragment, cf. Arecaceae sp. BR23, UBDH F000237a (Kampong Lugu), showing parallel veins and numerous oblique, variably angled and curved cross-veins; (F) Monocot sp. BR24, UBDH F00338b (Kampong Lugu), preserving strap-shaped, coalified blade, thick midvein, and thickened margin; (G) Possible fruit or seed of unknown affinities (BR25), UBDH F00041a (Berakas Beach).

*Rhaphidophora* sp. BR22 (Figs. 16A–16D)

*Exemplar and only specimen*. UBDH F00039a,b (PW1501-39a,b, from Berakas Beach; Figs. 16A–16D).

*Distinguishing features*. The blade of BR22 is long-elliptic and unlobed, with a thick costa and quilted relief; primary veins (per Boyce, 2001, also termed lateral veins) are moderately spaced, separated by numerous subparallel to reticulating interprimary veins that are deflected by and merge with random reticulate higher-order veins (Figs. 16A–16D). Primary veins, interprimary veins, and reticulum terminate in the fimbrial vein (Fig. 16D). Elliptical perforations (fenestrae) are present near the costa (Figs. 16A–16C), located between or interrupting the primary veins, orientation roughly parallel to the primary veins.

*Description*. Laminar shape long-elliptic, with a quilted texture; leaf length > 7.6 cm; width > 2.7 cm (*n* = 1). Margin unlobed and entire. Costa thick. Primary veins in at least 12 pairs, spacing regular, separated by ca. 6-7 thin interprimary veins oriented subparallel to the primaries, course deflected by and merging with reticulum of higher-order veins. Primary veins, interprimary veins, and reticulum terminate in the fimbrial vein. Base and apex not preserved. Perforations irregular-ellipsoidal, dimensions somewhat distorted preservationally, long axis length 2.4–3.5 mm, short axis length 1.0–1.9 mm; aligned in two rows on either side of, near to, and parallel to the costa; each oriented roughly parallel to or interrupting primary veins; frequency one per primary vein.

*Remarks*. Much of the basal preserved portion of the single specimen (as seen in Fig. 16C) is folded down vertically into the sediment on one side, distorting the margin and leaf shape, although this area best preserves the venation and the quilted texture. The quilted texture is also observed in the unfolded apical portion of the fossil, confirming this feature. The overall architecture of the specimen is typical of some Araceae genera, including the combination of a narrow-elliptic blade with a thick costa, numerous parallel primary and interprimary veins, and a fimbrial vein (Mayo, Bogner & Boyce, 1997). Several of the ca. 28 genera of Araceae in the Brunei flora (Coode et al., 1996) have species with very similar features to the fossil, among them *Homalomena* Schott, *Rhaphidophora*, *Schismatoglottis* Zoll. & Moritzi, and *Scindapsus* Schott (Mayo, Bogner & Boyce, 1997; Boyce, 2001; Boyce & Yeng, 2016).

The presence of numerous elliptical fenestrae on the blade (Figs. 16A–16C) is typical of some araceous genera (Mayo, Bogner & Boyce, 1997) and adds confidence that the fossil does not belong to a different family with somewhat similar leaves that lack this morphology, such as Zingiberaceae. We considered whether these features could represent insect damage, but they lack reaction rims and are too regular in size and placement to represent hole feeding. The only possibility related to insect activity would be bud feeding, but this would require the unlikely scenario for this family of the leaf being scrolled longitudinally in the bud rather than laterally and for two feeding events to have occurred in parallel. Accordingly, clear examples of bud feeding that we found in herbarium material of *Rhaphidophora* are oriented perpendicular to the costa (not parallel as seen here) and show substantial (ca. 3–6x) secular size increase related to instar stages (not more or less constant size as seen here).

Araceous genera with commonly well-fenestrated leaves, the majority in Tribe Monstereae, mainly occur in the Neotropics and Africa and have very different leaf shapes (*i.e*., sagittate, pinnatifid) from the fossil (Mayo, Bogner & Boyce, 1997). However, there are two monsteroid genera in the Brunei and regional flora with well-perforated leaves in some species: *Amydrium* Schott, which has an entirely different leaf form from the fossil, and *Rhaphidophora*, which, as just discussed, has very similar leaf architecture. Like the fossil, *Rhaphidophora* has several species with numerous ellipsoid perforations very close to or intersecting the costa, as well as a quilted blade appearance due to the thick costa and primary veins (*i.e*., *R. foraminifera* (Engl.) Engl. and others; see Mayo, Bogner & Boyce, 1997: plates 14A, 109B; Boyce, 2001: figs. 6, 7; Boyce et al., 2010: plate 6A, B). Thus, by simple elimination, the fossil can be provisionally assigned to *Rhaphidophora*, a widespread, West African and South and East Asian genus with about 100 species mostly of herbaceous root-climbers, including 16 species in Borneo (Boyce, 2001; Boyce et al., 2010).

cf. Family Arecaceae Bercht. & J.Presl

cf. Arecaceae sp. BR23 (Fig. 16E)

*Exemplar and only specimen*. UBDH F00237a,b (PW1503-103a,b, from Kampong Lugu; Fig. 16E).

*Distinguishing features*. Morphotype BR23 is a blade fragment preserving at least ten parallel veins and irregular, oblique, sinuous cross veins.

*Description*. Morphotype BR23 is represented by a single leaf fragment, ca. 3.7 cm by 2.0 cm, with no base or margins visible. At least ten parallel veins are preserved, along with oblique, variably angled, curved to sinuous cross veins.

*Remarks*. The irregular, oblique, sinuous cross veins connecting parallel veins are characteristic of palms (Arecaceae) but not diagnostic without other information (*e.g*., Gómez-Navarro et al., 2009). The most similar fossils in the collection are the fruit wings of *Shorea* sp. BR04, the largest of which is about half the width of the BR23 fragment, with much closer vein spacing and comparatively straight, not sinuous cross veins.

Monocot sp. BR24 (Fig. 16F)

*Exemplar and only specimen*. UBDH F00338a,b (PW1503-204a,b, from Kampong Lugu; Fig. 16F).

*Distinguishing features*. BR24 is strap-shaped and thick-textured with a strong midvein and thickened margin; numerous but indistinct parallel veins cross the blade longitudinally.

*Description*. Base and apex not preserved, the lone specimen almost completely coalified, length > 7.7 cm, width 0.9 cm (*n* = 1). Blade strap-shaped, linear. Margin prominently thickened, possibly revolute. Midvein prominent. Parallel veins numerous, with close spacing, course parallel to midvein.

*Remarks*. Morphotype BR24’s strap-like shape, single midvein, and parallel venation indicate a monocot, but no diagnostic features of lower taxa are preserved.

*Incertae sedis*

Unknown sp. BR25 (Fig. 16G)

*Exemplar specimen*. UBDH F00041a,b (PW1501-41a,b, from Berakas Beach; Fig. 16G).

*Distinguishing features and description*. BR25 is a probable fruit or seed of rounded shape with diameter about 4.0 cm.

*Remarks*. Morphotype BR25, though indistinct, is the only probable non-dipterocarp fruit or seed fossil in the collection.

## Discussion

This article reports the first fossil compression floras from Brunei and the first paleobotanical collections of significant size from the Cenozoic of the Malay Archipelago for a century or more. The macroflora and co-occurring palynoflora provide valuable new information about late Cenozoic coastal rainforests in Borneo. The data show that the ancient floristic composition and ecosystem structure were very similar to modern, including the characteristic dipterocarp dominance, thus heightening the historical conservation significance of the living analog forests. All floristic and physiognomic (*e.g*., only one species toothed, some leaves very large) indicators strongly support an everwet, megathermal climate, consistent with palynological data that show the persistence of aseasonal equatorial rainforests in Borneo and equatorial Malesia through the Pliocene and Pleistocene (Morley & Morley, 2013; Morley & Morley, 2022; de Bruyn et al., 2014). In a broad sense, this work affirms Wallace’s (1869) idea that the fossil leaves he observed in the region were nearly identical to modern taxa, although we do not know which fossil deposits Wallace observed. The nearly-entirely different compositional and abundance signals from pollen *vs* leaves demonstrate their complementary value and the importance of compression fossils for clarifying the region’s Cenozoic vegetation history, which had been known almost entirely from palynological data and some fossil woods (see Introduction). Our study shows that a great deal of information can be extracted from the overlooked compression floras of the Asian wet tropics through extensive collecting and careful preparation, despite the general likelihood of poor preservation. For instance, less than a fourth of the collected slabs had identifiable material at Berakas Beach, but that fraction contributed significantly to the floristic information recovered (Table 1).

### General reconstructions

We reconstruct the Berakas Beach assemblage as the remains of a (most likely) early Pliocene, coastal fern-dominated swamp with mostly still, at times brackish water and restricted marine influence. Near the depocenter, diverse semi-aquatic and terrestrial ferns with a wide variety of presumed life habits were present, along with several types of lycophytes and enormous numbers of fungi. Nearby mangroves included *Nypa*, *Avicennia*, and *Rhizophora*. Dipterocarps dominated the adjacent lowland coastal rainforest, including *Dipterocarpus*, *Dryobalanops*, and *Shorea*, and other families included Ctenolophonaceae, Lecythidaceae (*Barringtonia*), Rubiaceae, Rutaceae, and Sapotaceae. Probable non-arborescent (understory, parasitic, or climbing) angiosperms included Loranthaceae, Melastomataceae, *Merremia*, *Ziziphus*, *Calamus*, and *Rhaphidophora*. *Podocarpus* and *Ilex* pollen occurrences potentially indicate more remote hill forests or additional components of the lowland communities.

The Plio-Pleistocene of Kampong Lugu had a low-energy, mangrove-swamp depocenter with abundant rotting, submerged wood, large numbers of fungi, and diverse mangrove taxa, including *Nypa*, *Sonneratia*, *Avicennia*, *Rhizophora*, and the mangrove fern *Acrostichum*. Other ferns were varied and abundant, with several likely life habits from semi-aquatic to ground cover, tree ferns, climbers, and epiphytes. The bordering lowland rainforest was dominated by dipterocarps, especially *Dryobalanops*, and a large-leaved form similar to extant *Dipterocarpus confertus*. Additional rainforest elements included Combretaceae, Euphorbiaceae, Lecythidaceae (*Barringtonia*), Malvaceae, Melastomataceae, Myristicaceae, Rubiaceae, and Sapotaceae. Potential hill-forest contributions included *Podocarpus*, *Myrica*, and *Ilex*.

### Dipterocarp dominance and fossil history

Dipterocarp macrofossils are clearly dominant at both fossil sites, even though the total sample size was somewhat limited by preservation (Table 1). Dipterocarps comprised 79% of all identifiable specimens (Table 1), providing the first localized evidence of ancient dipterocarp-dominated forests in Malesia and establishing new regional macrofossil records for *Dipterocarpus*, *Dryobalanops*, and *Shorea*. This result complements earlier observations that Neogene woods in the region, mainly from Java, are often dipterocarpaceous (van Slooten, 1932; Schweitzer, 1958; Mandang & Kagemori, 2004; see Introduction) as well as the ubiquity of dipterocarp-sourced amber in Neogene Brunei sediments (Kocsis et al., 2020). Moreover, it is likely that many of the unclassified morphotypes (*e.g*., morphotypes BR13 and BR18) and unplaced leaves also represent dipterocarps, especially those that are elliptical with regular, straight secondary veins and opposite percurrent tertiary venation.

This report is the only direct comparison from Malesia of dipterocarp macrofossil and pollen relative abundances using unbiased counts from the same strata. In sharp contrast to the macrofossils, the palynology assemblages yielded only rare specimens of dipterocarp pollen, always <1% abundance in all four samples from both sites (Appendix 1). This result is notable in light of the central role of dipterocarp pollen in the historical understanding of biogeography and assembly of Southeast Asian lowland rainforests (*e.g*., Morley, 2000; Morley, 2018; Ashton et al., 2021). The pollen of dipterocarps is a simple but distinctive tricolpate type, and its rarity in the Brunei assemblages could be explained by a combination of very little pollen reaching the depositional sites and further dilution from the dominant, locally sourced fern spores and fungal bodies. Among other authors (see Introduction), Hamilton et al. (2019) inferred that dipterocarp pollen production, pollination strategy, and pollen preservation lead to its species being commonly underrepresented in the fossil record. Bera (1990) performed one of the only relevant actualistic studies on Sal (*Shorea robusta*) in Madhya Pradesh (India), finding that the trees produce enormous amounts of pollen that are nevertheless poorly dispersed and progressively degraded in the air column until the grains are nearly all filtered out at soil level. From all these observations, dipterocarp pollen abundance, although valuable data, might have little general relationship to the actual standing biomass of dipterocarps through time, a hypothesis that should be further tested using other macrofossil deposits. The absence of dipterocarp pollen probably means very little about whether or not dipterocarps were present at a site, but the preservation of any amount of dipterocarp pollen may well be linked to the dominance of the family, as seen here. For now, our work helps to make sense of the frequent observation that dipterocarp pollen is absent or very rare in many Southeast Asian pollen assemblages where it might be expected to be very abundant (see Introduction), including in Brunei (Anderson & Muller, 1975; Roslim et al., 2021).

Several of the dipterocarp macrofossil occurrences are significant, in addition to the general novelty of the collection and its location. The *Dryobalanops* leaves (Fig. 7), the most abundant fossils in the whole sample (Table 1), complement Neogene wood records of *Dryobalanoxylon* from Sumatra, Indonesian Borneo, and Java (where the genus is extinct; *e.g*., Schweitzer, 1958; Srivastava & Kagemori, 2001; Mandang & Kagemori, 2004). Outside Indonesia, Neogene *Dryobalanoxylon* woods are reported from southern India, Thailand, and Vietnam (summarized by Bande & Prakash, 1986; Biswas, Khan & Bera, 2019). Thus, other than an interesting anecdotal report of a *Dryobalanops*-like leaf from the Neogene of West Java (van Slooten, 1932), the new fossils appear to represent the only non-wood macrofossil record of this ecologically significant genus of large tropical trees.

The *Shorea* fruits from Berakas Beach (Figs. 8, 9A–9D) may also be a first for the Malesian region, although abundant evidence indicates a history of the now-hyperdiverse genus (Ashton et al., 2021) since the middle Eocene. Well-preserved *Shorea* winged*-*fruit fossils come from the late Eocene Maoming Basin (Guangdong Province, China; Feng et al., 2013; also see Ashton et al., 2021), from Miocene sediments in Fujian Province, China (Shi, Jacques & Li, 2014), and Arunachal Pradesh and Gujarat, India (Khan & Bera, 2010; Shukla, Guleria & Mehrotra, 2012; Khan et al., 2016). Middle Eocene *Shoreoxylon* woods from Myanmar (Licht et al., 2014) are the oldest definitive macrofossil evidence of Dipterocarpoideae (see Kooyman et al., 2019) and belong to Section *Shorea*. *Shoreoxylon* is also known from Myanmar, Thailand, several areas of India, and Indonesia (Java and Sumatra; Schweitzer, 1958; Wheeler, 2011). Neogene leaves attributed to *Shorea* are widely reported from India (summarized in Feng et al., 2013; Khan et al., 2016). *Shorea*-type pollen is found in Malesia from the Oligocene onwards (Muller, 1981; Mohd Yakzan et al., 1996; Huang et al., 2021), including the Miocene of Brunei (Anderson & Muller, 1975). More recent work from Brunei with SEM imaging reported grains of *Shorea* sp. and *S*. sp. cf. *S*. *albida* (*S. albida* is the dominant peat-swamp species in the region today) from the Miocene Seria, Miri, and Belait formations (Roslim et al., 2021).

The *Dipterocarpus* fossils at both sites (Figs. 5, 6) represent at least two species, including one with notably large leaf size and architecture comparable, in the living Brunei flora, with the emergent tree *D. confertus*. To our knowledge, these are the only recently reported non-wood macrofossils of the genus from the Malay Archipelago. However, several fossil leaves and fruits from the region are attributed to *Dipterocarpus* in historical literature in need of revision (see Introduction; summarized in Khan et al., 2020), and some illustrations show the characteristic plicated foliage (*e.g*., Miocene of Sumatra: Kräusel, 1929a: plate 6.1). Palynological data support the presence of the genus in northern Borneo since the Oligocene (Muller, 1981; Ashton et al., 2021).

Outside Malesia, *Dipterocarpus* fruits are known from middle Miocene strata of southeastern China (Fujian Province; Shi & Li, 2010; Shi, Jacques & Li, 2014; Chen et al., 2021). Woods and pollen potentially related to *Dipterocarpus* are found far beyond the current range, including pollen from the Late Cretaceous (Maastrichtian) of Sudan (Morley, 2018; Ashton et al., 2021; Chen et al., 2021). Many leaf fossils attributed to *Dipterocarpus* are reported from the Neogene Siwalik sequence in India, Nepal, and Bhutan (summarized in Khan et al., 2020).

*Dipterocarpus* leaf fossils are most convincing when the typical architecture of straight, regular, robust secondaries and opposite percurrent tertiary veins (features found in several plant groups) are combined with the taxonomically restricted feature of visible plications, as seen in our fossils and several previous examples (Kräusel, 1929a; Srivastava et al., 2017). Khan et al. (2020) recently assigned leaf fossils to *Dipterocarpus* from Deccan sediments, close to the Cretaceous-Paleogene boundary in Madhya Pradesh, central India, and considered these specimens as evidence for the popular out-of-India model for the introduction of Dipterocarpoideae into Asia (*e.g*., Dutta et al., 2011). However, the fossils lack plications and have comparatively thin and irregular secondary venation, unlike most living *Dipterocarpus*; thus, they could belong to many other taxa despite some similarities in the cuticle.

A few comments on dipterocarp origins are warranted in light of recent reviews of this fascinating topic (Kooyman et al., 2019; Ashton et al., 2021; Cvetković et al., 2022). The dipterocarps are widely held to have originated on the supercontinent of Gondwana and to have arrived in Asia *via* the post-Gondwanan movements of India or Africa (*e.g*., Ashton et al., 2021 and references therein). However, the idea of Gondwanan origins is, so far, lacking any direct support from paleobotany. The Gondwana hypothesis, by definition, requires that dipterocarps were present on the Gondwana supercontinent by ca. 110 Ma (latest Early Cretaceous), before India-plus-Madagascar and Africa began to separate from the remaining landmass (*e.g*., Jokat et al., 2021; Scotese, 2021). However, the literature regarding out-of-Gondwana origins for dipterocarps seems to omit that Early Cretaceous deposits have been sampled across Gondwana for decades, and no fossil dipterocarps or likely relatives have been reported from hundreds of publications (among many others, Archangelsky, 1963; Banerji, 2000; Mohr & Friis, 2000; McLoughlin, Pott & Elliott, 2010; Nagalingum & Cantrill, 2015; Monje-Dussán et al., 2016). Importantly, angiosperms in Gondwanan Early Cretaceous floras are rare, show early stages in the evolution of leaf organization and other characters, and are not allied with derived eudicot families (Mohr & Friis, 2000; Cúneo & Gandolfo, 2005; Nagalingum & Cantrill, 2015; Coiro et al., 2020; Pessoa, Ribeiro & Jud, 2021). All confirmed reports of fossil dipterocarps and related taxa in Africa and India (*e.g*., Ashton et al., 2021) are from much younger, post-Gondwanan strata, and even the Maastrichtian *Dipterocarpus*-type pollen from Sudan (Morley, 2018) is ca. 40 million years younger than Africa’s separation from Gondwana.

The current lack of Gondwanan dipterocarp fossils does not rule out the often-conflated idea that dipterocarps evolved in post-Gondwanan, pre-India-collision India or Africa (*i.e*., ca. 110–50 Ma). The primary evidence for the presence of the family in India and Africa during that interval is palynological (Morley, 2018; Prasad et al., 2018; Ashton et al., 2021; Bansal et al., 2022; Mishra et al., 2022) and quite compelling, appearing to eliminate the rival idea of Asian dipterocarp origins (*e.g*., Shukla, Mehrotra & Guleria, 2013; Srivastava et al., 2014). However, nearly all potentially supporting macrofossil and geochemical evidence has been contested (Shukla, Mehrotra & Guleria, 2013; Kooyman et al., 2019). A recent, comprehensive study of fossil woods from the Deccan Traps found no dipterocarp specimens among 47 anatomically preserved species (Wheeler et al., 2017), and the family has not appeared in any of a large number of recent studies of silicified reproductive material from the Deccans that used advanced three-dimensional imaging techniques (*e.g*., Manchester et al., 2019; Matsunaga et al., 2019). The richness of the debate and the varied evidence presented seem sure to lead to many years of discoveries on the topic of dipterocarp origins.

### Other significant occurrences

The Melastomataceae specimens (Fig. 10) may be the only reliable Asian fossil record of the diverse family of ca. 5,000 species, of which ca. 3,500 are Neotropical (Carvalho et al., 2021). The melastomes have an indistinct pollen type and, despite the striking, perfect-acrodromous leaf architecture seen in many species, a notably poor leaf-fossil record globally that was primarily concentrated in North America and Europe (Renner, Clausing & Meyer, 2001; Morley & Dick, 2003; Carvalho et al., 2021). However, Carvalho et al. (2021) recently reported the oldest record of the family, from the Paleocene (ca. 60–58 Ma) Bogotá Formation of Colombia, which was then in Gondwanan South America. In Asia, the only prior records of the family are leaves of *Melastomaceophyllum* sp. from the Miocene of Labuan Island (Geyler, 1887) and *M. geyleri* from the late Miocene of Sumatra (Kräusel, 1929a; see also van Gorsel, 2020). The first of these is published only as a line drawing of a small leaf fragment that, pending examination of the corresponding type, does not resemble Melastomataceae. The second, *M. geyleri*, is published both as a line drawing and a small photograph. Although the line drawing resembles Melastomataceae, Kräusel’s photograph clearly shows thickened lateral veins that are not typical of the family; the overall venation pattern more closely resembles some Rhamnaceae such as *Zizyphus* (discussed next).

The new *Ziziphus* leaves from Brunei (Figs. 12A–12D) appear to be the first reliable fossil record of the family Rhamnaceae in Malesia and contribute to the biogeographic understanding of the ziziphoid Rhamnaceae (Correa et al., 2010; Hauenschild et al., 2018). There are no previous reports of fossil flowers, fruits, pollen, or wood of Rhamnaceae from the Malay Archipelago (*e.g*., Jud et al., 2017). Prior leaf records are limited to historical reports of Neogene “*Rhamnus*” and “*Ceanothus*” from Java, in need of revision (Göppert, 1864; Crié, 1888; see van Konijnenburg-van Cittert, van Waveren & Jonkers, 2004). Rhamnaceae fossils are widely distributed in mainland South and East Asia and are primarily attributed to other extinct or extant genera, such as *Berhamniphyllum* and *Paliurus*; *Ziziphus* records include Eocene fruits from Gujarat (India), Pliocene woods from Rajasthan (India), and a Pleistocene fruit from Thailand (for summaries, see Burge & Manchester, 2008; Jud et al., 2017; Zhou et al., 2020). As discussed earlier, *Ziziphus*-like isolated leaf fossils are well known from the Paleogene of western North America and elsewhere, but their affinities to the genus are uncertain (*e.g*., Burge & Manchester, 2008; Correa et al., 2010; Manchester, 2014).

The presence of the significant understory and climbing family Araceae and the monsteroid genus *Rhaphidophora* in the fossil flora also appears to be novel for Malesia. Zuluaga, Llano & Cameron (2019) recently reviewed the biogeography and scarce fossil history of monsteroids worldwide. Some of the more reliable records related to living monsteroid genera are seed fossils of *Epipremnum* from the Oligocene and Neogene of Europe (see Madison & Tiffney, 1976), and the global pollen record suggests a deeper history for some genera (Hesse & Zetter, 2007).

## Conclusions

We report two new late Cenozoic compression assemblages from Brunei Darussalam, a nation with extraordinarily biodiverse and intact tropical rainforests. The new plant fossils are the first from that country and the first Cenozoic compression floras from the Malay Archipelago in the modern era. We also report co-occurring palynofloras, and both the macro- and microfossils are unbiased collections. Our results, most broadly, show that the principal features of northern Borneo’s coastal vegetation (*e.g*., Thia-Eng, Loke Ming & Sadorra, 1987; Wong & Kamariah, 1999) have changed little for at least 4–5 million years. Dipterocarps overwhelmingly dominate both macrofossil assemblages, showing for the first time from compression floras, which record localized paleoecological information, that the dipterocarp-dominated rainforests that define lowland forest structure throughout Malesia are ancient. At least three genera (*Dipterocarpus*, *Dryobalanops*, and *Shorea*) and four species of dipterocarps are present, and dipterocarps represent 79% of all identifiable macrofossils. All other elements identified are also present in the living Brunei flora and include the first reliable macrofossil occurrences for the region of Melastomataceae, Rhamnaceae (*Ziziphus*), and Araceae (*Rhaphidophora*).

Rich palynofloras from the same strata as the leaves detail fern- and mangrove-swamp depositional environments with input from adjacent tropical rainforests and diverse, well-structured communities. The pollen data provide a large number of taxon occurrences that complement the macrofloras, with few overlaps. Dipterocarp pollen is notably rare, at less than 1% abundance. Thus, our work directly tests and supports the idea that the low representation of dipterocarp pollen in many regional assemblages results from significant taphonomic bias, providing a caveat for palynological studies. Macrofossils offer an outstanding opportunity to assess patterns of dipterocarp diversity, abundance, and dominance through time and, more broadly, the evolution of the modern vegetation structure and dominance patterns of Southeast Asia. Our discovery of dipterocarp-dominated coastal rainforests in Borneo from 4–5 million years ago raises the conservation significance of their highly threatened yet still strikingly diverse and ecologically foundational living analogs.

## Appendix

**Appendix 1:** Palynological occurrences.

Fossil Taxon	Group	Likely source	Berakas Beach, S6	Berakas Beach, S8	Kampong Lugu, S1	Kampong Lugu, S2
**Freshwater Algae**						
*Botryococcus* spp.	Botryococcaceae	Botryococcaceae	23	47	7	7
*Chomotriletes* spp.	Oedogoniaceae	Oedogoniaceae	+	+	6	18
*Pediastrum* spp.	Hydrodictyaceae	Hydrodictyaceae	0	0	+	0
Algal body	Zygnemataceae?	Zygnemataceae?	0	3	0	0
Algal body (smooth leiosphere)	Zygnemataceae?	Zygnemataceae?	0	1	0	0
**Bryophyta**						
*Stereisporites* sp.	Possible bryophyte	Bryophyte?	0	+	0	0
**Lycopodiopsida**						
*Isoetes* sp.	Isoetaceae	*Isoetes*	0	+	0	0
*Foveotriletes* sp.	Lycopodiaceae	*Huperzia*	+	+	+	0
*Lycopodiacidites* sp.	Lycopodiaceae	*Lycopodiella*	0	1	0	0
*Lycopodiumsporites* sp.	Lycopodiaceae	*Lycopodium*	+	0	0	1
*Selaginella* sp.	Selaginellaceae	*Selaginella*	0	1	0	0
**Polypodiopsida**						
*Asplenium* sp.	Aspleniaceae	*Asplenium*	0	0	+	0
*Blechnum* sp.	Blechnaceae	*Blechnum*	0	+	+	0
*Scolocyamus magnus*	Blechnaceae	*Stenochlaena*	+	1	0	0
*Stenochlaenidites papuanus*	Blechnaceae	*Stenochlaena*	0	*+*	0	0
*Stenochlaenidites* sp.	Blechnaceae	*Stenochlaena*	0	0	0	+
*Verrucatosporites usmensis*	Blechnaceae	*Stenochlaena*	+	9	+	3
*Ctenitis* sp.	Dryopteridaceae	*Ctenitis*	1	0	+	0
Gleicheniaceae spp.	Gleicheniaceae	Gleicheniaceae	0	0	+	+
*Hymenophyllum* sp.	Hymenophyllaceae	*Hymenophyllum*	0	0	+	0
*Crassoretitriletes vanraadshoveni*	Lygodiaceae	*Lygodium*	0	0	+	+
*Osmunda* sp.	Osmundaceae	*Osmunda*	+	0	0	+
*Drynaria* sp.	Polypodiaceae	*Drynaria*	0	0	0	+
*Verrucatosporites favus*	Polypodiaceae	*Polypodium*	7	16	4	9
*Verrucatosporites* spp.	Polypodiaceae	*Polypodium*	9	10	8	2
*Acrostichum* sp.	Pteridaceae	*Acrostichum*	0	0	+	+
*Magnastriatites howardii*	Pteridaceae	*Ceratopteris*	0	+	0	0
*Pteris* spp.	Pteridaceae	*Pteris*	3	3	1	+
*Concavisporites* sp.	Lindsaeaceae?	*Lindsaea*?	0	0	0	+
*Cyathidites* spp.	Dennstaedtiaceae?	*Pteridium*?	12	19	35	22
*Laevigatosporites* spp.	Thelypteridaceae?	*Thelypteris*?	47	130	52	37
*Microfoveolatisporis* sp.	Unknown	Unknown	0	0	0	+
*Cicatricosisporites* sp.	Schizaeaceae?	Unknown	0	Rwk	0	0
**Gymnosperms**						
*Podocarpus* sp.	Podocarpaceae	*Podocarpus*	1	3	+	+
*Pityosporites* sp.	Unknown	Unknown	Rwk	0	0	0
Taxodioideae	Taxodioid Cupressaceae	Taxodioid Cupressaceae (transported)	+	+	0	0
**Angiosperms**						
*Ilex* sp.	Aquifoliaceae	*Ilex*	0	+	1	+
*Dicolpopollis* sp.	Arecaceae	*Calamus*	+	0	0	0
*Monocolpopollenites* sp.	Arecaceae	Possibly a palm	+	0	+	0
*Nypa* sp.	Arecaceae	*Nypa*	0	+	+	+
*Proxapertites* sp.	Arecaceae?	Questionably a palm	1	0	0	0
Palm spp. (echinate)	Arecaceae	Palm	0	1	1	5
*Spinizonocolpites echinatus*	Arecaceae	Palm	0	Rwk	0	0
*Avicennia* sp.	Avicenniaceae	*Avicennia*	+	+	+	0
Combretaceae spp.	Combretaceae	Combretaceae	0	0	5	1
*Perfotricolpites digitatus*	Convolvulaceae	*Merremia*	0	+	+	0
*Corsinipollenites* sp.	Ctenolophonaceae	*Ctenolophon*	+	+	0	0
Dilleniaceae sp.	Dilleniaceae	Dilleniaceae	0	+	0	0
Dipterocarpaceae spp.	Dipterocarpaceae	Dipterocarpaceae	+	1	+	0
*Croton* sp.	Euphorbiaceae	*Croton*	0	0	+	0
Euphorbiaceae sp.	Euphorbiaceae	Euphorbiaceae	3	1	0	0
*Praedapollis* sp.	Fabaceae?	Fabaceae?	1	0	0	0
*Retitricolpites amapaensis*	Fabaceae?	Fabaceae?	0	+	+	0
*Rostriapollenites robustus*	Lecythidaceae	*Barringtonia*	+	1	1	+
Loranthaceae sp.	Loranthaceae	Loranthaceae	0	+	0	0
*Florschuetzia levipoli*	Lythraceae	*Sonneratia*	0	0	1	0
*Bombacacidites* sp.	Malvaceae	Bombacoideae	0	+	0	0
*Discoidites* sp.	Malvaceae	*Brownlowia*	1	2	1	2
*Myrica* sp.	Myricaceae	*Myrica*	0	0	+	+
*Myristica* sp.	Myristicaceae	*Myristica*	+	+	+	+
*Polycolpites* sp.	Onagraceae?	Onagraceae?	0	+	0	0
*Persicarioipollis* sp.	Polygonaceae	*Polygonum*	+	0	+	0
*Zonocostites ramonae*	Rhizophoraceae	*Rhizophora*	+	+	19	9
*Lasianthus* sp.	Rubiaceae	*Lasianthus*	0	1	+	0
Rutaceae sp.	Rutaceae	Rutaceae	0	+	0	0
*Palaquium* sp.	Sapotaceae	*Palaquium*	+	+	+	+
Sapotaceae spp.	Sapotaceae	Sapotaceae	1	1	+	+
*Clavainaperturites* sp.	Unknown	Unknown	0	0	+	0
Echiperiporate sp.	Unknown	Unknown	+	+	0	0
Echitriporate sp.	Unknown	Unknown	0	0	0	+
*Gemmatricolporites psilatus*	Unknown	Unknown	+	0	0	0
Gemmate sp.	Unknown	Unknown	0	0	0	+
*Psilatricolporites* spp.	Unknown	Unknown	8	8	5	3
Retitricolporate sp.	Unknown	Unknown	1	0	0	0
*Retitriporites* sp.	Unknown	Unknown	0	+	0	0
*Tetracolporites* sp.	Unknown	Unknown	+	1	0	0
*Triorites* sp.	Unknown	Unknown	0	+	0	0
Tricolporate spp.	Unknown	Unknown	7	5	0	+
Triporate sp.	Unknown	Unknown	+	0	0	0
*Triporopollenites* sp.	Unknown	Unknown	0	0	+	0
**Fungal Bodies**						
Fungal hyphae	Fungal mycelia	Fungal mycelia	114	12	130	146
Hyphopodia sp.	Fungal mycelia	Fungal mycelia	0	+	0	0
*Asterostromella investiens*	Agonomycetes	Fungal spore	0	0	+	0
*Dendromyceliates splendus*	Agonomycetes	Fungal spore	0	0	+	+
*Diporisporites* sp.	Amerospores	Fungal spore	1	0	1	0
Form-A of Jarzen & Elsik, 1986	Amerospores	Fungal spore	+	+	0	0
*Hypoxylonites* spp.	Amerospores	Fungal spore	2	1	1	2
*Inapertisporites* spp.	Amerospores	Fungal spore	21	11	7	7
*Mediaverrunites* spp.	Amerospores	Fungal spore	9	8	5	6
*Monoporisporites* spp.	Amerospores	Fungal spore	+	+	+	+
*Spegazzinia tessarthra*	Amerospores	Fungal spore	0	0	+	0
*Lirasporis* sp.	Dictyospores	Fungal spore	+	0	0	0
*Staphlosporonites* sp.	Dictyospores	Fungal spore	+	0	0	0
*Dicellaesporites* spp.	Didymospores	Fungal spore	5	1	0	1
*Dyadosporites* spp.	Didymospores	Fungal spore	1	+	+	+
*Fusiformisporites* sp.	Didymospores	Fungal spore	1	0	+	0
*Cirrenalia pygmea*	Helicospores	Fungal spore	0	0	5	7
*Involutisporonites foraminus*	Helicospores	Fungal spore	0	0	0	1
*Ingoldian* type	Hyphomycetes	Fungal spore	0	0	+	0
*Brachysporisporites* sp.	Phragmospores	Fungal spore	1	0	0	0
*Alleppeysporonites* sp.	Phragmospores	Fungal spore	+	0	0	0
*Multicellaesporites* sp.	Phragmospores	Fungal spore	1	0	0	0
*Multicellites* sp.	Phragmospores	Fungal spore	0	0	1	1
*Pluricellaesporites* sp.	Phragmospores	Fungal spore	1	0	0	0
*Quilonia* sp.	Phragmospores	Fungal spore	+	0	+	+
*Meliolinites* sp.	Sordariomycetes	Fungal spore	0	0	1	1
*Frasnacritetrus* sp.	Staurospores	Fungal spore	0	0	+	+
Fungal fruiting body	Microthyriaceous fungi	Fungal fructification	+	0	0	5
*Callimothallus* sp.	Microthyriaceous fungi	Fungal fructification	1	+	0	0
*Desmidiospora* sp.	Microthyriaceous fungi	Fungal fructification	0	+	0	0
*Microthyriacites* sp.	Microthyriaceous fungi	Fungal fructification	0	0	0	+
*Parmathyrites* sp.	Microthyriaceous fungi	Fungal fructification	+	0	0	0
*Phragmothyrites serratus*	Microthyriaceous fungi	Fungal fructification	9	+	0	2
*Phragmothyrites serratus* (germling)	Microthyriaceous fungi	Fungal fructification	+	0	0	3
*Phragmothyrites* spp.	Microthyriaceous fungi	Fungal fructification	4	0	0	+
**Marine Microplankton**						
*Adnatosphaeridium* sp.	Dinoflagellata	Dinocyst	0	Rwk	0	0
*Impletosphaeridium* sp.	Dinoflagellata	Dinocyst	0	0	0	+
*Operculodinium* sp.	Dinoflagellata	Dinocyst	+	0	0	+
*Spiniferites* sp.	Dinoflagellata	Dinocyst	0	1	0	+
*Leiosphaeridia* sp.	Acritarcha	Acritarch	0	0	+	+
*Tasmanites* sp.	Prasinophyceae	Green Algae	0	+	2	+
Microforaminiferal test lining	Foraminifera	Microforam lining	+	0	+	+

**Note:**

Reported abundances are based on an initial count of 300 specimens, after which the slide was scanned for additional taxa, denoted by a plus symbol (+) when present. Rwk, considered reworked from older strata.

**Appendix 2:** Paleobotanical inventory.

UBDH	Locality	Field no.	Morphotype	Comments
F00001	PW1501	1	Un (Unidentified)	Several leaf fragments
F00002	PW1501	2	Un	–
F00003	PW1501	3	Un	–
F00004	PW1501	4	Un	–
F00005a,b	PW1501	5a,b	Un	–
F00006a,b	PW1501	6a,b	Un	–
F00007a,b	PW1501	7a,b	BR01, Un	Several leaf fragments
F00008	PW1501	8	Un	–
F00009a,b	PW1501	9a,b	Un	–
F00010	PW1501	10	BR10, Un	–
F00011	PW1501	11	Un	Several leaf fragments
F00012a,b	PW1501	12a,b	Un	–
F00013	PW1501	13	Un	–
F00014	PW1501	14	Un	Margin damaged (not toothed)
F00015	PW1501	15	Un	–
F00016	PW1501	16	Un	Possible insect mine, serpentine, faintly preserved
F00017a,b	PW1501	17a,b	BR07	BR07 exemplar (Figs. 11A–11C)
F00018a,b	PW1501	18a,b	Un	–
F00019	PW1501	19	Un	Several leaf fragments
F00020a,b	PW1501	20a,b	Un	–
F00021	PW1501	21	Un	–
F00022	PW1501	22	BR04	Figure 9C
F00023	PW1501	23	Un	–
F00024	PW1501	24	Un	–
F00025	PW1501	25	Un	–
F00026a,b	PW1501	26a,b	BR09, Un	Figure 12D
F00027	PW1501	27	BR14	BR14 exemplar (Figs. 14A, 14B)
F00028	PW1501	28	Un	–
F00029	PW1501	29	Un	–
F00030	PW1501	30	BR01	–
F00031	PW1501	31	BR01	–
F00032a.b	PW1501	32a,b	Un	–
F00033	PW1501	33	BR21	BR21 exemplar (Fig. 15G)
F00034	PW1501	34	Un	–
F00035a,b	PW1501	35a,b	Un	–
F00036	PW1501	36	Un	–
F00037a,b	PW1501	37a,b	BR11	BR11 exemplar (Fig. 13A)
F00038a,b	PW1501	38a,b	Un	–
F00039a,b	PW1501	39a,b	BR22, Un	BR22 exemplar (Figs. 16A–16D)
F00040a,b	PW1501	40a,b	Un	–
F00041a,b	PW1501	41a,b	BR25	BR25 exemplar (Fig. 16G)
F00042a,b	PW1501	42a,b	Un	–
F00043	PW1501	43	Un	–
F00044a,b	PW1501	44a,b	Un	–
F00045	PW1501	45	Un	–
F00046	PW1501	46	Un	–
F00047	PW1501	47	Un	–
F00048	PW1501	48	Un	Several leaf fragments
F00049a,b	PW1501	49a,b	Un	–
F00050	PW1501	50	Un	–
F00051	PW1501	51	Un	–
F00052	PW1501	52	Un	Hole feeding (DT2)
F00053a,b	PW1501	53a,b	BR15	BR15 exemplar (Figs. 14C, 14D)
F00054	PW1501	54	Un	–
F00055	PW1501	55	Un	–
F00056	PW1501	56	BR17	BR17 exemplar (Fig. 15A)
F00057a,b	PW1501	57a,b	Un	–
F00058	PW1501	58	BR10	BR10 exemplar (Fig. 12E)
F00059	PW1501	59	Un	Several leaf fragments
F00060a,b	PW1501	60a,b	Un	–
F00061	PW1501	61	Un	–
F00062	PW1501	62	Un	–
F00063	PW1501	63	Un	Two leaf fragments, one similar to *Calophyllum*
F00064a,b	PW1501	64a,b	Un	–
F00065	PW1501	65	Un	–
F00066	PW1501	66	Un	–
F00067	PW1501	67	Un	–
F00068	PW1501	68	BR10	Figure 12F
F00069	PW1501	69	Un	–
F00070a,b	PW1501	70a,b	Un	–
F00071	PW1501	71	Un	–
F00072	PW1501	72	Un	–
F00073	PW1501	73	Un	–
F00074	PW1501	74	Un	–
F00075	PW1501	75	BR08	–
F00076	PW1501	76	BR01	–
F00077	PW1501	77	Un	–
F00078a,b	PW1501	78a,b	BR03, Un	–
F00079	PW1501	79	Un	–
F00080a,b	PW1501	80a,b	Un	Hole feeding (DT2)
F00081	PW1501	81	Un	–
F00082a,b	PW1501	82a,b	Un	–
F00083	PW1501	83	Un	Several leaf fragments
F00084a,b	PW1501	84a,b	Un	Hole feeding (DT2)
F00085a,b	PW1501	85a,b	BR01	–
F00086	PW1501	86	Un	Several leaf fragments
F00087	PW1501	87	Un	–
F00088	PW1501	88	Un	Hole feeding (DT2)
F00089	PW1501	89	Un	–
F00090a,b	PW1501	90a,b	BR01, Un	BR01 exemplar (Fig. 5B), plus several Un leaf fragments on block
F00091a,b	PW1501	91a,b	Un	Several leaf fragments
F00092a,b	PW1501	92a,b	Un	Skeletonization (DT16)
F00093	PW1501	93	Un	–
F00094	PW1501	94	Un	–
F00095	PW1501	95	Un	Hole feeding (DT2)
F00096	PW1501	“upgully”	BR01, Un	A few meters upstream of PW1501, not at same horizon (Fig. 5D)
F00097a,b	PW1501	FS1a,b	BR04	BR04 exemplar (Fig. 8)
F00098a,b	PW1502	1a,b	Un	–
F00099a,b	PW1502	2a,b	BR06	Figure 10C
F00100	PW1502	3	Un	–
F00101	PW1502	4	Un	–
F00102a,b	PW1502	5a,b	Un	–
F00103	PW1502	6	Un	–
F00104a,b	PW1502	7a,b	Un	–
F00105	PW1502	8	Un	–
F00106	PW1502	9	Un	Hole feeding (DT2)
F00107	PW1502	10	Un	Hole feeding (DT1)
F00108a,b	PW1502	11a,b	Un	Gall (DT11). Several leaf fragments on block.
F00109a,b	PW1502	12a,b	Un	Several leaf fragments
F00110a,b	PW1502	13a,b	BR04	Figures 9A, 9B
F00111a,b	PW1502	14a,b	BR04	Figure 9D
F00112	PW1502	15	Un	Hole feeding (DT1)
F00113a,b	PW1502	16a,b	Un	Surface feeding (DT31)
F00114a,b	PW1502	17a,b	Un	–
F00115a,b	PW1502	18a,b	Un	–
F00116a,b	PW1502	19a,b	Un	–
F00117a,b	PW1502	20a,b	BR09	BR09 exemplar (Figs. 12A–12C); hole feeding (DT1, DT2)
F00118a,b	PW1502	21a,b	Un	–
F00119a,b	PW1502	22a,b	Un	–
F00120a,b	PW1502	23a,b	Un	–
F00121a,b	PW1502	24a,b	BR04	–
F00122a,b	PW1502	25a,b	Un	–
F00123a,b	PW1502	26a,b	Un	–
F00124	PW1502	27	Un	–
F00125a,b	PW1502	28a,b	Un	Similar to BR02
F00126a,b	PW1502	29a,b	Un	–
F00127a,b	PW1502	30a,b	Un	Possible morphotype
F00128a,b	PW1502	31a,b	Un	–
F00129a,b	PW1502	32a,b	BR08	BR08 exemplar (Figs. 11D, 11E)
F00130a,b	PW1502	33a,b	Un	–
F00131	PW1502	34	BR06	Figure 10E
F00132a,b	PW1502	35a,b	Un	Possible galls
F00133a,b	PW1502	36a,b	Un	Unknown leaf damage, possibly fungal
F00134	PW1502	37SL	Un	Same level (SL) & lateral to PW1502
F00135	PW1502	38SL	Un	Same level (SL) & lateral to PW1502
F00136	PW1502	39	BR05	BR05 exemplar (Figs. 9E, 9F)
F00137a,b	PW1503	1a,b	BR02	BR02 exemplar (Figs. 6A–6C)
F00138	PW1503	2	Un	Several leaf fragments
F00139	PW1503	3	BR16	BR16 exemplar (Figs. 14E, 14F)
F00140a,b	PW1503	4a,b	BR01	Figures 5E, 5F; hole feeding (DT2)
F00141	PW1503	5	BR03, Un	–
F00142a,b	PW1503	6a,b	BR02	–
F00143	PW1503	7	Un	–
F00144a,b	PW1503	8a,b	Un	–
F00145a,b	PW1503	9a,b	BR18	BR18 exemplar (Fig. 15B); galls (DT11)
F00146a,b	PW1503	10a,b	Un	–
F00147a,b	PW1503	11a,b	Un	–
F00148a,b	PW1503	12a,b	Un	–
F00149a,b	PW1503	13a,b	BR06, Un	BR06 exemplar (Figs. 10A, 10B)
F00150	PW1503	14	BR02	Figure 6D
F00151	PW1503	15	BR03	Figure 7B
F00152	PW1503	16	BR03	–
F00153	PW1503	17	BR03	–
F00154	PW1503	18	BR12	BR12 exemplar (Figs. 13B, 13C); hole feeding (DT3)
F00155a,b	PW1503	19a,b	Un	Several leaf fragments
F00156	PW1503	20	BR01	Figure 5C
F00157a,b	PW1503	21a,b	BR03	–
F00158	PW1503	22	BR03	–
F00159	PW1503	23	Un	–
F00160	PW1503	24	BR03	–
F00161	PW1503	25	BR03	–
F00162	PW1503	26	Un	–
F00163a,b	PW1503	27a,b	BR03	–
F00164a,b	PW1503	28a,b	Un	Hole feeding (DT2)
F00165	PW1503	29	Un	Hole feeding (DT2)
F00166a,b	PW1503	30a,b	Un	–
F00167	PW1503	31	Un	Field photo only
F00168	PW1503	32	Un	Several leaf fragments
F00169a,b	PW1503	33a,b	Un	–
F00170a,b	PW1503	34a,b	BR01, BR03	–
F00171a,b	PW1503	35a,b	Un	Several leaf fragments
F00172	PW1503	36	BR03, Un	–
F00173	PW1503	37	Un	–
F00174	PW1503	38	BR03	–
F00175	PW1503	39	Un	–
F00176	PW1503	40	Un	–
F00177a,b	PW1503	41a,b	BR03	–
F00178	PW1503	42	Un	Several leaf fragments
F00179a,b	PW1503	43a,b	BR03	–
F00180	PW1503	44	Un	–
F00181	PW1503	45	Un	–
F00182	PW1503	46	Un	–
F00183	PW1503	47	BR03	Figures 7D, 7G
F00184a,b	PW1503	48a,b	Un	Several leaf fragments
F00185	PW1503	49	Un	–
F00186	PW1503	50	Un	–
F00187a,b	PW1503	51a,b	Un	–
F00188	PW1503	52	BR02, BR03	–
n/a	(PW1503)	(53,54)	(numbering gap)	(numbers skipped in the field)
F00189a,b,c	PW1503	55a,b,c	Un	–
F00190	PW1503	56	BR03 (2 leaves)	–
F00191	PW1503	57	BR03	–
F00192a,b	PW1503	58a,b	BR03, Un	Figure 7E; several leaf fragments
F00193a,b	PW1503	59a,b	Un	–
F00194	PW1503	60	Un	–
F00195	PW1503	61	BR01	–
F00196	PW1503	62	BR03	–
F00197	PW1503	63	Un	–
F00198	PW1503	64	BR03	–
F00199a,b	PW1503	65a,b	Un	Several leaf fragments
F00200	PW1503	66	Un	–
F00201	PW1503	67	BR01	–
F00202	PW1503	68	BR03	–
F00203a,b	PW1503	69a,b	Un	–
F00204	PW1503	70	Un	–
F00205a,b	PW1503	71a,b	Un	–
F00206	PW1503	72	Un	–
F00207	PW1503	73	Un	–
F00208	PW1503	74	Un (wood)	Wood sample
F00209	PW1503	75	BR03, Un	–
F00210a,b	PW1503	76a,b	Un	–
F00211a,b	PW1503	77a,b	BR03, Un	Several leaf fragments
F00212a,b	PW1503	78a,b	BR19	BR19 exemplar (Figs. 15C, 15D)
F00213	PW1503	79	Un	–
F00214	PW1503	80	Un	–
F00215	PW1503	81	BR03	–
F00216	PW1503	82	Un	–
F00217	PW1503	83	BR03	–
F00218	PW1503	84	Un	–
F00219a,b	PW1503	85a,b	BR13	Figure 13F
F00220a,b	PW1503	86a,b	Un	–
F00221	PW1503	87	BR03, Un	Several leaf fragments
F00222	PW1503	88	Un	Several leaf fragments
F00223	PW1503	89	BR03	–
F00224	PW1503	90	BR03	–
F00225	PW1503	91	Un	–
F00226	PW1503	92	Un	–
F00227	PW1503	93	Un	–
F00228	PW1503	94	BR03	–
F00229	PW1503	95	Un	–
F00230	PW1503	96	Un	=
F00231	PW1503	97	Un	–
F00232	PW1503	98	BR03	–
F00233	PW1503	99	Un	–
F00234	PW1503	100	BR03	–
F00235	PW1503	101	Un	–
F00236	PW1503	102	BR03	–
F00237a,b	PW1503	103a,b	BR23, Un	BR23 exemplar (Fig. 16E)
F00238	PW1503	104	Un	–
F00239	PW1503	105	Un	–
F00240	PW1503	106	Un	–
F00241	PW1503	107	BR03	–
F00242	PW1503	108	BR03, Un	Several leaf fragments
F00243	PW1503	109	Un	–
F00244a,b	PW1503	110a,b	Un	Likely dipterocarp. Hole feeding (DT2)
F00245	PW1503	111	BR01, Un	–
F00246	PW1503	112	BR03	–
F00247	PW1503	113	Un	Several leaf fragments
F00248	PW1503	114	BR03, Un	Several leaf fragments
F00249	PW1503	115	BR03, Un	Several leaf fragments
F00250	PW1503	116	BR03	–
F00251	PW1503	117	BR03	–
F00252	PW1503	118	BR03, Un	Several leaf fragments
F00253a,b	PW1503	119a,b	BR01	Figure 5A
F00254a,b	PW1503	120a,b	BR03	Elongate slot feeding (DT8)
F00255	PW1503	121	BR03 (2 leaves)	–
F00256	PW1503	122	Un	Several leaf fragments
F00257	PW1503	123	Un	Several leaf fragments
F00258a,b	PW1503	124a,b	Un	–
F00259	PW1503	125	BR03	–
F00260	PW1503	126	Un	Field photo only; potentially missing
F00261	PW1503	127	BR03 (2 leaves), Un	–
F00262	PW1503	128	Un	–
F00263	PW1503	129	BR20	BR20 exemplar (Figs. 15E, 15F)
F00264	PW1503	130	BR03	–
F00265	PW1503	131	BR03	–
F00266	PW1503	132	BR03	–
F00267	PW1503	133	Un	–
F00268	PW1503	134	BR03	–
F00269	PW1503	135	BR03	–
F00270	PW1503	136	BR03	–
F00271	PW1503	137	BR03	–
F00272a,b	PW1503	138a,b	Un	Several leaf fragments
F00273	PW1503	139	Un	–
F00274	PW1503	140	BR03, Un	Several leaf fragments
F00275a,b	PW1503	141a,b	BR06	Figure 10D
F00276	PW1503	142	Un	–
F00277	PW1503	143	BR03	–
F00278	PW1503	144	BR03	–
F00279	PW1503	145	Un	–
F00280	PW1503	146	Un	
F00281	PW1503	147	BR03, Un	Several leaf fragments
F00282	PW1503	148	Un	–
F00283	PW1503	149	Un	–
F00284	PW1503	150	Un	–
F00285	PW1503	151	Un	–
F00286	PW1503	152	BR03	–
F00287	PW1503	153	Un	–
F00288	PW1503	154	Un	–
F00289	PW1503	155	BR03	–
F00290	PW1503	156	Un	–
F00291	PW1503	157	Un	–
F00292	PW1503	158	BR03, Un	Figure 7C
F00293	PW1503	159	Un	–
F00294	PW1503	160	BR03, Un	–
F00295a,b	PW1503	161a,b	BR03	–
F00296	PW1503	162	Un	–
F00297	PW1503	163	BR03	–
F00298	PW1503	164	BR03	–
F00299	PW1503	165	Un	–
F00300	PW1503	166	BR02	–
F00301a,b	PW1503	167a,b	Un	–
F00302	PW1503	168	Un	–
F00303	PW1503	169	Un	–
F00304	PW1503	170	Un	Several leaf fragments
F00305	PW1503	171	Un	–
F00306	PW1503	172	BR03	–
F00307a,b	PW1503	173a,b	Un	–
F00308	PW1503	174	BR03, Un	Several leaf fragments
F00309a,b	PW1503	175a,b	BR03	–
F00310	PW1503	176	BR03, Un	Possible galls on one of several leaf fragments present, circular to polylobate, rim and center region of structures impressed into the matrix
F00311	PW1503	177	BR03	–
F00312	PW1503	178	BR03	–
F00313	PW1503	179	Un	–
F00314	PW1503	180	Un	–
F00315	PW1503	181	Un	–
F00316	PW1503	182	Un	–
F00317	PW1503	183	BR03	–
F00318	PW1503	184	Un	–
F00319	PW1503	185	BR03	–
F00320a,b	PW1503	186a,b	BR03	–
F00321	PW1503	187	BR03	–
F00322	PW1503	188	BR03	–
F00323	PW1503	189	BR03	Figure 7F
F00324	PW1503	190	Un	–
F00325	PW1503	191	BR03	–
F00326	PW1503	192	Un	–
F00327	PW1503	193	BR01, BR03	–
F00328	PW1503	194	Un	–
F00329	PW1503	195	BR03, Un	Several leaf fragments
F00330	PW1503	196	BR03	–
F00331	PW1503	197	BR03, Un	Several leaf fragments
F00332	PW1503	198	BR03	BR03 exemplar (Fig. 7A)
F00333	PW1503	199	BR03	–
F00334	PW1503	200	Un	–
F00335	PW1503	201	BR03	–
F00336a,b	PW1503	202a,b	BR13	BR13 exemplar (Figs. 13D, 13E)
F00337	PW1503	203	BR03	–
F00338a,b	PW1503	204a,b	BR24	BR24 exemplar (Fig. 16F)
F00339	PW1503	205	Un	–

**Note:**

UBDH and corresponding field numbers apply to individual slabs as collected in the field, each of which contains one or more fossils as indicated. Unidentified (Un) fragments are noted but not counted separately. See Table 1 for the morphotype list with “BR” codes, affinities, and totals. Localities PW1501 and PW1502 are at Berakas Beach, and locality PW1503 is at Kampong Lugu (Figs. 1–3). DT, damage type (Labandeira et al., 2007). UBDH, Herbarium of Universiti Brunei Darussalam.

## Additional Information and Declarations

### Field Study Permissions

The following information was supplied relating to field study approvals (*i.e*., approving body and any reference numbers): Fieldwork, fossil collecting, and specimen loans to the US were approved through permitting by several government ministries of Brunei Darussalam, including the Brunei Museum, the Ministry of Primary Resources and its Biodiversity Research and Innovation Centre (BRIC); and Universiti Brunei Darussalam (UBD), including the UBD Herbarium (UBDH) and Institute for Biodiversity and Environmental Research (IBER).

### Data Availability

The following information was supplied regarding data availability: The high-resolution image library of the macrofossil material is available open-access (CC-BY 4.0) at Figshare Plus, https://doi.org/10.25452/figshare.plus.16510584.
